# Post-myocardial Infarction Cardiac Remodeling: Multidimensional Mechanisms and Clinical Prospects of Stem Cell Therapy

**DOI:** 10.1007/s12015-025-10888-7

**Published:** 2025-05-05

**Authors:** Jiahui Yong, Jing Tao, Kaiyang Wang, Xia Li, Yining Yang

**Affiliations:** 1 Department of Cardiology, People’s Hospital of Xinjiang Uygur Autonomous Region, Ürümqi, Xinjiang China; 2 Xinjiang Key Laboratory of Cardiovascular Homeostasis and Regeneration Research, Ürümqi, Xinjiang China; 3 Department of Radiology, People’s Hospital of Xinjiang Uygur Autonomous Region, Ürümqi, Xinjiang China

**Keywords:** Stem Cell Therapy, Cardiac Remodeling, Myocardial Infarction, Mechanisms, Clinical Prospects

## Abstract

This review examines the application of stem cell therapy in myocardial remodeling following myocardial infarction, delving into the complex changes in the cardiac microenvironment after myocardial infarction, the potential mechanisms of stem cell treatment, and the progress of clinical research. It also provides an outlook on future research directions and clinical applications. After myocardial infarction, the heart undergoes a series of complex biological processes, including cardiomyocyte death and hypertrophy, activation and transdifferentiation of fibroblasts, remodeling of the extracellular matrix, functional changes in endothelial cells, and activation of inflammatory responses. These processes ultimately lead to pathological alterations in cardiac structure and function, known as cardiac remodeling. Stem cell therapy and its cell derivatives improve cardiac structure and function through multiple pathways, such as inducing myocardial regeneration, promoting angiogenesis, modulating the inflammatory microenvironment, and reducing fibrosis. However, stem cell therapy still faces many challenges in the treatment of myocardial infarction, such as low cell survival rates, excessive fibrosis, and low clinical translation efficiency. Despite these challenges, stem cell therapy, as an emerging treatment modality, shows great potential in cardiac remodeling after myocardial infarction. Therefore, this article, through its outlook on future research directions, emphasizes the importance of optimizing treatment strategies, developing new technologies, and conducting multicenter clinical trials, providing theoretical basis and practical guidance for the clinical application of stem cell therapy in myocardial repair after myocardial infarction.

**Graphical Abstract:**

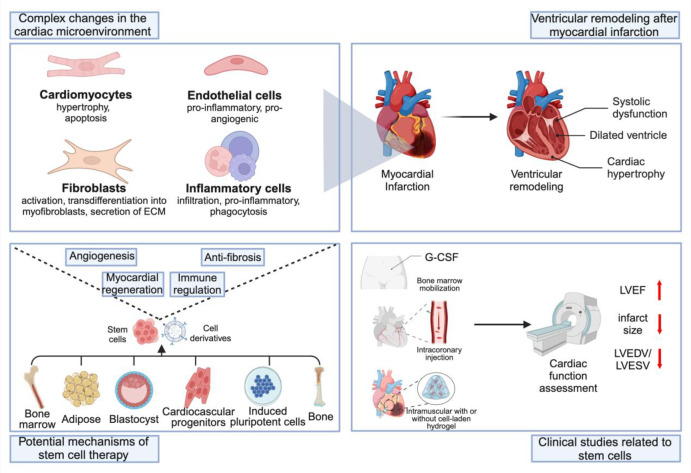

## Introduction

Myocardial infarction (MI) is a severe cardiac disease characterized by acute occlusion of the coronary arteries, leading to ischemic necrosis of cardiomyocytes. According to the 2023 report from the World Heart Federation, cardiovascular diseases (CVD) are one of the leading causes of death globally [1]. In China, the mortality rate of coronary heart disease among urban residents was 126.91 per 100,000 in 2020, while in rural residents it was 135.88 per 100,000. Notably, the mortality rate of MI increased from 2002 to 2020, with the mortality rate of acute myocardial infarction (AMI) among urban residents reaching as high as 78.65% in 2020, and 60.29% among rural residents [2]. After MI, the heart undergoes a series of complex biological processes that ultimately lead to pathological changes in cardiac structure and function. Specifically, these changes include ventricular dilation, myocardial hypertrophy, and contractile dysfunction, collectively termed ventricular remodeling (VR). VR is an adaptive response of the heart to injury following MI, but it often culminates in the development of heart failure.

VR is a complex, multi-stage, and dynamic process involving the interaction of various cell types and molecular mechanisms. Its core components include cardiomyocyte death, activation of the inflammatory response, recruitment and proliferation of myofibroblasts, remodeling of the extracellular matrix (ECM), and adaptive changes in the vascular network. In terms of temporal sequence, VR can be roughly divided into three stages: the inflammatory phase, the proliferative phase, and the maturation phase. From the perspective of remodeling scope, it can be further categorized into early and late remodeling. At the onset of VR, cardiomyocyte death due to ischemic injury acts as the trigger for the entire remodeling process. The damaged myocardial tissue, particularly the dead cardiomyocytes, releases a series of alarmins known as “danger-associated molecular patterns” (DAMPs), such as high-mobility group protein B1 (HMGB1). In addition, the injured tissue releases various chemokines, cytokines, heat shock proteins (HSPs), and components of the complement system, which together constitute the initiating signals for the inflammatory response. Stimulated by these signals, the inflammatory response is rapidly activated, involving the activation of both innate and adaptive immune cells. Neutrophils and macrophages are recruited and infiltrate the myocardial injury area, performing their phagocytic and clearance functions to remove dead cells and ECM debris from the infarcted region. This process not only clears the “debris” from the injured area but also creates conditions for the subsequent migration, proliferation, and activation of immune cells and fibroblasts. Based on the inflammatory response, further remodeling of cardiac tissue ensues. The interaction between cardiomyocytes, fibroblasts, and ECM becomes the core of remodeling, while changes in endothelial cell function also play a crucial role. Under the influence of various cytokines and growth factors, fibroblasts are transformed into myofibroblasts, which actively participate in ECM deposition and remodeling. Meanwhile, endothelial cells contribute to the remodeling of cardiac tissue by forming new blood vessels and repairing processes, providing necessary blood supply to the remodeling heart. These consecutive events not only reshape the structure of the heart but also profoundly affect its function. The ultimate outcome of VR, whether in terms of structural changes or functional impacts, is decisive for the clinical prognosis of patients.

Regeneration refers to the process of restoring the structure and function of damaged tissue through cell proliferation and differentiation. After MI, regeneration mainly involves cardiomyocyte regeneration and angiogenesis. Cardiomyocyte regeneration refers to the differentiation of stem cells into new cardiomyocytes to replace those that died due to infarction. Angiogenesis refers to the formation of new blood vessels in the damaged tissue to restore blood flow. Repair refers to filling the voids in damaged tissue and restoring tissue integrity through ECM remodeling and fibrosis. After MI, the repair process mainly involves the formation of scar tissue to prevent cardiac rupture. Cardiac regeneration and repair after MI involve multiple mechanisms, including activation of the inflammatory response, activation of fibroblasts, collagen deposition, recruitment and differentiation of stem cells, and ECM remodeling. These mechanisms interact with each other to promote the repair and functional recovery of the damaged myocardium.

For patients post-MI, regeneration and repair of the infarcted myocardium have significant clinical implications. Compared with traditional pharmacological treatments and surgical interventions, regenerative medicine approaches such as stem cell therapy have significant potential benefits. Stem cell therapy can comprehensively improve cardiac structure and function by promoting cardiomyocyte regeneration, angiogenesis, reducing fibrosis, and modulating immune responses. However, despite the great potential of stem cell therapy in the treatment of MI, it still faces many challenges. For example, low cell survival rates and excessive fibrosis limit the effectiveness of its clinical application. Therefore, in-depth research into the mechanisms of stem cell therapy, optimization of treatment strategies, and validation of its efficacy and safety in clinical settings are of great significance for advancing the treatment of post-MI myocardial remodeling.

This review aims to explore in depth the key roles and mechanisms of cardiomyocytes, fibroblasts, ECM, endothelial cells, and immune cells in the process of VR after MI. Additionally, this study focuses on analyzing the potential impact of stem cell therapy on ischemic myocardial repair and proposes new directions and prospects for myocardial repair after MI.

## Cardiac Remodeling after Myocardial Infarction

### Myocytes after Myocardial Infarction

During MI, necrosis of cardiomyocytes is the primary manifestation. This process is complex and severe, extending not only to the infarcted area but also to the adjacent border zone and potentially even to distant cardiomyocytes. Pathological insults such as tissue ischemia and hypoxia lead to a progressive loss of cardiomyocytes, accompanied by the disruption of the interstitial collagen framework (degradation of the ECM, discussed in detail later), ultimately resulting in localized thinning and dilation of the myocardium in the infarcted region [3].

From a biological perspective, cardiomyocytes are generally considered terminally differentiated cells with extremely limited proliferative capacity. Instead, cardiomyocytes primarily respond to various stimuli through changes such as hypertrophy, atrophy, or apoptosis. In the weeks to months following MI, surviving cardiomyocytes undergo a series of changes, the most notable of which is eccentric hypertrophy of cardiomyocytes in the non-infarcted area due to increased myocardial load, leading to dilation of the left ventricular cavity [4]. This process is initially compensatory, aiming to maintain cardiac output following infarcted myocardium and the resultant formation of non-compliant scar tissue. However, over time, these changes result in increased wall stress, left ventricular enlargement, and subsequent increases in left ventricular end-systolic and end-diastolic volumes, as well as elevated myocardial oxygen consumption, potentially expanding the area at risk for ischemia [5]. Persistent ventricular dilation also leads to hemodynamic abnormalities, including the development of ischemic or functional mitral regurgitation [6]. With increased left ventricular preload and thinning of the ventricular wall, the heart is unable to generate sufficient contractile force, resulting in further increases in end-systolic volume and a decline in left ventricular ejection fraction. This process is a central mechanism in the development of ischemia-driven dilated cardiomyopathy and a key factor in the progression to heart failure.

### Fibroblasts after Myocardial Infarction

Following MI, the mobilization and activation of reparative fibroblasts and myofibroblasts are essential for repairing the infarcted myocardial tissue. These cells synthesize and regulate the deposition of extracellular matrix proteins to prevent catastrophic events such as ventricular rupture. However, increasing evidence suggests that the role of fibroblasts extends beyond modulating fibrotic responses for myocardial repair; their reaction to inflammatory signals and regulation of the inflammatory response also have profound impacts on myocardial repair. A thorough understanding of the phenotypic transitions and functional changes of fibroblasts during myocardial repair after MI is a current priority in post-MI research and holds significant clinical implications for patients with MI or heart failure.

#### Fibroblasts during the Inflammatory Phase

Fibroblasts are considered the “sentinel cells” of myocardial injury. They are widely distributed in the cardiac interstitium, interact with cardiomyocytes, and serve as an important source of pro-inflammatory mediators. However, the precise role of fibroblasts in activating the post-infarction inflammatory cascade remains unclear.

Most evidence regarding the role of fibroblasts in post-infarction inflammation is descriptive. Studies have shown that in myocardial ischemia-reperfusion models, the production of reactive oxygen species (ROS) can activate inflammasomes in fibroblasts and promote the generation of interleukin-1β (IL-1β), thereby triggering leukocyte infiltration and cytokine expression [7]. Additionally, the expression of NLRP3 and IL-18 mRNA in the left ventricle is significantly increased, primarily localized to cardiac fibroblasts, especially when exposed to ATP. In *NLRP3*-deficient mice, cardiac function is significantly improved, and ischemic injury is reduced following ischemia-reperfusion [8]. This suggests that the NLRP3 inflammasome may be a key mediator through which fibroblasts exert their effects in myocardial tissue after MI.

Notably, the pro-inflammatory environment in the early post-infarction period has a significant impact on the phenotype and function of fibroblasts. For example, ROS can induce cardiac fibroblasts to produce pro-inflammatory signals while reducing ECM synthesis and enhancing the activity of matrix metalloproteinases (MMPs) [9]. Cytokine stimulation may also play an important role during the inflammatory phase of cardiac repair. Studies have shown that in animal models of cardiac ischemia-reperfusion, the oncostatin-M (OSM) receptor on the surface of cardiac fibroblasts can bind to OSM, synthesizing and releasing keratinocyte-derived chemokine (KC/CXCL1) and lipopolysaccharide-induced chemokine (LIX/CXCL5) through the JAK-STAT, MAPK, and PI3 K pathways. These factors are potent neutrophil chemoattractants [10]. IL-1α can promote the release of pro-inflammatory cytokines such as IL-1β, tumor necrosis factor-α (TNF-α), and IL-6 from cardiac fibroblasts, while also secreting the anti-inflammatory cytokine IL-10 [11]. In addition, IL-1β can inhibit transforming growth factor-β (TGF-β)-induced transformation of fibroblasts into myofibroblasts, reduce the expression of α-smooth muscle actin (α-SMA), and stimulate the synthesis of MMPs in an IL-1R1-dependent manner [12]. Beyond their pro-inflammatory effects, cytokine stimulation can also enhance fibroblast responsiveness to profibrotic signals. For example, following MI, increased levels of TNF-α and angiotensin II (Ang II) can induce the expression of the Ang II type 1 receptor (AT1) in fibroblasts, thereby increasing cell [3 H]-proline incorporation and tissue inhibitor of metalloproteinases-1 (TIMP-1) protein expression stimulated by Ang II, while reducing MMP-2 activity and expression [13].

The attenuation of the inflammatory response marks the transition of the repair process to the proliferative phase. However, whether fibroblasts act as anti-inflammatory cells during this process remains unclear. IL-1 receptor-associated kinase 3 (IRAK-3/IRAK-M) is an anti-inflammatory mediator expressed in the infarcted area, which can inhibit the inflammatory response following MI by reducing cellular responsiveness to pro-inflammatory signals, expressing decoy cytokine and chemokine receptors, and releasing soluble anti-inflammatory mediators [14]. In a mouse MI model, IRAK-M is significantly upregulated in fibroblasts and infiltrating macrophages, characterized by inhibition of fibroblast matrix-degrading properties and timely suppression of the inflammatory response. In contrast, *IRAK-M* knockout mice exhibit enhanced myocardial inflammation and protease activation, leading to adverse cardiac remodeling [15]. Given the dynamic changes in fibroblasts during myocardial repair and their responsiveness to inflammatory mediators, it can be hypothesized that fibroblasts may play an important role in suppressing post-infarction inflammation. However, the mediators and specific regulatory mechanisms involved require further investigation.

#### Fibroblasts during the Proliferative Phase

During the proliferative phase, fibroblasts emerge as the predominant cell type within the infarcted myocardium. Their key characteristics include high proliferative activity, migratory behavior through the temporary matrix network of the infarct region, transdifferentiation into myofibroblasts, and sustained synthesis of structural and extracellular matrix proteins.

#### Fibroblast-to-Myofibroblast Transdifferentiation

Under the dual influence of ECM degradation and mechanical stress, fibroblasts are stimulated by growth factors and cytokines to undergo phenotypic transformation into myofibroblasts [16]. Studies have shown that the infiltration of myofibroblasts in the infarct border zone is a hallmark of the transition from the infarct healing into the proliferative phase. These cells express contractile proteins, possess an extensive endoplasmic reticulum, and secrete large amounts of matrix proteins [17, 18]. The formation of myofibroblasts requires the synergistic stimulation of multiple factors, including the activation of TGF-β, deposition of ED-A fibronectin and matricellular proteins, and increased mechanical stress [19].

The TGF-β signaling pathway serves as a crucial link between the inflammatory and reparative responses. TGF-β is rapidly activated in the early stages of infarction, but the abundance of pro-inflammatory mediators at this time reduces cellular (especially fibroblast) responsiveness to TGF-β. As pro-inflammatory signals are attenuated, TGF-β robustly promotes myofibroblast transdifferentiation, activates matrix deposition, induces the expression of collagen and fibronectin, and upregulates the synthesis of protease inhibitors (e.g., TIMP-1) [20]. Studies have indicated that the profibrotic and matrix-depositing actions of TGF-β in fibroblasts are primarily mediated through the activation of the Smad3 signaling pathway, which also interacts with other key pathways regulating fibrogenic responses in the remodeling myocardium. Fibroblasts from *Smad3* knockout mice exhibit reduced α-SMA expression, decreased migratory activity, and diminished capacity for extracellular matrix protein synthesis. The TGF-β/Smad3/connective tissue growth factor (CTGF) pathway is a key mediator of these changes [21]. Additionally, TGF-β can mediate the nuclear translocation of myocardin-related transcription factor (MRTF)-A, thereby inducing α-SMA transcription in fibroblasts. Post-MI, MRTF-A responds to TGF-β1/ROCK signaling, directly binding to and activating the CArG elements on the Col1a2 promoter to promote fibroblast phenotypic transformation and the expression of collagen proteins such as Col1a1 and Col1a2 [22].

The regulation of the matrix environment plays a significant role in myofibroblast transdifferentiation. The extracellular matrix protein fibronectin ED-A is markedly upregulated in post-MI tissue. Studies have shown that *ED-A* knockout mice exhibit reduced left ventricular dilation and improved myocardial contractile function. At the tissue level, these mice display decreased inflammatory cell infiltration, reduced activity of MMP-2 and MMP-9, and diminished myofibroblast transdifferentiation [23]. Collagen deposition can also promote fibroblast transdifferentiation in the infarcted myocardium. Studies have shown that cardiac fibroblasts isolated and cultured on collagen matrices exhibit strong induction of myofibroblast transdifferentiation by type VI collagen. Further validation using in vivo MI models revealed concurrent increases in type VI collagen and myofibroblast content in the post-MI myocardium, suggesting an important role for the latter in cardiac remodeling [24]. Proteoglycans expressed by cardiac fibroblasts themselves can also influence myofibroblast transdifferentiation and fibrotic scar formation. Syndecan-4 (Syn4), a heparan sulfate proteoglycan, is significantly upregulated in post-MI tissue. *Syn4* knockout mice exhibit impaired myofibroblast transdifferentiation, with isolated fibroblasts showing reduced stress fibers and impaired activation of FAK, Akt, and RhoA pathways associated with cell migration and contractile function in response to fibronectin stimulation, as well as an increased incidence of cardiac rupture [25].

Direct evidence for the stimulatory effects of mechanical stress on fibroblasts post-MI is currently lacking, with most studies inferring the role of mechanical stress through descriptions of the unique functional changes in myofibroblasts. Studies have shown that under mechanical stress, fibroblasts acquire contractile stress fibers, thereby gaining the contractile capacity of myofibroblasts [26]. These intracellular microfilament structures, primarily composed of actin and myosin, play a crucial role in cellular contractile function. Moreover, stress fibers connect to the ECM through integrin-containing cell-matrix adhesion sites, remodeling the ECM [27]. In intact tissue, fibroblasts are typically protected by cross-linked ECM; however, disruption of the cardiac ECM’s structural integrity following tissue injury may expose fibroblasts to mechanical stress, thereby promoting their transformation into myofibroblasts. Myocardial hypertrophic growth resulting from pressure or volume overload is another manifestation of mechanical stress stimulation. Studies have shown an association between myocardial hypertrophy and myofibroblast transdifferentiation. In vitro experiments have demonstrated that mechanical stress mediates actin filament assembly through the Rho-Rho kinase-LIMK-cofilin pathway, which promotes the nuclear translocation of transcriptional coactivator MRTF-A and subsequent SMA promoter activation [28].

#### Fibroblast Proliferation

Numerous studies have confirmed that following MI, fibroblasts exhibit significant proliferative activity within the damaged tissue [29]. The regulation of fibroblast proliferative capacity involves several key molecules, including fibroblast growth factor 2 (FGF-2), TGF-β1, platelet-derived growth factor (PDGF), Angiotensin II (Ang II), and β-catenin [30]. Notably, activation of the TGF-β/Smad3 signaling pathway appears to convey an anti-proliferative effect [21].

In animal experiments, the absence of FGF-2 leads to a significant reduction in fibroblast proliferation and interstitial collagen deposition. *FGF2* knockout mice exhibit impaired scar contraction, ultimately resulting in increased infarct size and ventricular dilation [31]. Moreover, PDGF secreted by endothelial cells, through activation of the PDGF-B/PDGFR-α pathway, can reduce collagen deposition in the infarcted myocardium, possibly by inhibiting fibroblast proliferation [32]. Studies have shown that Ang II is a crucial regulator of cardiac fibrosis and extracellular matrix protein (e.g., collagen and fibronectin) synthesis in vivo, with one of its mechanisms potentially involving the G-protein-coupled AT1 receptor on cardiac fibroblasts [30]. In a rat MI model, injection of active β-catenin (Ad-catenin) into the infarct border zone significantly reduces infarct size, endowing cardiomyocytes and fibroblasts with anti-apoptotic properties and promoting cell entry into the proliferative cycle while increasing myofibroblast formation. In vitro experiments further confirmed that Ad-catenin primarily acts by increasing the number of fibroblasts [33]. In contrast, the TGF-β/Smad3 signaling pathway is significantly activated in the infarct border zone. In vitro studies have shown that *Smad*3 knockout fibroblasts exhibit significantly reduced collagen mesh contraction ability, migratory activity in response to serum stimulation, and ECM protein synthesis [21].

#### Fibroblast Migration

Studies have shown that in 5-lipoxygenase (*5-LOX*) knockout mice, collagen production and fibroblast accumulation are significantly reduced following MI, likely due to impaired migratory capacity of *5-LOX*^−/−^ fibroblasts [34]. During infarct repair, the specific migration of fibroblasts to the infarct area is crucial for the repair process. In addition to 5-LOX, pro-inflammatory cytokines such as IL-1β, cardiotrophin-1 (CT-1), and the TGF-β/Smad3 signaling pathway may also promote fibroblast migration [21, 35, 36]. It has been pointed out that quiescent cells typically reside in a “strong adhesion state,” whereas cells involved in post-injury repair and tissue remodeling are in an “intermediate adhesion state,” a process involving cell detachment. Matricellular proteins such as thrombospondin 1 (TSP1), TSP2, tenascin-C, and SPARC possess high regulatory activity and can induce cells to rapidly shift to an intermediate adhesion state, characterized by the loss of actin stress fibers and focal adhesions (e.g., vinculin and α-actin). Cell detachment may prevent apoptosis triggered by the loss of intercellular adhesion through the activation of survival signals [37]. IL-1β exerts a concentration-dependent pro-migratory effect on neonatal rat cardiac fibroblasts, with all three subfamilies of mitogen-activated protein kinases (MAPK)—extracellular signal-regulated kinase (ERK), c-Jun N-terminal kinase (JNK), and p38—being activated in response to cytokine stimulation [35]. CT-1 influences myofibroblast migration through multiple parallel signaling pathways, including altering membrane potential (initial depolarization followed by hyperpolarization), modulating intracellular Ca^2+^ levels (initial increase followed by decrease), and activating several intracellular signaling pathways, such as JAK2 activation and myosin light chain phosphorylation [36]. Additionally, the activation of migration-inhibitory signals has been observed in MI tissue. For example, the upregulation of CXC chemokine CXCL10/interferon-γ-inducible protein (IP)−10 in the infarcted myocardium inhibits growth factor-induced fibroblast migration [38], which may serve to prevent overly exuberant fibrotic responses.

#### Interregulation between Fibroblasts and the ECM

Numerous studies have demonstrated that myofibroblasts are the primary source of extracellular matrix proteins during MI repair. In addition to this, myofibroblasts regulate matrix homeostasis by promoting the deposition of matricellular proteins and by expressing MMPs and their inhibitors [39]. Several mediators have been identified as being associated with the acquisition of a synthetic phenotype by cardiac fibroblasts. For instance, TGF-β is one of the earliest identified molecules [40], with 10 ng/ml TGF-β1 stimulation shown to increase collagen I levels in non-operated control fibroblasts by 187 ± 10% [41]. Activated fibroblasts in the scar region can also secrete components required for the generation of angiotensin peptides, such as angiotensin-converting enzyme (ACE), the levels of which are significantly elevated in fibrous tissue within the infarct area [42]. The use of ACE inhibitors or AT1 receptor antagonists has been proven to have positive effects in reducing scar tissue metabolic activity and attenuating myocardial remodeling in non-infarcted regions [43]. However, the composition of the ECM also plays a crucial role in regulating fibroblast function and phenotypic changes, which will be described in the following section.

#### Fibroblasts during the Maturation Phase

The maturation phase is characterized by matrix cross-linking and a reduction in cellular components. Studies have shown that in mature infarct tissue, the density of myofibroblasts is significantly decreased, with large areas of myocardial tissue being replaced by collagenous scar in mouse models [44]. Apoptosis has been identified as the primary mechanism for the clearance of fibroblasts in mature scars [45], with matricellular proteins such as thrombospondin 1 (TSP1) potentially playing an important role in regulating fibroblast apoptosis [46]. Additionally, changes in matrix composition and the stable stress environment provided by the mature scar may prompt activated fibroblasts to transition to a quiescent state.

#### Fibroblasts in the Non-Infarcted Region

Studies have shown that while a large number of myofibroblasts undergo apoptosis, fibroblasts in the myocardium distant from the infarct region are chronically activated due to the effects of pressure and volume overload. However, research on the characteristics of fibroblasts in the non-infarcted region remains relatively limited. Animal experimental results indicate that pressure overload induces early activation of matrix synthesis pathways, which is associated with myocardial fibrosis and diastolic dysfunction, followed by activation of matrix degradation signals, leading to ventricular dilation and decompensated heart failure [47]. Volume overload, on the other hand, is primarily associated with matrix loss and cardiac dilation [48]. Further exploration is needed to elucidate the phenotypic and functional changes of fibroblasts in this particular and important region.

### Extracellular Matrix after Myocardial Infarction

The cardiac ECM is crucial for maintaining the structural integrity of the heart. Disruption of the ECM network leads to alterations in ventricular geometry and is closely associated with both systolic and diastolic dysfunction. Studies have found that in the context of MI, the composition of the cardiac ECM may undergo the most dramatic changes [49].

In the infarct region, myofibroblasts accumulate in large numbers and produce substantial amounts of extracellular matrix proteins. This collagen-based matrix ultimately forms a mature scar that provides mechanical support to the infarcted heart. In the later stages of healing, defects in ECM components alter the mechanical properties of the heart, leading to enhanced ventricular dilation and spherical changes, known as post-MI remodeling. Additionally, extracellular matrix proteins act as key regulators of the repair response by modulating cell-cell interactions. Extracellular matrix components also play important roles in inflammatory pathways and regulate fibroblast phenotype and gene expression, thus playing a significant role in cardiac repair.

### ECM during the Inflammatory Phase

#### Activation and Function of MMPs

During the inflammatory phase, damaged myocardial tissue releases a series of inflammation-inducing molecules, such as TNF-α and IF-1β, which promote the release and activation of MMPs [50]. Studies have shown that in mouse infarct models, IL-1 stimulates the synthesis of MMPs in an IL-1R1-dependent manner [12]. The rapid activation of MMPs leads to the degradation of the cardiac ECM. In rats subjected to left coronary artery ligation, activation of MMPs is observed in the cardiac interstitium as early as 6 h to 2 days post-infarction [39]. Collagenases cleave collagen into 75 and 25 kDa gelatin fragments, which are further degraded into amino acids and oligopeptides by gelatinases (such as MMP2 and MMP9) and serine proteases [51]. This process involves two aspects: first, in the infarct region, pre-existing latent collagenases (e.g., MMP1) are activated during the repair process, which is an important part of the repair mechanism; second, as the existing collagenases and gelatinases are activated and participate in collagen degradation, their overall levels decrease, triggering the synthesis of new collagenase mRNA to maintain the dynamic balance of collagen degradation during tissue repair [39].

#### Formation of the Temporary Matrix and its Inflammatory Role

In mouse and canine models of MI, it has been observed that in the early stages of MI, the extravasation of plasma proteins leads to the formation of a complex, fibrillar temporary matrix, primarily composed of fibrin and plasma fibronectin. Subsequently, the temporary matrix derived from plasma proteins is dissolved by proteases produced by granulation tissue cells and is rapidly replaced by a cell-derived temporary matrix, which contains cellular fibronectin and hyaluronan, thus forming the temporary matrix of the inflammatory phase.

Recent studies have found that fibrinogen-derived fragments, as important components of the temporary matrix, make significant pathological contributions to myocardial ischemic injury. In the Langendorff myocardial ischemia-reperfusion model, mice lacking fibrinogen exhibited significantly reduced infarct size. Administration of a fibrin-derived peptide Bβ15–24 to ischemic mice competes with fibrinogen-derived fragment N-terminal disulfide bond-II (similar to fibrin E1 fragment) for binding to vascular endothelial cadherin, thereby preventing leukocyte infiltration into ischemic myocardium and reducing infarct size [52]. In a clinical study involving 234 patients with acute ST-segment elevation myocardial infarction (STEMI), patients were administered a naturally derived fibrin peptide FX06 or a corresponding placebo via intravenous injection during percutaneous coronary intervention. The results showed that although there was no significant difference in infarct size between the FX06 group and the control group, the area of the necrotic core region on magnetic resonance imaging (MRI) was significantly reduced [53]. Additionally, studies have indicated that in a skin wound model, animals lacking fibrinogen exhibited reduced tensile strength and delayed wound closure in skin wounds, but the final skin repair was still completed with results comparable to the control group [54]. However, in the MI repair process, reduced scar tensile strength may lead to a series of adverse effects, such as enhanced left ventricular remodeling and significant ventricular dysfunction. Therefore, the important role of fibrinogen in MI repair still requires further exploration.

#### ECM during the Proliferative Phase

During the proliferative phase, pro-inflammatory signals are attenuated, while profibrotic signaling is significantly enhanced in the damaged myocardial tissue, accompanied by the generation of new blood vessels. ECM remodeling during this stage is of great significance for tissue repair and regeneration.

#### Role of Fibronectin

During the proliferative phase, fibronectin secreted by fibroblasts and macrophages constitutes a “second-order” provisional matrix. The deposition of fibronectin plays a crucial role in the transdifferentiation and activation of myofibroblasts. The transdifferentiation of myofibroblasts is the result of the combined action of multiple factors, including mechanical tension, TGF-β, and the ED-A splice variant of cellular fibronectin [55, 56]. TGF-β can induce the expression of α-SMA, thereby promoting the transdifferentiation of myofibroblasts. Additionally, TGF-β enhances the synthesis of extracellular matrix molecules, including ED-A fibronectin (FN), which is a fibronectin isoform specifically expressed during wound healing and fibrosis [57, 58]. Studies have shown that application of the anti-ED-A monoclonal antibody IST-9 to rat skin wounds can specifically block the TGF-β-induced upregulation of α-SMA and type I collagen expression, indicating that ED-A FN plays an important role in TGF-β signaling and is essential for TGF-β-induced myofibroblast phenotypic transformation [56]. Fibronectin also plays an important role in the organization and stabilization of the matrix. It dynamically polymerizes to form fibrillar structures within the ECM, thereby maintaining the structure and function of the ECM [59]. For example, in vitro cell experiments have shown that fibronectin polymerization is essential for the deposition of type I collagen and TSP-1, providing a framework for these molecules to be correctly positioned within the ECM. Moreover, fibronectin is important for maintaining the composition of cell-matrix adhesion sites. Studies have found that in the absence of fibronectin, specific integrins (e.g., α5β1) and protein tyrosine phosphatases (e.g., tensin) cannot localize to fibroblast-matrix adhesion sites, which are key areas for cell-ECM interactions [60]. However, the specific significance of these interactions in cardiac repair has not been fully explored. Additionally, the deposition of laminin and hyaluronan in the infarct region is closely related to angiogenesis and cell migration. Specifically, in the early stages of MI, laminin appears fragmented in the infarct region, indicating disruption of the cardiomyocyte basement membrane; however, laminin immunoreactivity is observed in the basement membrane of newly formed blood vessels in the infarct region 7 to 14 days after reperfusion [61]. The formation of new blood vessels provides key oxygen and nutrients for the formation and maturation of the matrix.

#### Role of Matricellular Proteins

Matricellular proteins are a diverse group of extracellular matrix proteins with unique functions. Unlike traditional structural proteins, they do not directly participate in the scaffold construction of the ECM but instead regulate tissue homeostasis and remodeling by modulating cell functions and activities under specific physiological or pathological conditions. Studies have shown that various matricellular proteins are significantly upregulated during the proliferative phase after MI and play key regulatory roles in cardiac repair.

#### Tenascin-C

Tenascin-C is a matricellular protein that is significantly upregulated during the proliferative phase. During the proliferative stage following MI, Tenascin-C is primarily secreted by fibroblasts and localized to the edges of the infarct region, remodeled myocardial areas, and around so-called “hibernating myocardial cells” [62]. By 2 to 3 weeks post-infarction, Tenascin-C expression gradually shifts from the edges to the center of the infarct region, and it almost completely disappears during the maturation phase [62]. Studies have shown that various cytokines and growth factors, such as TNF-α, TGF-β, basic fibroblast growth factor (bFGF), and Ang II, can stimulate the synthesis of Tenascin-C through paracrine or autocrine pathways [63]. In vitro experiments further revealed the regulatory effects of Tenascin-C on cardiomyocytes. Tenascin-C significantly increases the attachment of cardiomyocytes to laminin but reduces costameric attachment of cardiomyocytes to laminin while increasing non-costameric attachment [64]. Costameres are key structures connecting cardiomyocytes to the surrounding ECM, transmitting the contractile force of myofibrils to the ECM through protein complexes, which is crucial for normal cardiomyocyte function. Costameric attachment is characterized by striated dark contacts in the region where cardiomyocytes contact the culture substrate, helping cardiomyocytes maintain structural integrity during contraction and transmit contractile force to the ECM to maintain cardiac pumping function. However, during tissue remodeling after MI, surviving cardiomyocytes in the infarct border zone need to relax this attachment to rearrange cells. Meanwhile, cells maintain attachment to the matrix through non-costameric white or gray contact sites to cope with the contractile pressure of the myocardial scar later [65]. Therefore, Tenascin-C may promote tissue remodeling by regulating the adhesion of surviving cardiomyocytes in the infarct border zone. Additionally, studies using a mouse model of myocardial cryoinjury combined with in vitro cell models found that Tenascin-C promotes the recruitment of myofibroblasts in myocardial repair by stimulating cell migration and differentiation. The alternatively spliced fibronectin type III (FNIII) and fibrinogen (Fbg)-like domains in Tenascin-C are considered key mediators of this process [66]. However, the specific role of Tenascin-C in post-infarction repair remains unclear.

#### TSP-1

TSP-1 is another matricellular protein that is significantly upregulated in experimental MI models. In a canine MI reperfusion model, TSP-1 protein was significantly present in the ECM, microvascular endothelial cells, and a subset of monocytes in the infarct border region 5 to 28 days after reperfusion. In *TSP-1* knockout (*TSP-1*^−/−^) mice with infarction, significant upregulation of chemokines, including monocyte chemoattractant protein-1 (MCP-1), macrophage inflammatory protein-1α, and interferon-γ-inducible protein-10/CXCL10, as well as cytokines IL-1β, IL-6, and TGF-β, was observed, indicating enhanced post-infarction inflammatory response and prolonged inflammatory phase. Moreover, in *TSP-1*^−/−^ mice, the density of macrophages and myofibroblasts in the infarcted and adjacent non-infarcted myocardial scar regions was significantly increased, suggesting that granulation tissue formation extended into the non-infarcted region [67]. These results suggest that selective endogenous expression of TSP-1 in the infarct border region may protect non-infarcted myocardium from fibrotic remodeling by inhibiting the inflammatory response and limiting the expansion of granulation tissue. However, the specific anti-inflammatory mechanisms of TSP-1 require further investigation.

#### Osteopontin (OPN)

Osteopontin (OPN) has been found to be significantly expressed in human post-MI tissue. Studies have shown that OPN mRNA and protein expression is abundant in macrophages within the necrotic and granulation tissues after MI [68]. OPN plays an important role in MI healing and cardiac remodeling. Research has shown that *OPN*-deficient mice exhibit increased left ventricular dilation, characterized by increased left ventricular end-diastolic diameter (LVEDD) and left ventricular end-systolic diameter (LVESD), while ejection fraction (EF) and percentage fractional shortening (%FS) are significantly reduced. Additionally, activation of MMPs helps reduce myocardial fibrosis and left ventricular dysfunction in *OPN*-deficient mice [69, 70]. These results indicate that OPN exerts a cardioprotective effect by promoting collagen synthesis and deposition to mitigate adverse myocardial remodeling. However, the specific mechanisms by which OPN mediates matrix remodeling effects remain unclear.

#### Secreted Protein, Acidic and Rich in Cysteine/Osteonectin (SPARC/Osteonectin)

SPARC/Osteonectin is a matricellular protein that regulates cell-matrix interactions during wound healing. Its expression is also significantly upregulated during MI repair. Studies have found that *SPARC* gene-targeted inactivation in mice results in a fourfold increase in mortality after MI, mainly due to a significant increase in the incidence of cardiac rupture and heart failure. Moreover, the infarct region in *SPARC* gene knockout mice exhibited disorganized granulation tissue and immature collagen scar. Administration of TGF-β rescued cardiac rupture in mice. In vitro experiments showed that silencing SPARC with shRNA in cardiac fibroblasts significantly weakened TGF-β-mediated Smad2 phosphorylation [71]. These results suggest that SPARC can prevent adverse cardiovascular events after MI and may play a key role through the TGF-β signaling pathway. However, whether SPARC’s cardioprotective effects involve other key mechanisms remains to be explored.

#### Periostin

Periostin is a matricellular protein that is highly expressed in the infarct border region of human and mouse myocardium. Researchers constructed a *periostin*-deficient (*periostin*^−/−^) mouse model and found that these mice had impaired cardiac repair after acute MI, leading to cardiac rupture. The results indicated that this was due to reduced recruitment of *α-SMA*-positive cells, impaired collagen fiber formation, and decreased focal adhesion kinase (FAK) phosphorylation, thereby reducing myocardial stiffness [72]. Moreover, *periostin*^−/−^ mice that survived exhibited reduced fibrosis and improved cardiac function. Mechanistic studies found that cardiac fibroblasts isolated from *periostin*^−/−^ mice had reduced adhesion to cardiomyocytes, and deposition of type V collagen was significantly decreased [73]. Type V collagen plays a key role in regulating collagen fibril diameter and alignment, affecting collagen fiber growth and structure [74]. The above results suggest that periostin may play a role in cardiac repair by inducing myofibroblast recruitment and collagen synthesis.

#### ECM during the Maturation Phase

Studies have shown that in a canine model of MI, collagen content continuously increases from 7 days to 6 weeks post-MI. During the first 7 days following MI, the heart is characterized by ventricular dilation and expansion of the infarct size. However, from day 7 to 6 weeks post-MI, the scar gradually contracts, the infarct area progressively diminishes, and the ventricular wall thins, a phase defined as the maturation period [75]. During scar maturation, the tensile strength of the myocardium significantly increases, a change closely associated with the cross-linking intensity of collagen. It has been found that compared to the control group, the activity of lysyl hydroxylase in infarcted tissue significantly increases and peaks in the later stages of infarction. This heightened enzyme activity promotes the formation of the final mature scar [76]. However, the enhanced tensile strength also passively increases myocardial stiffness, which may subsequently lead to impairments in both diastolic and systolic cardiac function [77]. Additionally, the mature matrix network may shield fibroblasts from mechanical tension, thereby facilitating cell deactivation and entry into a quiescent state [17, 26].

In summary, the components of the ECM are not merely passive byproducts of pathological changes in the infarcted heart but actively regulate inflammatory responses and the repair process by transmitting signals that influence cell survival, phenotypic transformation, and gene expression. The dynamic changes in the ECM not only affect cellular functions but also impact the overall structure and function of the tissue by modulating cell-matrix interactions. A deeper understanding of the mechanisms by which the ECM functions in MI repair will facilitate the exploration of more effective therapeutic targets and strategies, thereby improving cardiac function and prognosis following MI.

### Endothelial Cells and the Microvasculature after Myocardial Infarction

MI inflicts severe damage on the coronary microvasculature, leading to the disintegration of blood vessels in the infarcted area and a paucity of capillaries. This injury manifests primarily in two aspects: On one hand, the death of cardiomyocytes and endothelial cells in the core of the infarct leads to intramyocardial hemorrhage, a hallmark of severe microvascular damage; on the other hand, there is a significant increase in microvascular permeability. As myocardial ischemia persists, a robust tissue repair response—angiogenesis—begins to emerge. Studies have shown that an appropriate angiogenic response is closely associated with favorable outcomes in animal models of MI, characterized by reduced scar size, attenuated cardiac remodeling, and improved cardiac function. However, the exact mechanisms remain incompletely understood, with current research mainly focusing on the infiltration of inflammatory cells and the facilitation of material exchange to meet the high metabolic demands, thereby limiting further damage to various cellular components within the microenvironment from ischemic injury [78]. Other studies have also demonstrated that intercellular communication between endothelial cells and other cell types during infarct repair plays an important role in improving cardiac function [79]. What is certain is that the dynamic changes in endothelial cells are of significant importance in the post-MI healing process.

#### Overall Overview of Endothelial and Microvascular Injury after MI

Microvascular injury, along with microvascular occlusion caused by distal arterial thromboembolism and blood cell blockage, is the main manifestation of ischemia/reperfusion injury in the coronary microcirculation. Although endothelial cells can withstand brief ischemic injury, prolonged ischemia or reperfusion following ischemia can lead to irreversible damage. In a rat model, it was found that significant vascular leakage occurred in the infarcted area after 90 min of ischemia, indicating increased vascular permeability [80]. At the microscopic level, capillary endothelial cells exhibited pronounced cytoplasmic blebbing and an increase in the number of caveolae. The increase in caveolar vesicles is considered a key mediator of trans-endothelial transport and also acts as a shear sensor for endothelial cells, leading to endothelial cell proliferation mediated by ROS following a sharp reduction in blood flow [81]. Additionally, the nuclei showed some early signs of apoptosis, such as DNA condensation at the nuclear periphery, which are considered manifestations of endothelial cell activation. The vascular integrity of the microcirculation largely depends on the connections between endothelial cells, and it was found that the average number of connections per capillary in the infarcted area was significantly reduced [80].

Studies have shown that the increase in microvascular permeability is regulated by secretory proteins rapidly produced in the infarcted area under hypoxic conditions. These secretory proteins include: Vascular Endothelial Growth Factor A (VEGFA), Angiopoietin-like 4 (ANGPTL4), and Angiopoietin 1 and 2 (ANGPT1, ANGPT2). VEGFA is an important member of the VEGF family and plays a dominant role in the regulation of angiogenesis. However, it has been found that VEGFA can also increase microvascular permeability by activating Src tyrosine kinase and disrupting the vascular endothelial growth factor receptor 2 (VEGFR2)/cadherin-5 complex at endothelial cell junctions [82]. In contrast, ANGPTL4 can antagonize the effect of VEGFA on endothelial cell permeability. It has been found that in *ANGPTL4* knockout mice and mice treated with recombinant ANGPTL4, ANGPTL4 can stabilize the VEGFR2/cadherin-5 complex, thereby maintaining the structural integrity and barrier function of endothelial cells in the first few hours after reperfusion [83]. Moreover, in patients with AMI, low serum ANGPTL4 concentration after percutaneous coronary intervention (PCI) is associated with microvascular injury and obstruction as shown by cardiac magnetic resonance imaging (cMRI) [84]. ANGPT1 and ANGPT2 play opposing roles in regulating microvascular integrity and inflammation after MI. Endothelial cells produce ANGPT2 under hypoxic and pro-inflammatory cytokine induction, which has been shown to interfere with ANGPT1-tyrosine kinase with immunoglobulin-like and epidermal growth factor-like domains 2 (TIE2) signaling after MI, thereby promoting vascular leakage and inflammation [85]. Antibody-mediated neutralization of ANGPT2 has been shown to reduce the final scar size and improve left ventricular remodeling in mice after MI [86].

Enhanced proliferative activity is another important characteristic of endothelial cells after MI. Based on three-dimensional (3D) reconstruction studies of porcine coronary vessels, it was found that the number of microvessels in the infarcted area decreased, and the width and surface area of the vascular network for oxygen and nutrient exchange were reduced, with the capillary network becoming sparse. These changes were more pronounced on day 7 compared to day 1 after MI. However, from the perspective of individual microvessels, the radius and surface area of microvessels significantly increased on day 7 after MI, while vessel length shortened [87]. Further observations of the diameter of residual microvessels after MI revealed that on day 7 post-MI, the percentage of microvessels with a diameter greater than 8.2 μm (i.e., arterioles/venules) and medium-sized capillaries (6.9 and 8.2 μm) in the infarcted area increased, while smaller-diameter vessels decreased. This indicates that microvessels in the infarcted area underwent regression, while others expanded or even underwent arteriolarization. A similar observation was made in the study by Susanne et al. Based on the theory that the formation of medium and large vessels is the result of active endothelial cell proliferation, they observed BrdU-positive flat cells lining the inner walls of blood vessels, demonstrating the proliferative activity of endothelial cells [88].

The above studies demonstrate that within 7 days after MI, the microvascular network in both infarcted and non-infarcted areas exhibits adaptive manifestations of reduced integrity and selective survival. However, existing ischemic and MI animal models still have limitations and may not fully replicate the pathophysiological characteristics of humans. Importantly, findings that endothelial cells exhibit significant apoptosis and impaired intercellular connections have deepened the understanding of the key role of endothelial cells in the MI microenvironment.

#### Dynamic Changes of Endothelial Cells after MI

With the continuous development of transcriptome sequencing technology, studies have gradually delineated the dynamic changes of endothelial cells after MI through single-cell RNA sequencing. The latest research, through bulk RNA sequencing, analyzed the transcriptomic characteristics of mouse cardiac endothelial cells at three key time points after injury (D0: before injury; D2: day 2 post-MI; D7: day 7 post-MI; D28: day 28 post-MI). The findings revealed that on day 2 post-MI (D2), endothelial cells exhibited increased gene expression related to metabolism, involving enzymes associated with glucose metabolism and nucleotide biosynthesis. Concurrently, genes related to cell proliferation were significantly upregulated, particularly those associated with the transition from the S phase to the G1 phase of the cell cycle. Additionally, stress and cell death-related genes, such as hypoxia-inducible factor 1-alpha (*HIF-1α*) and Caspase 3, were upregulated, and endothelial cells acquired a pro-inflammatory phenotype, characterized by significant upregulation in the expression of pro-inflammatory chemokines (e.g., chemokine (C-C motif) ligand 2 [Ccl2] and Ccl7) and cytokines (e.g., Il6). By day 7 post-MI (D7), during the proliferative phase, the transcriptomic profile of endothelial cells underwent significant changes. At this time, genes associated with cell division were upregulated, indicating that cells entered the mitotic phase (M/G2 phase). Meanwhile, the expression of immune cell binding proteins (e.g., vascular cell adhesion molecule [Vcam1] and intercellular adhesion molecule [Icam1]) and chemokines was increased. In parallel, genes related to vasoconstriction and vascular tone were downregulated, involving calcium signaling pathways and contractile protein-related genes. Moreover, endothelial cells acquired pro-angiogenic and mesenchymal characteristics, as evidenced by the upregulation of ECM synthesis and deposition-related genes (e.g., collagen type I alpha 1 chain [*Col1a1*] and *Postn*). On day 28 post-MI (D28), endothelial cells had not yet returned to their pre-infarction state. Functional analysis showed that cells retained pro-inflammatory, pro-thrombotic, and pro-fibrotic properties [89]. Further research by Lukas et al. revealed that on day 3 post-MI, endothelial cells exhibited increased expression of S phase-related genes, which returned to homeostasis by day 14. Additionally, from day 3 to day 7 post-MI, endothelial cells transiently displayed mesenchymal cell characteristics, with increased expression of genes such as *Col3a1*, *Fn1*, and serpin family E member 1 (*Serpine1*), while the expression of the endothelial cell marker gene claudin-5 (*Cldn5*) was significantly reduced and returned to baseline levels 14 days post-injury [90]. This activation of a mesenchymal state may induce angiogenesis by promoting endothelial cell migration and clonal expansion. Moreover, this subpopulation of endothelial cells may secrete ECM and inflammatory proteins, thereby indirectly influencing wound healing responses and fibrosis. The above studies demonstrate that the transcriptome of endothelial cells undergoes significant stage-specific changes after MI, providing a systematic and dynamic characterization of the dynamic changes in endothelial cells post-MI.

In combination with genetic lineage tracing techniques, studies have further revealed the adaptive transcriptional and phenotypic changes of endothelial cells in response to the damaged microenvironment, including alterations in pro-inflammatory, metabolic, mesenchymal, and hematopoietic characteristics. This plasticity of endothelial cells in responding to acute stress is crucial for preventing adverse outcomes.

#### Inflammatory Chemotaxis of Endothelial Cells after MI

During the inflammatory response following MI, the chemotaxis, recruitment, and infiltration of inflammatory cells are key features of the cardiac microenvironment. Studies have shown that functional changes in endothelial cells play an important role in the recruitment and infiltration of inflammatory cells. Endothelial cells can respond to pro-inflammatory environments and support immune cell infiltration through specific mechanisms. Specifically, endothelial cells release Weibel-Palade bodies containing P-selectin through degranulation, thereby promoting the adhesion and rolling of inflammatory cells [91]. Additionally, endothelial cells can be induced to express ICAM1 and VCAM1. ICAM1 interacts with activated leukocyte β2 integrins to firmly adhere white blood cells to the endothelial layer. The subsequent trans-endothelial migration of leukocytes depends on the synergistic action of ICAM1, VCAM1, and junctional adhesion molecules (JAM) [92]. In the infarcted myocardial tissue, upregulation of pro-inflammatory cytokines (e.g., TNF, IL-1β, and members of the IL-6 family) induces the synthesis of endothelial cell adhesion molecules [93]. These cytokines not only promote the expression of endothelial cell adhesion molecules but also induce endothelial cell apoptosis and ROS production, while reducing the biosynthesis of nitric oxide (NO), overall leading to endothelial layer instability [94]. Moreover, the loosening of endothelial cell contacts is thought to facilitate the recruitment of inflammatory cells, which also provides benefits for the role of inflammatory infiltration in post-MI repair.

#### Metabolic Changes of Endothelial Cells after MI

Compared with other cardiac cells, endothelial cells have relatively fewer mitochondria and do not rely on mitochondrial oxidative phosphorylation to produce ATP under normal physiological conditions. This unique metabolic characteristic makes endothelial cells more resistant to ischemic and injury conditions than cardiomyocytes [95]. The metabolic profile of endothelial cells is primarily centered on fatty acid transport, with CD36 and fatty acid-binding proteins (such as fatty acid-binding protein 4 [FABP4] and FABP5) being endothelial-specific proteins involved in fatty acid metabolism [96]. Under ischemic conditions, the induction of hypoxia-inducible factors and oxygen-sensing components (such as endothelial nitric oxide synthase [eNOS] or NADPH oxidase) significantly enhances glycolysis in cells, while fatty acid metabolism is correspondingly reduced [97]. Concurrently, the expression of glucose transporter 1 (GLUT1) and phosphofructokinase B3 (PFKB3) is significantly increased [98], while the expression of the transcription factor forkhead box O1 (FOXO1) is suppressed [99]. Additionally, hypoxia causes glutamine metabolism to shift from an oxidative pathway to a reductive carboxylation state [100]. Hypoxia-induced metabolic changes also involve AMP accumulation, which can activate AMP-activated protein kinase (AMPK). AMPK plays an important role in regulating fatty acid oxidation and NO production in endothelial cells and affects endothelial cell function by modulating inflammatory and angiogenic responses [101]. However, whether endothelial cells in MI models also involve changes in these metabolic pathways and molecular components requires further validation.

#### Endothelial-Mesenchymal Transition in Endothelial Cells after MI

In recent years, the development of single-cell RNA sequencing technology has provided a new perspective for studying the dynamic changes of endothelial cells after MI and revealed that endothelial cells can transiently exhibit a mesenchymal phenotype shift (endothelial-mesenchymal transition, EndMT) after MI. This phenomenon has sparked researchers’ keen interest in the specific components that induce functional changes in endothelial cells. Macrophages, as one of the important cell populations in myocardial repair after MI, have been found to significantly upregulate the expression of MMP14 in macrophages on day 7 post-MI. MMP14 can activate latent TGF-β1, thereby enhancing the transmission of the TGF-β1/Smad2 signaling pathway in endothelial cells and inducing the occurrence of EndMT [102]. Further in vitro experiments have shown that the inflammatory environment induced by IL-1β and the profibrotic environment of TGF-β2 can synergistically strongly promote the EndMT process. In this co-stimulation environment, nuclear factor-κB (NF-κB) plays a key regulatory role [103]. In addition, the metabolic state of endothelial cells also has a significant impact on the EndMT process. It has been found that the knockout of carnitine palmitoyltransferase II (Cpt2E) in endothelial cells leads to reduced fatty acid oxidation, increased levels of cytoplasmic acetyl-CoA, and affects the synthesis and secretion of silent information regulator T1 (SIRT) and the cell cycle-related gene MYC proto-oncogene (MYC), all of which have been proven to have an important impact on the EndMT process [104–106]. Mitochondrial dysfunction and cellular oxidative stress are also important inducers of EndMT. It has been found that increased levels of intracellular ROS and the knockdown of oxidative stress inhibitory molecules such as hydrogen sulfide (H2S) can induce the occurrence of EndMT [107, 108]. However, whether these effects still exist after MI and their specific mechanisms in the in vivo environment are not yet clear.

#### Pro-Angiogenic Role of Endothelial Cells after MI

The generation of new blood vessels after MI is crucial for the repair and functional recovery of damaged myocardium. Through single-cell RNA sequencing, it has been found that resident endothelial cells in the infarct border region exhibit a significant proliferative phenotype after MI. This cell population replenishes the endothelial cells lost due to ischemic injury through clonal proliferation, thereby promoting angiogenesis. Plasmalemma vesicle-associated protein (Plvap) has been identified as a key molecule regulating endothelial cell proliferation [109]. However, despite the formation of new blood vessels in the infarcted area, their function and structure are not complete. Analysis using a fluorescence imaging system revealed that although the newly formed blood vessels in the infarcted area are morphologically well-formed, they suffer from insufficient perfusion, structural disorganization, and lack of distinct vascular hierarchy. Specifically, this is manifested as reduced vessel diameter and increased branching. Moreover, capillary endothelial cells exhibit characteristics of insufficient vascular maturation, such as reduced coverage of neural antigen 2 *NG-2*^+^ pericytes and type IV collagen [110]. These features indicate that although new blood vessels are formed, their maturation and functionalization processes are hindered, which may affect the efficiency and quality of tissue repair after MI.

Despite numerous studies revealing the dynamic changes of endothelial cells after MI and the transcriptional profiles of endothelial cells at different stages post-MI provided by single-cell RNA sequencing, it is still not possible to fully integrate these dynamic changes with the spatiotemporal sequence of angiogenesis. Therefore, we cannot yet clearly articulate the specific temporal order of endothelial cell dynamic changes. The resolution of this issue requires further basic research to validate and demonstrate the temporal sequence of endothelial cell dynamic changes.

### Inflammatory Response after MI

Years of research have demonstrated that the inflammatory response plays a crucial role in the repair and remodeling processes following myocardial injury. Beginning with ischemic damage triggered by coronary occlusion, inflammation rapidly mobilizes nearly all cell types within the cardiac microenvironment, including mast cells, monocytes, cardiomyocytes, fibroblasts, and endothelial cells, and induces the large-scale recruitment and activation of neutrophils and monocytes. These cells are not only involved in the clearance and repair of the infarcted area but also have a profound impact on cardiac remodeling and functional recovery. Over time, the inflammatory response transitions from the acute phase to the reparative phase, accompanied by changes in cell types and functional adjustments. Precise regulation of this process is essential for achieving optimal healing outcomes, as excessive or insufficient inflammatory responses can lead to adverse consequences, such as poor myocardial healing or even cardiac rupture. A deeper exploration of the mechanisms of inflammation in MI is of great significance for understanding the key links in disease progression and developing effective therapeutic strategies.

#### Inflammatory Response and the Innate Immune System

Shortly after the onset of ischemia, resident mast cells release preformed inflammatory mediators such as histamine and TNF-α through degranulation [108]. Concurrently, necrotic myocardial tissue cells release alarmins, which further activate the innate immune system in a process mediated by “danger-associated molecular patterns” (DAMPs) [111, 112]. Alarmins are a diverse group of endogenous signaling molecules that are predominantly released following tissue necrosis and promote the activation and recruitment of innate immune cells by binding to pattern recognition receptors (PRRs) [113]. Among these, HMGB1 is a key initiator of the inflammatory response following myocardial ischemia. HMGB1 activates downstream pro-inflammatory cascades by interacting with transmembrane receptors such as Toll-like receptors (TLRs) and the receptor for advanced glycation end products (RAGE) [114]. Subsequently, resident macrophages and cardiomyocytes begin to produce inflammatory cytokines and chemokines, which participate in the recruitment of innate immune cells.

#### Role of Chemokines in the Inflammatory Response

In the infarcted myocardial tissue, the expression of two major families of chemokines is significantly increased: the CC and CXC chemokines. CXC chemokines containing the tripeptide sequence Glu-Leu-Arg rapidly increase in the infarcted myocardium and mediate the recruitment of neutrophils [10]. CC chemokine ligand 2 (also known as MCP-1) and CCL7 are rapidly upregulated in the infarcted myocardium and mediate the recruitment of pro-inflammatory phagocytic monocytes, which clear dead cells and matrix debris from the infarcted area [115]. Additionally, interactions involving the CC chemokine receptor type 5 (CCR5) may be involved in the recruitment of monocyte subsets with anti-inflammatory properties, such as suppressive monocyte subsets and regulatory T cells [116]. Pro-inflammatory macrophage subsets also infiltrate the infarcted area and may help maintain a pro-inflammatory environment within the infarcted myocardium [117].

#### Role of Cytokines in the Inflammatory Response

In animal models of MI, the expression of pro-inflammatory cytokines TNF, IL-1β, and IL-6 is significantly increased. TNF, which is released following MI, can promote inflammatory damage and induce the synthesis of chemokines and adhesion molecules in the infarcted myocardium [118]. However, TNF has also been noted to protect cardiomyocytes from apoptosis [119]. The differential signaling through TNF receptor 1 and 2 may regulate remodeling in the infarcted heart [120]. IL-1β has been confirmed to mediate the recruitment and activation of leukocytes while delaying the activation of myofibroblasts [12, 121]. Additionally, neutralization of IL-1 has been found to reduce cardiomyocyte apoptosis both in vitro and in vivo [122]. The upregulation of IL-6 in the infarcted myocardium may regulate inflammation and repair through the IL-6 receptor subunit β and activation of the JAK/STAT signaling cascade [123, 124]. Neutrophil adhesion is almost entirely dependent on CD18 and ICAM-1. Animal studies have shown that recombinant IL-6 can significantly induce the adhesion of yeast activation serum (ZAS)-stimulated neutrophils to cardiomyocytes and the expression of ICAM-1 on cardiomyocytes during ischemia-reperfusion [125]. However, it has been found that knockout of *IL-6* in the infarcted myocardium can be compensated by other family members, rendering the absence of IL-6 inconsequential to post-infarction myocardial function and remodeling [126].

#### Other Inflammatory Activation Factors

In addition, other components within necrotic myocardial tissue can also activate immune responses. For instance, HSPs and ATP released from necrotic cells can activate immune cells. The damaged ECM may also transmit key signals that activate innate immune cells. Low molecular weight hyaluronan and fibronectin fragments can activate Toll-like receptors (such as cell surface receptors TLR2 and TLR4) and serve as important initiators of pro-inflammatory signaling. MMPs produced by cardiac fibroblasts can degrade the ECM, thereby facilitating cell migration to the infarcted area [127]. This process not only aids in the infiltration of inflammatory cells but may also impact cardiomyocyte survival. ECM degradation is associated with cardiomyocyte dislocation, which can lead to cardiomyocyte death as cardiomyocytes lose key survival signals transmitted by the ECM [128]. Activation of the complement system is also involved in transmitting immune responses in infarcted myocardial tissue, and complement inhibition can reduce leukocyte recruitment post-MI. Ischemia-mediated production of ROS is crucial for inflammatory signaling in the infarcted myocardium, promoting leukocyte chemotaxis and infiltration through the activation of all steps in the recruitment of inflammatory cells [129]. Substances like ROS can also activate inflammasomes in fibroblasts, leading to the production of IL-1β, which triggers leukocyte infiltration and cytokine expression [7]. These processes further amplify the inflammatory response and exacerbate myocardial injury. Additionally, non-cardiomyocyte components within cardiac tissue, such as venules endothelial cells, also play an important role in the inflammatory response. Studies have found that in MI models, venules endothelial cells can release CXC chemokines, such as IL-8 (angiogenic factor) and IP-10 (angiostatic factor) [130]. These chemokines not only induce the recruitment of neutrophils but also trigger the release of MCP-1, promoting monocyte migration [131]. Collectively, these events induce the large-scale production and recruitment of neutrophils and monocytes, which are considered independent risk factors for adverse outcomes following MI.

After accumulating in the heart, neutrophils and monocytes actively participate in the inflammatory cascade. Initially, their primary function is to clear dead components, such as dead and dying cardiomyocytes. Myeloid-epithelial-reproductive tyrosine kinase (MerTK) in macrophages is a necessary component for the clearance of apoptotic cardiomyocytes by macrophages [132]. Type I interferons and interferon regulatory factor 3 (IRF3) have been shown to be another important component in inducing and clearing apoptotic cardiomyocytes. Studies have found that MI triggers the transformation of cardiac macrophages into an “interferon-inducible cell (IFNIC)” macrophage subtype, while activating the intracellular IRF3-interferon axis, thereby promoting inflammatory cell infiltration and cardiac dysfunction [133]. Interestingly, apoptotic cardiomyocytes play a crucial role in this process. Experiments have shown that during MI, apoptotic cardiomyocytes release double-stranded DNA (dsDNA) to activate the cytoplasmic DNA sensor cyclic GMP-AMP synthase (cGAS) in macrophages, which in turn promotes the synthesis of cyclic GMP-AMP (cGAMP). cGAMP binds to the stimulator of interferon genes (STING) protein (a transmembrane protein located in the endoplasmic reticulum) and activates the TANK-binding kinase 1 (TBK1)-dependent IRF3, thereby promoting the production of IFN-β. Macrophages induce apoptosis in cardiomyocytes by secreting inflammatory factors such as IFN-β and CXCL10. H-151, a selective inhibitor of STING protein, has been found to reduce IFN-β production in macrophages, thereby alleviating cardiomyocyte apoptosis and fibroblast activation [134]. These immune cells can produce proteases that help digest necrotic myocardial tissue and further release inflammatory cytokines such as IL-1, IL-6, and TNF, amplifying the inflammatory response through their effects on leukocytes, endothelial cells, and cardiomyocytes. As time progresses post-MI, the number of neutrophils gradually decreases and almost completely disappears by day 7, while monocytes continue to accumulate in the ischemic heart. They primarily originate from extramedullary hematopoiesis in the spleen and are continuously recruited to the damaged myocardium, where they differentiate into cardiac macrophages and influence the inflammatory process [135].

#### Proliferative Phase and Inflammatory Inhibition Signals

The repair of damaged tissue relies on the timely inhibition and effective control of the inflammatory response. During the proliferative phase, the inflammatory response gradually subsides, and tissue repair mechanisms are initiated. The main characteristics of this stage are the disappearance of neutrophils and the appearance of Ly6 C(low) macrophages. Studies have shown that almost all cell types in the cardiac microenvironment, as well as a series of pro-inflammatory cytokines, growth factors, and chemokines, are involved in the transition from inflammation to the repair process [136].

Macrophages, as key inflammatory cells, participate in tissue remodeling during the proliferative phase. Research has found that the phenotype of macrophages in the infarcted myocardium undergoes the most significant dynamic changes, indicating a shift from early pro-inflammatory M1-type Ly6 C(high) macrophage infiltration to a predominance of late reparative M2-type Ly6 C(low) macrophages [137]. Nuclear receptor subfamily 4 group A member 1 (NR4 A1) has been shown to promote the transition of macrophages from a pro-inflammatory phenotype to a reparative phenotype by inhibiting the expression of IL-6, TNF, and MMP9 [138]. Regulatory T cells (Treg cells) can induce the differentiation of M2-type macrophages and promote the activation of myofibroblasts as well as the expression of monocyte/macrophage-derived proteins, thereby facilitating repair in the infarcted area [139]. Additionally, neutrophils can induce macrophages with high phagocytic capacity for apoptotic cells by releasing neutrophil gelatinase-associated lipocalin (NGAL) [140]. In the myocardial infarction mouse model lacking *IRAK-M*, enhanced inflammatory responses, increased protease activation, and increased infiltration of CD11-b+/Ly6 C(high) pro-inflammatory monocytes were observed, while the matrix-degrading activity of fibroblasts was significantly weakened [15]. These reparative macrophages promote fibrosis and angiogenesis by producing factors such as TGF-β, VEGF, and myeloid-derived growth factor (MYDGF) [134, 141].

Moreover, the efficient clearance of apoptotic cells by phagocytes can activate anti-inflammatory signals, facilitating the transition from the inflammatory response to the repair process. For example, the absence of *MerTK* leads to the accumulation of apoptotic cardiomyocytes, slowed clearance of inflammatory cytokines, and reduced cardiac contractile function [132]. IL-1 receptor-associated kinase 3 (IRAK-3/IRAK-M), an anti-inflammatory mediator secreted by fibroblasts and macrophages in the infarct zone, inhibits the inflammatory response following MI by reducing cellular responsiveness to pro-inflammatory signals, expressing decoy cytokines and chemokine receptors, and releasing soluble anti-inflammatory mediators [14]. The transition from inflammation to repair is also accompanied by the accumulation of mast cells, which peaks on day 7 post-infarction. However, inflammatory inhibition does not appear to be the primary role of mast cells. Studies have shown that mast cells can protect cardiac contractile function by promoting the phosphorylation of tropomyosin I and myosin-binding protein C through tryptase-induced protein kinase A activity [142].

Endothelial cells also play an important role in this process. They attract pericytes and smooth muscle cells by secreting PDGF-B. The interaction of PDGF-B with PDGFR-α and PDGFR-β exerts fibrotic effects. It has been found that activation of the PDGF-B/PDGFR-β pathway in infarcted myocardium mediates the formation of a new vascular coverage cell layer, promotes vascular maturation, and inhibits the infiltration of inflammatory cells. Activation of the PDGF-B/PDGFR-α pathway, although it does not affect vascular maturation, can work synergistically with the former to reduce collagen deposition and inhibit fibrosis [32]. A few weeks later, the inflammatory regulation and pro-repair functions of neutrophils, monocytes, mast cells, and various lymphocyte subsets gradually weaken, and the number of immune cell populations gradually returns to homeostasis levels.

Notably, studies have indicated that immune cells may also be involved in ventricular remodeling in non-infarcted areas. Previous research has shown that heart failure following MI may primarily be due to remodeling of the left ventricular wall in non-infarcted areas. In mouse models, researchers found that recruitment of monocytes also occurs in the remote myocardium from the infarct, even significantly increasing and peaking on day 10 post-ischemia. This phenomenon is mediated by both local macrophage proliferation and monocyte recruitment, likely activated by chemokines such as MCP-1 and the MAPK pathway, which is activated by increased wall tension due to ventricular remodeling [143, 144]. The above studies demonstrate that the inflammatory response plays an important role in multiple stages following MI, including remodeling of the remote myocardium from the infarct. Further exploration of the mechanisms mediating immune cell accumulation in this area and its prognostic implications will provide new insights for the treatment of MI.

## Stem Cell Therapy for Myocardial Infarction

The term “stem cell” is commonly used to describe a population of undifferentiated cells that have the potential to differentiate into various specific cell types and possess the ability to self-renew. To date, researchers have isolated several stem cell populations with cardiogenic potential, including human adult stem cells and pluripotent stem cells (PSCs).

### Pluripotent Stem Cells

PSCs are stem cells that have the ability to differentiate into any cell type in the body, and they mainly include embryonic stem cells (ESCs) and induced pluripotent stem cells (iPSCs).

ESCs are primarily derived from the inner cell mass of embryos that have developed to the blastocyst stage (approximately 5–6 days) during in vitro fertilization and are the earliest studied type of PSCs. ESCs have extremely high differentiation potential and can theoretically differentiate into any tissue cell type in an adult animal. Additionally, ESCs can proliferate indefinitely in vitro while maintaining an undifferentiated state [145]. Boheler et al. first reported the potential of ESCs to generate functional cardiomyocytes (CMCs) in vitro [146]. Studies have shown that VEGF, bone morphogenetic protein (BMP), and FGF2 may induce cardiogenesis and differentiation of ESCs by activating signaling pathways such as phosphoinositol 3-kinase (PI3 K) IA class, protein kinase C (PKC), and Wnt/β-catenin [147]. However, since the source of ESCs is related to early embryos, their research involves ethical issues, which limits the conduct of research to some extent.

iPSCs are a type of pluripotent stem cell generated by reprogramming somatic cells (such as skin cells or blood cells) through genetic reprogramming techniques to acquire properties similar to ESCs. This technology was first reported in 2006 by Japanese scientist Shinya Yamanaka, who reprogrammed adult cells into a pluripotent state by introducing specific transcription factors (such as Oct4, Sox2, Klf4, and c-Myc) [148]. iPSCs have the ability to self-renew and differentiate into cells of all three germ layers. Compared with ESCs, iPSCs can avoid ethical controversies and can be prepared from the patient’s own cells, reducing the possibility of immune rejection [149]. Numerous studies have shown that iPSCs can successfully differentiate into functional CMCs and vascular endothelial cells [150] and form functional three-dimensional cardiac tissue [151]. However, the direct implantation of undifferentiated ESCs and iPSCs has been found to cause teratoma formation, which is another important limitation for the clinical application of these cells.

### Human Adult Stem/Progenitor Cells (SPCs)

SPCs are a class of cells with self-renewal ability and differentiation potential (tissue-specific) that exist in mature tissues. These cells may originate from stem cell populations left over from embryonic development to maintain tissue homeostasis and repair damaged tissues [152]. Among them, bone marrow-derived mononuclear cells (BM-MNCs), mesenchymal stem cells (MSCs), and cardiovascular progenitor cells (CPCs) have been proven to be useful for improving myocardial injury after MI [153].

BM-MNCs and MSCs are the most commonly used stem cells in clinical research promoting cardiac tissue regeneration. BM-MNCs are a population of mononuclear cells isolated from bone marrow, including hematopoietic stem cells (HSCs), bone marrow mesenchymal stem cells, and endothelial progenitor cells (EPCs). BM-MNCs can be obtained through bone marrow aspiration, do not require extensive expansion, and can be separated by density gradient centrifugation. They are the “first-generation stem cells” used in cardiovascular disease clinical trials. Studies have shown that BM-MNCs can directly differentiate into vascular endothelial cells and smooth muscle cells to form new blood vessels [154]; or secrete pro-angiogenic factors and endothelial cell growth factors in an autocrine/paracrine manner to promote blood vessel growth [155]. With the continuous progress in the understanding of stem cell biology and cell separation techniques, researchers have begun to use purified progenitor cell populations, such as MSCs [156] and CD34^+^ cells.

MSCs originate from the mesoderm and can be isolated from various tissues such as bone marrow, adipose tissue, placenta, umbilical cord, and even dental pulp. In addition to their self-renewal and multidifferentiation capabilities, MSCs have been widely studied for their low immunogenicity and strong paracrine and immunomodulatory functions. Studies have shown that VEGF, stromal cell-derived factor-1 (SDF-1), bFGF, and C-X-C chemokine receptor type 4 (CXCR4) and their downstream signaling can actively stimulate the survival, proliferation, and differentiation of MSCs [157], which is of great significance in cardiac tissue regeneration. Moreover, the lack of major histocompatibility class II markers in MSCs makes them an excellent choice for allogeneic transplantation [158].

HSCs and EPCs are cells that specifically express CD34 and CD133. Among them, EPCs are recruited to the peripheral blood after injury and are called circulating progenitor cells when isolated from the blood. Circulating progenitor cells can differentiate into blood cell lineages and endothelial cells and have pro-angiogenic effects [159]. CD34^+^ cells are progenitor cells with the potential to differentiate into endothelial cells. In vitro, they can proliferate and form tubular structures during culture and exhibit functions of endothelial cells, such as the uptake of acetylated low-density lipoprotein (acLDL) and response to molecular stimuli like acetylcholine. In animal models, CD34^+^ cells have also been shown to integrate into newly formed blood vessels in ischemic tissues and form vascular walls together with host vascular endothelial cells [160].

The paracrine/autocrine activity of MSCs and BM-MNCs is considered an important mechanism for promoting cell survival, growth, and eventual differentiation into myocardial tissue. In particular, MSCs require in vitro expansion and thus cannot be used on the same day of collection like BM-MNCs, which may limit their application as autologous transplants for the treatment of acute diseases. Adipose tissue-derived regenerative cell populations are obtained from liposuction samples, and their stromal fraction contains multipotent cells that can differentiate into multiple lineages. These cells can also be administered on the same day of collection and have been shown to contain a higher proportion of MSCs and CD34^+^ cells.

CPCs are the only recognized stem cells with cardiomyogenic potential that appear during embryonic development and play an important role in cardiac development, repair, and regeneration [161]. Studies have shown that after transplantation into the infarcted heart, CPCs can not only replenish damaged cardiomyocytes but also differentiate into vascular smooth muscle cells and vascular endothelial cells, thereby promoting capillary formation, improving blood circulation in the infarcted area, and facilitating repair of the infarcted myocardium [162].

Cortical bone-derived stem cells (CBSCs) are a subtype of mesenchymal stem cells derived from the cortical bone, with the ability to self-renew and differentiate into multiple lineages, mainly into chondrocytes and osteoblasts. However, they have stronger proliferative capacity and lineage differentiation stability. Importantly, studies have found that CBSCs can promote the repair of damaged hearts in the following ways: (1) secreting pro-angiogenic factors to stimulate endogenous angiogenesis; (2) differentiating into functional mature cardiomyocytes and vascular cells [163]; and (3) exerting immunomodulatory effects [164]. Additionally, it has been found that mouse cortical bone-derived stem cells (mCBSCs) can secrete high levels of TGF-β1, which promotes the self-migration of mCBSCs and the activation and differentiation of fibroblasts into myofibroblasts [165], contributing to tissue repair and scar maturation after MI. CBSCs have also been shown to produce cytokines and growth factors that promote the growth, chemotaxis, differentiation, and survival of T cells and macrophages, such as TIMP1, CXCL12, and macrophage colony-stimulating factor (M-CSF). CXCL12 strongly attracts lymphocytes [166] and protects cardiomyocytes from apoptosis [167]. M-CSF induces the differentiation of bone marrow cells into macrophages, induces an immunosuppressive phenotype, and induces the production of CCL2, which acts as a potent chemoattractant for lymphocytes and plays a key role in infarct healing [115]. Experiments in a porcine MI model have also shown that intracardiac injection of allogeneic CBSCs can reduce pathological remodeling of myocardial structure and function and improve left ventricular functional reserve [168].

### Mechanisms of Stem Cell Therapy in Post-Infarction Cardiac Remodeling

Stem cell therapy holds the promise of reversing myocardial damage and improving cardiac function, a potential that starkly contrasts with most existing medical therapies, which can only alleviate cardiovascular symptoms. Over the past 20 years, more than 200 stem cell clinical trials targeting cardiovascular diseases have been conducted, with the majority of cell types demonstrating good safety profiles. However, the rapid translation of clinical trials has largely outpaced our understanding of their mechanisms of action. Given that myocardial injury may progressively lead to severe ventricular remodeling over time, it is crucial to delve into the key mechanisms by which stem cell therapy affects myocardial remodeling.

Studies have found that implanted stem cells can promote regeneration of damaged cardiac tissue through three main mechanisms: (1) secretion of various paracrine or autocrine factors into the damaged cardiac microenvironment; (2) induction of proliferation and differentiation of endogenous CSPCs; and (3) generation of new CMCs and vascular endothelial cells (VECs) in the damaged area. This study primarily discusses the effects and mechanisms of non-cardiac-derived stem cells on ventricular remodeling after MI.

#### Stem Cell Therapy and Myocardial Regeneration

Necrosis and apoptosis of cardiomyocytes are among the earliest and most severe pathological changes following MI. Traditionally, cardiomyocytes have been considered terminally differentiated cells with little to no capacity for further division and proliferation. Consequently, non-contractile scar tissue gradually replaces the damaged myocardial tissue in the infarcted area, severely compromising the functional and structural integrity of the damaged region. Replenishing lost cardiomyocytes has long been the primary goal of stem cell therapy for MI. Although subsequent research has revealed that cardiomyocyte regeneration is not the main mechanism of stem cell therapy, the potential of different types of stem cells to differentiate into cardiomyocyte lineages and their applications have still been widely recognized and validated.

Currently, various stem cell lineages have been used to differentiate or generate cardiomyocyte-like cell (CLCs) types, including BMSCs, umbilical cord-derived mesenchymal stromal cells, adipose-derived mesenchymal stromal cells, human embryonic stem cells, human induced pluripotent stem cells, and resident cardiac stromal cells (characteristics and distinctions of CLCs derived from different sources are detailed in Table 1). Among them, MSCs are the most extensively studied type of stem cells.

**Table 1:** Advantages and disadvantages of differentiating CLCs from various stem cell sources

Cell type	Advantages	Disadvantages	References
BM-MSCs	Low risk of immune rejection: lack of surface markers	Limited differentiation capacity: low efficiency in differentiating into cardiomyocytes	[169, 170]
	Possess immunomodulatory properties	High passage number in vitro culture affects proliferation and differentiation	
	Easily accessible, widely available	Tumorigenic risk: potential for tumor formation	
Umbilical Cord Stem Cells (UCSCs)	Low risk of immune rejection: lack of surface markers	Potential for thrombosis: associated with coagulation cascade	[171]
	Possess immunomodulatory properties	Tumorigenic risk: potential for tumor formation	
	Easily accessible, no risk to mother or infant		
Adipose-Derived Stem Cells (ADSCs)	Low risk of immune rejection: lack of surface markers	High passage number in vitro culture affects proliferation and differentiation	[169, 170]
	Possess immunomodulatory properties	Tumorigenic risk: potential for tumor formation	
	Easily accessible, widely available		
	Anatomically close to myocardium, potentialy similar genetic lineage		
Human Embryonic Stem Cells (hESCs)	Capable of differentiating into all three germ layers, high differentiation potential	Ethical issues: involves embryo destruction	[169, 172]
	Can produce a higher proportion of cardiomyocytes	Risk of immune rejection: potential for host immune response	
		Genomic instability: potential for genetic mutations in differentiated cells	
Human Induced Pluripotent Stem Cells (hiPSC)	Capable of differentiating into all three germ layers	Low differentiation efficacy: limited cardiomyocyte yield	[169, 172]
	Fewer ethical issues	Genomic instability: potential for genetic mutations during induction	
	Can be induced from patient’s own cells, reducing immune rejection		

#### CLCs Derived from BMSCs

BMSCs can be specifically induced to differentiate into CLCs under in vitro drug induction or in vivo myocardial microenvironment simulation conditions, exhibiting functional characteristics of cardiomyocytes. Numerous components have been shown to promote the differentiation of BMSCs into cardiomyocytes, including chemical agents (such as 5-azacytidine [5-aza]), miRNAs, cytokines (such as insulin-like growth factor 1 [IGF-1]), and members of the TGF-β superfamily.

Wakitani et al. first reported the in vitro differentiation of MSCs into cardiomyocytes, primarily through the use of the cytidine analog 5-aza. Studies have found that MSCs typically contain a methylated region that determines myogenic transformation, which is transcriptionally inactive under normal conditions. 5-aza is a demethylating agent that can bind to repressor proteins on the promoters specific for cardiomyocyte differentiation, causing demethylation and thereby initiating the differentiation of stem cells into cardiomyocytes [173]. Under the stimulation of 5-aza, research has observed structures similar to cardiomyocytes [174]. Xing et al. found that the combined use of 5-aza and Ang II can induce the expression of cardiomyocyte proteins (such as cTnI) and α-actin, and proposed that it may function by promoting the production of TGF-β1 [175], although the specific mechanism has not been elucidated. However, despite the promising role of 5-aza in stem cell differentiation, its potential carcinogenicity may limit its application in therapy [176].

MiRNAs are small non-coding RNAs approximately 22 nucleotides in length that act as negative regulators of gene expression by binding to the 3’ untranslated region (3’UTR) of mRNA. Studies have shown that miRNAs play an important role in promoting the differentiation of BMSCs into cardiomyocytes. For example, miR-1 can promote cardiomyogenic differentiation of stem cells by targeting Delta-like 1 (Dll-1, a Notch ligand), as evidenced by increased expression of cardiomyocyte-specific genes (such as cTnI, cTnT, and myosin heavy chain 6 [MYH6]) [177]. miR-1–2 has been shown to promote the differentiation of BMSCs into cardiomyocytes by activating the Wnt/β-catenin signaling pathway [178]. However, the specific mechanisms in both pathways have not been clearly demonstrated. Gain- and loss-of-function experiments with miR-124 have shown that miR-124 can influence the differentiation of BMSCs into cardiomyocytes by targeting the signal transducer and activator of transcription 3 (STAT3)/GATA-4 pathway, with miR-124 leading to inhibition of cardiomyogenic differentiation and reduction of cardiomyocyte potassium channel currents [179]. It is worth noting that although BMSCs treated with miRNAs can express cardiac-specific genes, these cells still lack the morphological characteristics of cardiomyocytes, indicating the need for further functional studies of BMSC-derived CLCs as a basis for stem cell therapy.

Cytokines, as important paracrine factors, also play a significant role in the differentiation of stem cells into CLCs. Studies have shown that the combined application of IGF-1 and hepatocyte growth factor (HGF) can inhibit apoptosis of BMSCs after implantation and increase angiogenesis. Notably, levels of cardiac-specific markers (such as cTnT, GATA binding protein 4 [GATA4], NK2 homeobox 5 [NKx2.5], and connexin 43 [Cx43]) in BMSCs are also increased [180]. This synergistic effect has been validated in a porcine MI model, where intracoronary infusion of IGF-1 and HGF was found to significantly increase the number of c-kit^+^ endogenous cardiac stem/progenitor cells (epCSCs) in the infarcted area and promote the formation of new myocardium (including cardiomyocytes and microvessels) in the infarcted and surrounding areas [181]. IGF-1 itself has been shown to induce the differentiation of BMSCs into CLCs through the PI3 K/Akt and MAPK/ERK pathways [182]. bFGF is a heparin-binding growth factor that is thought to induce the differentiation of BMSCs into cardiomyocytes during embryogenesis, although it is not essential for cardiomyocyte development. However, the combination of bFGF and the steroid drug hydrocortisone can induce the differentiation of sternal BMSCs into cardiomyocytes, with significant upregulation of cTnI, cTnC, and Cx43 in cell cultures [183]. The role of bFGF in post-MI repair has also been validated in a canine MI model, where retrograde coronary infusion of bFGF and BMSCs, compared to infusion of BMSCs alone, resulted in increased cell implantation efficiency, enhanced vascular differentiation, and significant thickening of the ventricular wall [184]. This suggests that this novel combination therapy and delivery method may be a promising strategy for cardiac repair following ischemic injury. IL-1, a member of the pro-inflammatory cytokines, has been shown to be significantly expressed after myocardial injury. Studies have found that IL-1β also has a positive effect on cardiac differentiation in vitro, characterized by significant increases in cardiomyocyte-related protein expression, and its combination with 5-aza (5 ng/ml) has been shown to be more effective than single-agent treatment [185]. However, despite the significant expression of cardiomyocyte-related molecules in BMSCs treated with the above cytokines, the functional characteristics of these cells have not been fully demonstrated. The addition of functional studies of cells will be the next direction of research on the role of cytokines in the differentiation of stem cells into CLCs.

TGF-β1 and BMP belong to the TGF-β superfamily and are involved in regulating multiple biological processes, including cell proliferation, survival, differentiation, and migration. Studies have shown that under the induction of TGF-β1, BMSCs significantly increase the expression of cardiac-specific markers. This effect is even more pronounced when treated with autologous serum, and the cell proliferation rate is also increased [186]. Moreover, TGF-β1 has been proven to potentially participate in the differentiation of BMSCs into cardiomyocytes induced by electrical stimulation [187]. Research has found that after treating BMSCs with BMP2, BMP2 shows the ability to enhance the differentiation of BMSCs into CLCs, both in terms of cellular ultrastructural characteristics and the expression of cardiomyocyte-specific proteins [188]. As key molecules in tissue growth and development, validating the role of the TGF-β superfamily in post-MI myocardial remodeling in humans will significantly enhance our understanding of cell therapy.

In addition to the aforementioned regulatory components, some novel biomaterial carriers as the microenvironment for cell culture may also affect cell proliferation and differentiation. For example, graphene and collagen scaffolds have been proven to promote the differentiation of stem cells into cardiomyocytes. Studies have found that when BMSCs are seeded and cultured on graphene, the expression levels of cardiac-specific markers in these cells are significantly higher than those in cells cultured on coverslips. Moreover, the BMSCs on graphene also show significant expression of matricular proteins related to cardiomyocyte differentiation, including collagen types I, III, and IV, fibronectin, and laminin [189]. When BMSCs are directly cultured on collagen scaffolds, it has been found that these cells can produce more cardiomyocyte-specific proteins and secrete cardiac trophic factors such as BMP4, HGF, VEGF, and bFGF, while exhibiting fewer fibrotic characteristics [190]. This highlights the importance of ECM in the differentiation process of stem cells. As the microenvironment for cell interaction, ECM not only provides structural support for damaged myocardium but also enhances the differentiation potential of stem cells. In addition, cell membrane proteins (such as caveolin-1), ion channel proteins (such as vanilloid receptor 1 [VR-1]), and epigenetic modification molecules (such as histone deacetylase 1 [HDAC1]) have also been proven to promote the differentiation of stem cells into cardiomyocytes. Studies have found that treating BMSCs with siRNA for caveolin-1 can enhance the expression of cardiomyocyte markers during BMSCs differentiation by regulating the activation of the STAT3 signaling pathway [191]. VR-1 is a Ca^2+^ channel, and its ligand capsaicin, as a transient receptor potential vanilloid 1 (TRPV1) agonist, has been proven to promote the maturation of mouse embryonic stem cell-derived cardiomyocytes (mESC-CMs) [192]. In BMSCs, knockdown of *VR-1* results in significantly lower mRNA and protein expression of α-MHC, α-actin, and Nkx2.5 compared to the control group, and the study suggests that this may be mediated through the Wnt/β-catenin signaling pathway [193]. Epigenetic modifications play an important role in cell differentiation. Studies have found that treating BMSCs with *HDAC1*-RNAi lentiviral vectors results in significantly higher expression of cardiomyocyte-specific genes in these cells compared to the control group. Treating BMSCs with non-specific HDAC inhibitors (such as valproic acid and trichostatin A) can also achieve the same effect [194]. In addition, recent studies have also focused on nuclear reprogramming. Studies have found that inducing hMSCs with neonatal rat cardiomyocyte lysates (containing cytoplasmic and nuclear components) can promote the expression of cardiomyocyte-specific proteins [195], thereby regulating the gene expression patterns within stem cells. This reveals the mechanisms by which stem cells differentiate into CLCs and provides a direction for promoting their transdifferentiation.

#### CLCs Derived from Umbilical Cord MSCs

Mesenchymal cells isolated from the umbilical cord have been proven to differentiate into all three germ layers and ultimately into CLCs [196]. Among them, amniotic fluid mesenchymal stem cells (AF-MSCs) and mesenchymal stem cells from Wharton’s Jelly (WJ-MSCs) have shown extremely high differentiation potential in vitro. Under the influence of 5-aza, both can exhibit increased expression of GATA4, cTnT, α-actin, and Cx43 [197].

#### CLCs Derived from ADSCs

Various types of ADSCs have been explored for their potential to differentiate into cardiomyocytes, including those derived from white adipose tissue (WAT), brown adipose tissue (BAT), epicardial adipocytes, pericardial adipocytes, and omental adipocytes. Research has indicated that BAT holds greater potential as a source of cardiac cells compared to WAT. Gene expression analysis has revealed that BAT significantly expresses a variety of cardiac-specific genes, including Nkx2.5, GATA4, myosin light chain 2a (MLC2a), MLC2v, cTnT, as well as VEGF and IGF-1. Additionally, it has been found that treating WAT with VEGF and IGF-1 can increase the expression of cardiac-specific genes [198]. Epicardial adipose tissue (EAT) has been found to possess BAT signature genes, such as high expression of uncoupling protein 1 (UCP-1), and is believed to have similar protective effects on the heart [199]. However, studies have shown that compared to induction with 5-aza, transfecting EAT-derived stem cells with retroviral vectors carrying transcription factors (such as estrogen-related receptor gamma [ESRRG], GATA4, myocyte enhancer factor 2 C [MEF2 C], myocardin [MYOCD], TBX5, and zinc finger protein multitype 2 [ZFPM2]) can significantly induce differentiation into cardiomyocytes and upregulate the expression of Actn2 and cTnT [200].

#### CLCs Derived from ESCs

ESCs possess pluripotency and self-renewal capabilities, enabling them to differentiate into all three germ layers (ectoderm, mesoderm, and endoderm). The determination of their cell fate is regulated by a variety of signaling pathways and transcription factors. Studies have shown that miR-1 and miR-133 are significantly enriched in the mesoderm of differentiating mouse embryonic stem cells (mESs), but they exhibit opposite effects when further differentiating into cardiomyocytes or skeletal muscle cells: miR-1 promotes cardiomyocyte differentiation, while miR-133 inhibits this process. Further experiments have demonstrated that miR-1 promotes cardiomyocyte differentiation by inhibiting Dll-1 expression and blocking the Notch signaling pathway in both mouse and human ESCs [201]. Additionally, supplementing IGF-1 in mES suspensions has been shown to enhance the efficiency of cell differentiation into host organ-specific cells after implantation into acute ischemic myocardial injury areas, promoting the expression of α-sarcomeric actin and significantly increasing ventricular wall thickness [181]. BMP2 has been proven to promote ESCs differentiation into cardiomyocytes downstream of HDAC1 and can restore the cells’ calcium transient capability and responsiveness to adrenergic stimulation [202]. The differentiation of ESCs into cardiomyocytes requires the participation of serum-containing medium. Adding fetal bovine serum (FBS) during the hanging drop stage and the first 24 h of adherent culture enabled ESCs treated with BMP4 to achieve cardiomyocyte differentiation. The differentiated cells exhibited expression of cardiac-specific genes, chronotropic responses to active drugs, and numerous intercellular connections, although the myofibrils mainly presented an immature phenotype [203].

#### Maturation of CLCs Derived from Stem Cells

The maturation of cardiomyocytes is a complex process involving the transition of their structural, functional, and electrophysiological characteristics to a mature state. While significant progress has been made in the differentiation of stem cells into cardiomyocytes to promote cardiac tissue regeneration, how to further induce these cells to mature into functional cardiomyocytes and effectively integrate them into normal cardiac tissue remains one of the key challenges in this field. Studies have shown that recapitulating the natural cellular microenvironment and allowing implanted cells to follow the physiological processes of cardiac development are crucial directions for achieving this goal. Embryonic developmental biology research has revealed that during embryonic development, cardiomyocytes are subject to comprehensive regulation by a variety of environmental factors, including stimulation by surrounding metabolites, mechanical forces, ECM remodeling, and electrical stimulation [204, 205]. Additionally, the morphological characteristics of cardiomyocytes in different planes are also important for their maturation [206].

Electrical stimulation, which is closely related to the contractility of cardiomyocytes, has been relatively well-developed in promoting cardiomyocyte maturation. The contraction of cardiomyocytes is triggered by the influx of ions through voltage-gated channels. Under electrical stimulation, the resting membrane potential of immature cardiomyocytes is reduced, and spontaneous contractions can occur [207]. For example, researchers stimulated hiPSC-CMs at a frequency of 1 Hz for 5 days, then increased the frequency by 1 Hz daily until it reached 6 Hz. After a week of stepwise increasing stimulation, the frequency was reset to 1 Hz and continued until day 28. The results showed that this stepwise increasing pacing frequency significantly induced the expression of Cx43, led to more orderly sarcomeric structures within the cells, and resulted in a mature cell phenotype [208]. Further studies compared low-frequency stepwise increase (frequency increased from 1 Hz to 3 Hz daily) with high-frequency stepwise increase (frequency increased from 1 Hz to 6 Hz), and found that the high-frequency regimen significantly enhanced the structural and electrophysiological functions of the regenerated myocardial tissue [209].

Mechanical forces have a direct impact on the maturation of the cardiac conduction system. Current research attempts to reconstruct the mechanical stimulation environment in vitro by simulating cyclic stretching and mimicking the stiffness of the myocardial tissue substrate. On the one hand, using hydrogels and bioreactor systems to apply cyclic stretching and pulsatile flow, it has been found that this stimulation can significantly upregulate the expression of cardiac-related genes (such as β-MHC, cardiac troponin, etc.) in PSC-CMs [210]. On the other hand, culturing cells derived from cardiac spheroids on polyacrylamide gels with a stiffness gradient (8–21 kPa), the results showed that cells gradually exhibited well-aligned myofibrils and longitudinal calcium ion propagation [211], which reflects the maturation and functional optimization of the cells.

During development, the energy metabolism of cardiomyocytes shifts from glycolysis to oxidative phosphorylation, producing ATP that is crucial for cardiac contractile function, which is an important sign of metabolic maturation in cardiomyocytes [212]. One method to promote metabolic maturation is to culture stem cells in a medium that mimics the metabolic environment of the adult heart, inducing cells to shift towards oxidative metabolism. For example, it has been found that when iPSC-CMs are placed in a specific medium supplemented with glucose, calcium, albumin-bound fatty acids, etc., contractile cardiomyocytes with spontaneous beating ability appear [204]. In addition, the expression of ion-specific genes (such as sodium voltage-gated channel alpha subunit 5 [SCN5 A], potassium inwardly-rectifying channel subfamily J member 4 [KCNJ4], and ryanodine receptor 2 [RYR2]) and contraction-related proteins (such as troponin, myosin light chain, myosin heavy chain [MYH], etc.) also significantly increased [213]. Another method is to activate the AMPK pathway, a key protein in energy metabolism, by upregulating genes related to mitochondrial biogenesis and fatty acid oxidation, thereby promoting the metabolic maturation of cardiomyocytes. For example, small molecules such as thyroid hormones and glucocorticoids, as well as regulating hypoxic conditions to trigger adaptive mechanisms in cardiomyocytes, can all promote their metabolic maturation [214, 215].

Cardiac tissue relies on its unique 3D structure and intercellular gap junctions to effectively transmit contractile impulse signals. In vitro simulated 3D cardiac structures aim to support the maturation of cardiomyocyte metabolism and function by mimicking the in vivo physiological microenvironment. Typically, this model is composed of a 3D scaffold seeded with various cell types, including cardiomyocytes, fibroblasts, and endothelial cells. These cellular components collaborate within the 3D scaffold to perform key functions such as biosynthesis, tissue structure formation, and nutritional support, thereby promoting the maturation and functional integration of cardiomyocytes [216]. Studies have found that compared with 2D differentiation, the expression of mature cardiac gene markers (such as gap junction protein alpha 5 [GJA5]/Cx40, potassium voltage-gated channel subfamily A member 5 [KCNA5], and calcium voltage-gated channel subunit alpha 1G [CACNA1G]) in 3D cell aggregates significantly increased, and ATP production increased by 10% [217]. In addition, some studies have inserted microfluidic devices to precisely control the cellular microenvironment in 3D co-culture systems and found that by controlling the flow of nutrients and oxygen, the microenvironment for cardiomyocyte maturation has been further optimized [218].

“Intercellular differentiation” refers to inducing the differentiation of stem cells by co-culturing with cardiomyocytes. For example, a study differentiated CLCs from HF-hiPSCs reprogrammed from skin fibroblasts of heart failure patients and found that functional integration and synchronized electrical activity occurred when co-cultured with neonatal rat cardiomyocytes, a characteristic that was also verified in rat transplantation experiments [219]. This provides a new direction for the repair of cardiac tissue after MI, but its effectiveness and safety still need to be further proven by mechanistic studies.

#### Arrhythmias Associated with CLCs Derived from Stem Cells

In recent years, significant progress has been made in the field of myocardial regeneration and cardiac remodeling with stem cell therapy. Numerous successful transplantation experiments in small animal models have provided strong support for the application of stem cell therapy in cardiac repair, demonstrating its potential for promoting myocardial regeneration. However, as research has delved deeper, especially in large animal models (particularly non-human primates such as pigs and monkeys), researchers have found that although the formation of new myocardium leads to improved cardiac function, it is also accompanied by frequent occurrences of arrhythmias. These arrhythmias mainly include serious issues such as ventricular premature contractions and ventricular tachycardia. For instance, in one study, researchers transplanted differentiated human ESC-CMs into the hearts of pigs 3 weeks after infarction. The results showed that the contractile function of the cardiac region in this group of animals was significantly enhanced compared to the control group, but monomorphic ventricular tachycardia occurred frequently within 3–4 days after treatment [220]. Similarly, in another study, cynomolgus monkeys with MI that received hiPSC-CMs also exhibited similar arrhythmias [221]. It is speculated that the occurrence of these arrhythmias may be related to the differences in heart size among animals. The larger hearts of large animals lead to more pronounced differences in the volume of transplanted cells and derived myocardial tissue, making it easier to induce electrophysiological abnormalities. In addition, compared with small animals, large animals and humans have slower heart rates, and the transplanted cardiomyocytes may more easily form pacemaker rhythms or reentrant circuits, thereby leading to the occurrence of arrhythmias [222]. It is worth noting that the cardiac structure and function of non-human primates are more similar to those of humans, so the results of these animal models can better reflect the potential problems that may arise after human stem cell transplantation. This also suggests that we need to conduct more targeted research before further advancing human trials to overcome potential arrhythmia complications. In addition, research should delve into the adverse reactions that stem cell therapy may cause and their management strategies, thereby providing a more solid scientific basis for clinical applications.

**Fig. 1:**
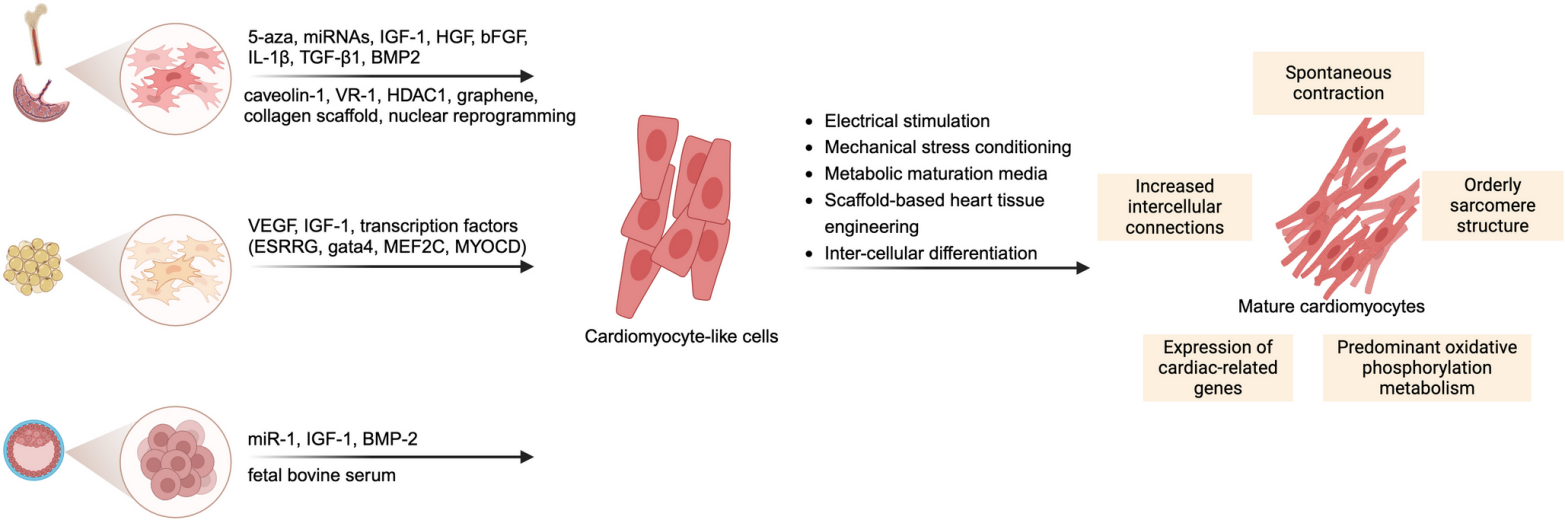
Mechanisms of stem cell therapy in promoting myocardial regeneration. Stem cells from various sources can differentiate into CLCs under the induction of chemical agents, miRNAs, cytokines, and members of the TGF-β superfamily. These CLCs can gradually mature into structurally and functionally mature cardiomyocytes under the combined effects of electrical stimulation, mechanical force induction, and a simulated cardiac physiological microenvironment. 5-aza: 5-azacytidine; IGF-1: insulin-like growth factor 1; HGF: hepatocyte growth factor; bFGF: basic fibroblast growth factor; IL-1β: interleukin-1β; TGF-β1: transforming growth factor-β1; BMP2: bone morphogenetic protein 2; VEGF: vascular endothelial growth factor; ESRRG: estrogen-related receptor gamma; GATA4: GATA binding protein 4; MEF2 C: myocyte enhancer factor; MYOCD: myocardin

Overall, both cell and animal model studies have demonstrated the strong potential of stem cell therapy in post-MI treatment and myocardial regeneration (as shown in Fig. 1). Some initial translational products have also shown positive effects. For example, one study used a cell-scaffold combination composed of human Wharton’s jelly mesenchymal stem cells and a novel composite material (consisting of polyethylene glycol (PEG), hyaluronic acid, and chitosan) and injected it into the infarcted myocardium of rabbits. The results showed that this combination could significantly improve cardiac function and promote angiogenesis and myocardial regeneration [222]. However, despite these encouraging initial findings, rigorous clinical trials and large-scale population cohort studies are still needed to comprehensively validate the safety and efficacy of stem cell therapy before it can be widely applied in clinical settings.

#### Interaction between Stem Cell Therapy and the Post-Infarction Inflammatory Microenvironment

The inflammatory microenvironment following MI significantly impacts the efficacy of cell therapy. Studies have shown that the post-MI inflammatory microenvironment may exert inhibitory effects on implanted stem cells. Therefore, an in-depth analysis of the interaction between the post-MI inflammatory microenvironment and implanted stem cells is crucial for optimizing stem cell therapy. As previously described, the inflammatory phase following MI involves nearly all components of the cardiac microenvironment, including cardiomyocytes, fibroblasts, ECM, endothelial cells, and the immune system. The production of pro-inflammatory cytokines and the infiltration and differentiation of inflammatory cells are the main characteristics of this phase.

### The Inflammatory Microenvironment may be Detrimental To Implanted Stem Cells

#### Effects of Inflammatory Cells and Cytokines

Research has found that the survival rate of BMSCs in ischemic areas significantly decreases in the early post-transplantation period [223]. This phenomenon is closely related to the inflammatory cells and cytokines within the inflammatory environment. For example, cytokines such as TNF-α, the IL-1 family, IL-2, IL-6, IFN-Ɣ, and their associated signaling pathways are closely related to the survival and function of stem cells. Natural killer cells (NK cells), which are immune cells with potent cytotoxicity, play a key role in the rejection of implanted cells or tissues. Studies have shown that upregulation of major histocompatibility antigens (MHC), especially class I and II, in implanted stem cells and/or differentiated cardiomyocytes can lead to rejection of transplanted cells through the cytotoxic response of NK cells [224]. In addition, some cytokine receptors and their ligands, such as TNF-R1, IL-1R, apoptosis antigen 1 (APO-1)/FAS, and TNF-related apoptosis-inducing ligand receptor (TRAIL-R), can induce apoptosis and tissue necrosis by activating the NF-κB cascade [225]. NF-κB is the primary responder to cytokines and other harmful factors such as ROS. Studies have shown that NF-κB, once activated by “death ligands,” can directly induce apoptosis of implanted stem cells through overexpression of the pro-apoptotic Bcl-2-associated X protein (Bax) gene [226]. These “death ligands” can also mediate the autophagy cascade during cardiac injury by inhibiting the mammalian target of rapamycin (mTOR) and Ras pathways [227]. In MSCs, the TNF-α and IFN-Ɣ network can synergistically enhance cell autophagy and apoptosis by stimulating the ROS/ERK1/3 pathway, inducing expression of the BH-3 domain protein Beclin-1, and inhibiting expression of the anti-apoptotic B-cell lymphoma 2 (Bcl-2) [227]. Moreover, TNF-α and its signaling pathways can inhibit the differentiation of CSPCs into cardiomyocytes and promote their development into catecholaminergic-like cells. The mechanism may involve stimulation of the NF-κB and MAPK pathways around the infarct area by TNF-R1 and TNF-R2, thereby reducing the differentiation and proliferation potential of CSPCs [228].

#### Hypoxia and Lack of Growth Factors

Hypoxia-mediated oxidative stress plays a significant role in inducing apoptosis of stem cells. Studies have found that hypoxia can significantly reduce the expression of anti-apoptotic proteins such as Bcl-2, while increasing the expression of pro-apoptotic proteins (such as Bax, Bcl-2-associated death promoter (Bad), and glycogen synthase kinase 3β (GSK-3β)) [229]. In addition, ROS-mediated specific protein misfolding in the endoplasmic reticulum and stimulation of the Ras and ERK1/2 signaling pathways can induce autophagic cell death [230, 231]. It has also been found that TNF-α can inhibit the secretion of stem cell factor (SCF), which is an important factor for inducing cardiac cell differentiation [232].

#### Immunomodulatory Effects of Stem Cells on the Inflammatory Microenvironment

The immunomodulatory effects of stem cell therapy within the post-MI inflammatory microenvironment have become a research hotspot. Stem cells primarily exert immunomodulation through paracrine effects and direct cell-to-cell contact, regulating the survival, proliferation, migration, and phenotypic transformation of host immune cells by secreting various immunosuppressive soluble factors, cytokines, and chemokines.

#### Activation of Stem Cells by the Inflammatory Microenvironment

MSCs are generally considered to possess immune evasion characteristics, and the inflammatory microenvironment can significantly activate the immunomodulatory effects of MSCs [233]. Studies have shown that high concentrations of pro-inflammatory cytokines, such as INF-Ɣ, TNF-α, IL-1α, and IL-1β, can act synergistically to activate MSCs and polarize them towards an immunosuppressive phenotype [234]. Additionally, MSCs can be induced to undergo apoptosis by cytotoxic cells in a perforin-dependent manner, thereby initiating the immunosuppressive effects of MSCs [235]. This suggests that pre-activation of MSCs in vitro can optimize their immunomodulatory effects following therapeutic infusion.

#### Stem Cell-Mediated Recruitment and Infiltration of Immune Cells

Regarding the interaction between MSCs and neutrophils, research has mainly focused on regulating the survival and degranulation processes of neutrophils. In vitro studies have found that co-culture of MSCs and neutrophils can significantly prolong the lifespan of neutrophils, with IL-6 derived from MSCs being a key factor. IL-6 activates the STAT3 transcription factor, thereby extending the lifespan of neutrophils [236]. However, the specific mechanism of prolonging neutrophil lifespan in post-MI repair is not yet clear, as timely inhibition of the inflammatory response to promote fibrotic repair is a necessary process in post-MI repair. MSCs can also mediate the migration and adhesion of neutrophils. For example, MSCs can promote the expression of KYNA (a GPR35 ligand), which facilitates the adhesion of neutrophils to cells expressing ICAM-1 (such as endothelial and cardiomyocytes) [237]. Moreover, TGF-β1 expressed by MSCs has a strong chemotactic effect on neutrophils and monocytes and has been shown to inhibit the cytotoxicity of neutrophils [238].

#### Stem Cell-Mediated Immune Cell Functional Changes and Inhibition of Inflammatory Signals

Studies have found that the release of neutrophil granules is significantly reduced in the presence of MSCs, which is considered an important action of MSCs against the inflammatory microenvironment. A key factor affecting the degranulation response of neutrophils is indoleamine 2,3-dioxygenase (IDO). IDO can inhibit the release of human neutrophil peptide 1–3 (HNP1-3, also known as α-defensin) [239]. α-defensins are a group of pro-inflammatory proteins stored in neutrophil granules that can induce the expression of adhesion molecules and co-stimulatory molecules, promote oxidative stress responses, and inhibit fibrinolysis and block angiogenesis [240]. Additionally, MSCs can inhibit both basal and N-Formyl-L-Methionyl-L-Leucyl-L-Phenylalanine (f-MLP)-induced respiratory burst, thereby reducing neutrophil damage to the myocardium [241]. When MSCs are pre-treated by activating TLR3 and TLR4, co-cultured neutrophils exhibit enhanced survival and bactericidal capacity in a simulated bacterial infection environment [236]. However, whether this phenomenon plays a role post-MI remains to be further explored.

Macrophages are important immune cells in the inflammatory environment. Studies have found that when MSCs are co-cultured with macrophages, they primarily function by promoting the polarization of macrophages towards the anti-inflammatory M2 phenotype [242], with prostaglandin E2 (PGE2), IDO, and MSC-derived IL-10 being proven to participate in this process and validated in a myocardial infarction mouse model. Sixteen weeks after MSC implantation, significant reductions in left ventricular remodeling and fractional shortening were observed in mice [243]. MSCs can also inhibit macrophages from producing pro-inflammatory cytokines such as IL-1β, IL-6, TNF-α, and IFN-Ɣ, while increasing the secretion of the anti-inflammatory cytokine IL-10, a process mainly attributed to the mediation of PGE2 and TGF-β1 [244, 245]. In contrast, the enhancement of macrophage phagocytic activity by MSCs is less pronounced. Additionally, in the context of sepsis, the interaction between MSCs and macrophages involves two negative feedback regulatory loops. For example, TNF-α and other pro-inflammatory cytokines produced by resident macrophages can activate MSCs to secrete the anti-inflammatory mediator TNF-α stimulated gene/protein 6 (TSG-6), which in turn can sequester the NF-κB signaling pathway in macrophages and inhibit the secretion of TNF-α by macrophages [244]. Lipopolysaccharide, TNF-α, NO, and DAMPs from damaged tissues and macrophages can stimulate MSCs to secrete PGE2, which can convert macrophages into a phenotype that secretes the anti-inflammatory cytokine IL-10 [242].

In addition to the aforementioned cell types, most studies on the immunomodulatory capabilities of MSCs have focused on their interactions with T lymphocytes, likely due to the key role of T lymphocytes in graft-versus-host disease. Splenocytes were isolated from rats with MI and injected into healthy control rats after activation. Six weeks later, lymphocyte-mediated myocardial damage was observed in the recipient rats. In vitro experiments indicated that cytotoxic T cells were primarily responsible, causing significant damage to cardiomyocytes. However, the formation of these autoreactive lymphocytes was not immediate; when isolated 3 weeks post-MI, their cytotoxic effects were most pronounced [246]. In addition to cytotoxic T cells, an increase in the Th1 cell subset post-MI has been observed, which has been shown to activate cytotoxic T cells and produce high levels of pro-inflammatory cytokines, thereby suppressing the production of protective Tregs and Th2 cell subsets [247]. Tregs are known to inhibit immune system activation and maintain tolerance to self-antigens, while Th2 cells are involved in humoral immunity, producing immunosuppressive cytokines such as IL-10 and IL-4 [248]. This antigen-antibody reaction against healthy cardiomyocytes may influence long-term myocardial remodeling post-MI. Regarding the mechanisms by which MSCs inhibit T cells, studies have shown that IDO, PGE2, TGF-β1, and IL-10 are likely key players. For example, tryptophan depletion induced by IDO and the accumulation of metabolites (such as kynurenic acid [KYNA] and quinolinic acid) [237], as well as the mediation of PGE2, are considered key pathways for MSCs to inhibit T cell proliferation and promote Tregs formation [249]. MSCs can release TGF-β1 from the TGF-β1/glycoprotein-A repetitions predominant (GARP) complex to inhibit T cell activation [250]; they can also arrest T cells in the G0 phase to control T cell numbers [251]. MSCs can inhibit the nuclear translocation of NF-κB and negatively regulate the co-stimulatory molecule B7-H4 to block cell cycle progression [252]. Additionally, MSCs can block the proliferation and cytotoxicity of NK cells through cytokines such as IFN-Ɣ, TGF-β1, and PGE2 [253]. Meanwhile, IL-10 and TGF-β1 produced by MSCs promote the formation of Th2 cells and induce the formation of Tregs. It has also been found that IL-4 produced by Th2 cells can promote the formation of M2-type anti-inflammatory macrophages.

The contributions of B cells, dendritic cells, and NK cells to the immune microenvironment post-MI are relatively less studied. The mechanism by which MSCs act on B cells primarily involves blocking cell cycle progression and regulating cell differentiation to control the harmful effects of B cells [254]. Additionally, MSCs can affect the chemotaxis of B cells towards CXCL12 and CXCL13, thereby blocking their migration to inflammatory areas [255]. For dendritic cells, it has been found that MSCs can significantly block their differentiation [169] and induce an immunotolerant phenotype through galectin-1-mediated MAPK signaling pathway stimulation [172]. PGE2 is also considered a key mediator for MSCs to target the function and maturation of dendritic cells. Studies have shown that when dendritic cells are co-cultured with MSCs, MSCs can reduce the homing of dendritic cells to lymphoid organs by downregulating CCR7 and integrin α4β1 (CD49 dβ1), thereby inhibiting T cell activation [170]. Moreover, MSCs have been found to inhibit the proliferation of resting NK cells, as well as the production of IFN-Ɣ and cytotoxicity, primarily by blocking activating receptors on the NK cell surface, such as natural cytotoxicity receptor 3 (NKp30), natural killer group 2 member D (NKG2D), and NKp44 [171].

#### Modulation of Inflammasomes by Stem Cells

The activation of inflammasomes has been demonstrated to play a role in the post-MI inflammatory environment. Studies have shown that MSCs can significantly inhibit the expression of nucleotide-binding oligomerization domain-containing protein 2 (NOD2), NLRP3, apoptosis-associated speck-like protein (ASC), and caspase-1 (components of the NLRP3 inflammasome) in the left ventricle of mice infected with coxsackievirus B3. This process is primarily mediated by stanniocalcin-1, an antioxidant protein secreted by MSCs, and offers a novel therapeutic strategy for viral myocarditis [256]. However, whether this mechanism operates similarly in the post-MI inflammatory environment remains to be further proven.

**Fig. 2:**
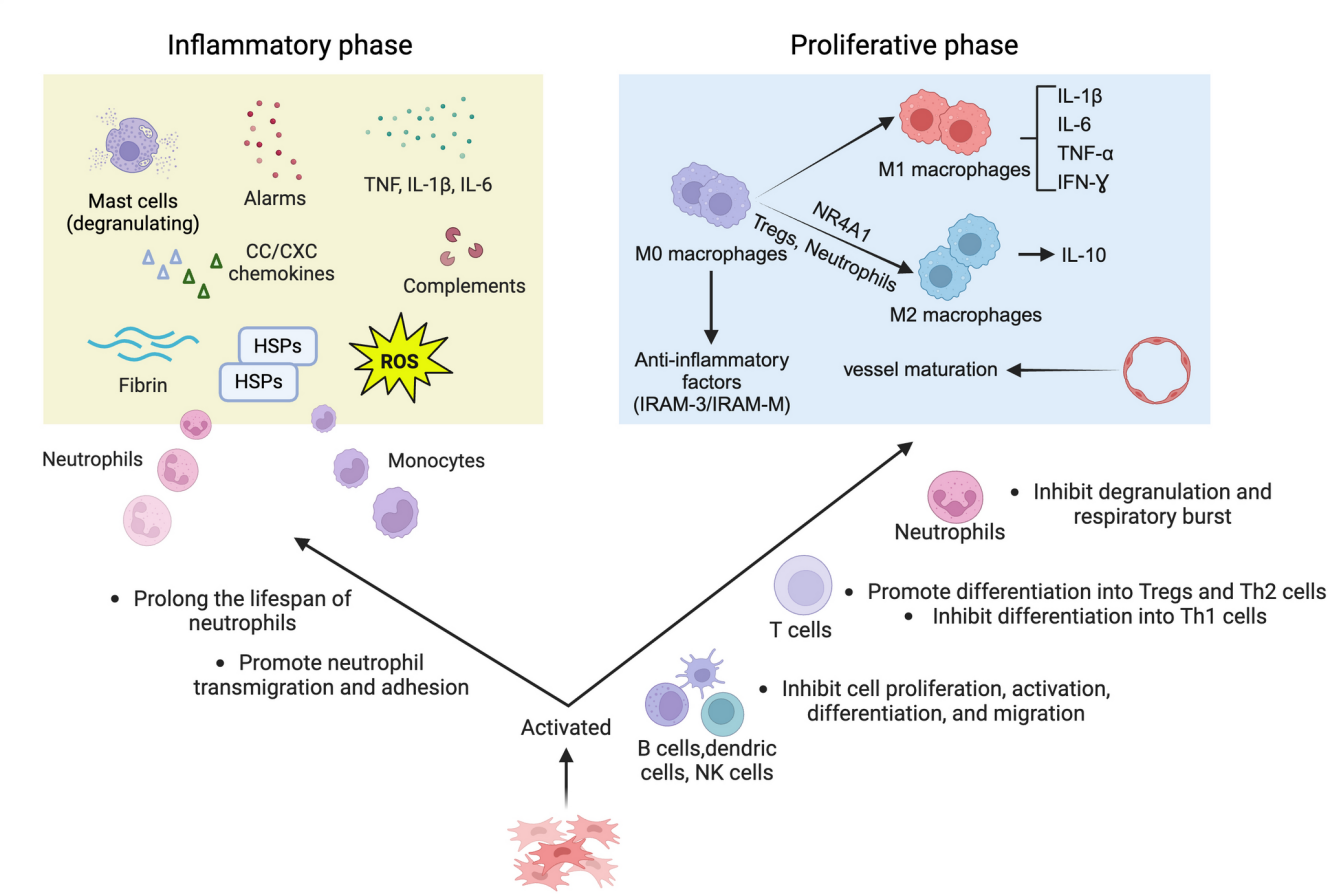
Mechanisms of stem cell therapy in modulating the inflammatory microenvironment after MI. Stem cell therapy plays a crucial role in the inflammatory phase and proliferative phase following MI, modulating the dynamic changes of the inflammatory microenvironment. During the inflammatory phase, cytokines, chemokines, and ROS released by damaged myocardial tissue induce the chemotaxis and infiltration of leukocytes. Stem cells can be activated to promote the recruitment and infiltration of immune cells. In the fibrotic scar phase, stem cells primarily modulate the phenotypic transformation and functional changes of immune cells such as macrophages, neutrophils, and T cells, thereby inhibiting excessive inflammatory responses while promoting angiogenesis and vascular maturation. TNF: tumor necrosis factor; HSPs: heat shock proteins; IFN-Ɣ:Interferon-gamma; NR4 A1: nuclear receptor subfamily 4 group A member 1

Above all, stem cells exert fine-tuned regulation of the inflammatory microenvironment through a complex and diverse array of mechanisms that span multiple levels, from the cellular to the molecular (as shown in Fig. 2). First, stem cells can be activated by the inflammatory microenvironment, polarizing them towards an immunosuppressive phenotype and thereby enabling their immunomodulatory functions. Second, stem cells promote the recruitment and infiltration of immune cells to the site of inflammation by secreting a variety of chemokines and cytokines, thereby establishing an effective immunomodulatory microenvironment at the site of inflammation. Furthermore, stem cells induce functional changes in immune cells, inhibiting the signaling of pro-inflammatory cells, reducing the release of inflammatory cytokines, and promoting the formation of anti-inflammatory phenotypes. Additionally, stem cells can modulate the activation of inflammasomes, thereby reducing the intensity and duration of the inflammatory response. These mechanisms provide a solid theoretical foundation for optimizing the application of stem cell therapy in post-MI cardiac repair. However, despite existing research that has uncovered a variety of immunomodulatory functions of stem cells within the inflammatory microenvironment, the specific details of how these mechanisms operate in the post-MI context remain unclear and require further elucidation.

#### Interaction between Stem Cell Therapy and Post-Infarction Fibrosis

Despite the new hope that stem cell therapy has brought to post-MI cardiac repair, poor cell implantation outcomes remain a major challenge limiting its clinical application. This section will focus on the impact of the host fibrotic environment on stem cell implantation, discuss new findings on the potential interactions between host myocardial fibroblasts, ECM, and implanted cardiomyocytes, and explore the possible feedback loop between cardiac fibrosis and cell implantation.

#### Effects of Tissue Fibrosis on Implanted Cells

As previously described, fibroblasts and ECM dynamics play a key role in myocardial remodeling during the healing process following MI and also significantly affect the fate of transplanted cells. Current understanding of the impact of fibrotic myocardial tissue post-MI on transplanted stem cells is largely based on speculative and descriptive studies of post-MI tissue composition changes, mainly reflected in the following aspects: early inflammatory responses induce an inflammatory cell phenotype in transplanted cells, compromising their survival [257]; activation of myofibroblasts interferes with the electrical coupling of transplanted cells; a stiff matrix hinders cell migration and contractile function; scar tissue alters cardiomyocyte contractility through mechanical signaling, and supramolecular fiber structures impair tissue vascularization. These factors collectively limit the integration and functional performance of transplanted cells in fibrotic myocardium.

#### Activation of Myofibroblasts Interferes with Electrical Coupling of Transplanted Cells

Myofibroblasts are the primary cell type during the fibroproliferative phase following MI, possessing characteristics of both fibroblasts and smooth muscle cells. However, unlike the electrically stimulated contraction of cardiomyocytes (which maintains cardiac rhythmic beating), the contraction of myofibroblasts primarily relies on mechanical forces for tissue repair and remodeling. There are significant differences between the two in terms of molecular markers, tissue environment, and regulatory mechanisms [26]. This difference leads to the contractile forces generated by myofibroblasts being transmitted to the cardiomyocyte membrane and activating mechanosensitive channels, which may subsequently impair the electrophysiological function of cardiomyocytes. Moreover, the α-SMA microfilaments expressed in myofibroblasts can promote the generation of abnormal automaticity and impulse conduction, affecting the electrophysiological properties of cardiomyocytes and enhancing the propensity for arrhythmias through gap junctions [258]. It is thus inferred that myofibroblasts may interfere with the electrical coupling between PSC-derived cardiomyocytes transplanted into the infarcted heart. Some studies to date support this hypothesis. For instance, a study using a 3D cardiac tissue slice model derived from hiPSCs to simulate torsade de pointes (TdP)-like tachycardia in vitro showed that when treated with a potassium ion channel (IKr) blocker, the cardiac tissue exhibited rapid arrhythmias with typical TdP features, and these abnormal waveforms were mainly observed in mixtures of cardiomyocytes and non-cardiomyocytes (fibroblasts) [259]. Additionally, it was found that implantation of hMSCs themselves may cause depolarization-induced conduction slowing through heterocellular coupling and directly release paracrine factors that affect repolarization rates, thereby inducing arrhythmias in cultured neonatal rat cardiomyocytes [260]. Notably, there are differences in the distribution and density of myofibroblasts (Mfbs) in the infarcted and peri-infarct regions following MI. At moderate densities (0–30% of Mfbs forming gap junctions), Mfbs can shorten cell action potentials and exacerbate the propensity for arrhythmia formation; whereas at high densities (80% of Mfbs forming gap junctions), Mfbs can counteract arrhythmias by causing resting depolarization and blocking conduction [261]. Moreover, the adverse electrophysiological consequences following cell therapy may be related to the ratio of myofibroblasts to implanted cells in the tissue. For example, compared to small animal models (rodents), non-lethal ventricular arrhythmias were observed in primate models transplanted with hESC-CMs, which may be attributed to spontaneously formed reentrant circuits in the animal’s myocardial tissue [220]. However, current research on the impact of the post-MI fibrotic environment on implanted cells remains very limited. Future studies should also consider exploring the effects of using stem cells from different sources, altering cell doses, or changing implantation methods at different stages post-MI on the efficacy of stem cell therapy, in order to optimize the application of stem cell therapy in the post-MI microenvironment.

#### Stiff Matrix Impedes Cell Migration and Contraction

Following MI, excessive collagen deposition significantly increases the stiffness of myocardial tissue (from 10 kPa in a normal adult heart to over 100 kPa in fibrotic tissue), which may contribute to diastolic and systolic dysfunction post-MI [262]. In vitro studies have shown that high-density collagen (≥ 6 mg/mL) restricts the formation of electrical coupling, cellular maturation, and tissue contractility in hESC-CMs lacking cell-to-cell contact, thereby significantly limiting cardiomyocyte function [263]. Moreover, research encapsulating hiPSC-CMs in chemically crosslinked gelatin hydrogels (1.25 × 10^8^/mL) has found that compared to cells in high-stiffness (16 kPa)/slow-degrading hydrogels, hiPSC-CMs in low-stiffness (2 kPa)/fast-degrading and medium-stiffness (9 kPa)/moderately degradable hydrogels can form significant intercellular networks, significantly express α-actinin and Cx43, and exhibit enhanced contractility [264]. These studies highlight the importance of physiological collagen density and matrix stiffness in the formation of stem cell-derived myocardial tissue.

#### Scar Tissue Alters Cardiomyocyte Contractility Through Mechanical Signaling

As previously mentioned, the ECM affects cardiomyocyte contractile activity through molecular mechanosensors and mechanotransducers such as integrins and the actin cytoskeleton. However, studies have found that force generation or contractility induced by non-muscle myosin also plays an important role in the mechanical sensing of cardiomyocytes. Non-muscle myosin is a motor protein that primarily participates in mechanotransduction by regulating the tension between cells and the matrix. In a MI mouse model, PKC and non-muscle myosin activity are significantly upregulated at the integrin adhesion sites of cardiomyocytes, which allows the increased matrix stiffness due to tissue fibrosis and mechanical tension to be transmitted to cardiomyocytes, leading to abnormal mechanical sensing in cardiomyocytes and thereby promoting cardiac remodeling and even heart failure [265]. As the scaffold for transplanted cell attachment, whether the increase in in vivo tissue stiffness post-MI affects cell penetration, migration, and interaction remains unclear and requires further research to elucidate the impact of scar tissue on stem cell implantation and therapeutic effects.

#### Supramolecular Fiber Structures Impair Tissue Vascularization

Transplanted cardiomyocyte tissue derived from implanted cells requires vascularization to provide adequate nutrition and oxygen supply. However, scar formation in ischemic areas can affect the heart’s angiogenic capacity [266]. The process of blood vessel formation, including the proliferation, migration, and sprouting of vascular cells, may be reshaped by the dynamic changes in the ECM during myocardial fibrosis. Additionally, the excessive accumulation of crosslinked type I collagen copolymers may form a physical barrier that hinders the migration of transplanted cells or restricts the infiltration of host-derived microvessels. Fibrotic tissue also expresses a variety of anti-angiogenic or pro-apoptotic factors, such as Ang-II and TGF-β1, which limit blood vessel formation [267]. These factors collectively significantly impact the vascularization capacity of scar tissue for implanted stem cells post-MI.

#### Antifibrotic Effects of Stem Cells

Myocardial injury leads to the loss of contractile myocardial tissue, which is typically irreversible in the absence of effective regeneration. Cell-based therapies have been shown to repair scarred myocardial tissue and reduce scar size, a conclusion that has been validated in multiple clinical studies.

### Non-Cardiac-Derived Stem Cells

#### BMSCs

Experimental studies have shown that injection of BMSCs near the infarct site significantly reduces cardiac fibrosis. In vitro experiments have demonstrated that the conditioned medium of BMSCs can reduce collagen production by cardiac fibroblasts and decrease their responsiveness to TGF-β stimulation. It has been found that the expression of TGF-β neutralizing proteoglycan biglycan and IGF-1 in the culture medium is significantly increased, with biglycan possessing high antifibrotic activity and IGF-1 having anti-apoptotic effects [268, 269]. Moreover, following the transplantation of BMSCs, the expression of pro-fibrotic molecules (such as miR-21 and miR-155) in the infarcted myocardium is significantly reduced [270].

#### MSCs

The therapeutic potential of MSCs in the damaged heart has been confirmed in numerous studies. Implanted MSCs promote collagen degradation in scar tissue through paracrine effects, such as the expression of cytokines and MMPs. For example, in a rat MI model, following MSCs transplantation, the mRNA and protein expression of type I collagen, type III collagen, TIMP-1/2, and TGF-β1 in the tissue were significantly reduced, while left ventricular systolic pressure and left ventricular systolic and diastolic peak velocities were significantly increased [271, 272]. Additionally, MSCs can inhibit fibroblast activation and proliferation, reduce collagen deposition, and regulate the synthesis of MMPs and their inhibitors. For instance, MSCs can inhibit the activation of cardiac fibroblasts induced by hypoxia, with leptin knockout playing a key role [271]. Some genes that inhibit cell proliferation in the conditioned medium of BMSCs, such as elastin and DNA damage-inducible transcript 3, are upregulated [273]. MSCs can also alleviate myocardial fibrosis by secreting antifibrotic factors (such as adrenomedullin [ADM]), with ADM-overexpressing MSCs showing reduced MMP2 levels and improved cardiac function [274]. Moreover, HGF secreted by MSCs can inhibit the pro-fibrotic effects of miR-155 [270]. Inhibiting nuclear factor (erythroid-derived 2)-like 2 (Nrf2) in MSCs via siRNA can reduce collagen deposition in the infarct area and decrease myocardial cell regeneration post-MI [275]. In addition to their effects on the ECM, MSCs also possess immunomodulatory functions, as evidenced by their interactions with cells involved in innate and adaptive immune responses (as previously described). Since chronic inflammation is an important driving force in the development of cardiac fibrosis, the anti-inflammatory effects of MSCs may be intertwined with their antifibrotic actions.

#### Other Stem Cells

In addition to the aforementioned two major categories of stem cells, other cell types such as ESCs, skeletal muscle fibroblasts (SMBs), smooth muscle cells (SMCs), and pericytes (PCs) have also been found to possess antifibrotic potential. A summary of the antifibrotic effects of these cells is presented in Table 2.

**Table 2:** Antifibrotic effects and mechanisms of non-cardiac-derived stem cells

Cell type	Mechanisms	References
BMSCs	1. Inhibition of the TGF-β signaling pathway, reducing the transdifferentiation of fibroblasts into myofibroblasts. 2. Secretion of antifibrotic factors (e.g., HGF, bFGF, IGF-1).	[267–269]
MSCs	1. Promotion of ECM component degradation (e.g., collagen). 2. Suppression of fibroblast activation and proliferation, reducing collagen synthesis. 3. Secretion of antifibrotic factors (e.g., ADM, HGF, Nrf2). 4. Immune modulation to reduce inflammatory responses.	[270–272]
ESCs	1. Cell-cell interactions: promoting cardiomyocytes survival. 2. Paracrine effects: reducing the transdifferentiation of fibroblasts into myofibroblasts, promoting the transition of M1 macrophages to M2 macrophages	[276, 277]
SMBs	1. Regulation of MMP (e.g., MMP2, MMP9) and TIMP (e.g., TIMP4) activities. 2. promoting ECM degradation and inhibiting synthesis.	[278, 279]
SMCs	1. Inhibition of fibroblast transdifferentiation into myofibroblasts.	[280]
pericytes	1. Inhibition of fibroblast transdifferentiation into myofibroblasts (via secretion of miR-132).	[281]

#### Cardiac-Derived Stem Cells

Endogenous cardiac stem cells (i.e., cardiac progenitor cells, CPCs) have also been shown to improve the fibrotic environment, primarily through the following two mechanisms: (1) promoting the degradation of ECM in myocardial scars; and (2) promoting ventricular wall thickening and tissue vascularization. For example, it has been found that CPCs recruited by local stimulation can secrete collagen-degrading enzymes such as MMP2/9/14, while inhibiting the expression of TIMP-4, thereby reducing the adverse effects of scar tissue [282]. Co-transplantation of CPCs with EPDCs or MSCs can produce synergistic effects, further reducing the size of infarcted tissue. It has also been found that changes in the cellular secretome, such as significant increases in the expression of VEGF-A, placental growth factor (PLGF), and PDGF-BB, contribute to improved cardiac function and reduced left ventricular remodeling [283]. Further exploration of the important signaling pathways involved in these cells will provide new insights into stem cell antifibrosis.

#### Improving the Fibrotic Microenvironment to Enhance the Efficacy of Stem Cell Therapy

Despite encouraging results with stem cell-derived cardiomyocytes in animal studies, the translation of these findings to clinical applications still faces numerous challenges, including low graft survival rates, poor functional integration, and suboptimal therapeutic outcomes. In addition to optimizing the characteristics of the transplanted cells themselves, modulating the target of cell therapy—the fibrotic environment—to enhance functional integration between cells and tissues is equally crucial.

#### Pharmacological Intervention to Modulate the Fibrotic Environment

The combination of stem cell-based regenerative medicine and pharmacological therapy is considered a promising strategy for replacing lost myocardium and restoring cardiac function. Pharmacological interventions aimed at inhibiting or reversing fibrosis and its adverse outcomes have been widely applied in the treatment of heart disease, such as ACE inhibitors and statins [284]. Statins possess antioxidant and anti-inflammatory properties that can reduce leukocyte infiltration in infarcted myocardium, thereby protecting the endothelial barrier and inhibiting the transdifferentiation of cardiac fibroblasts into myofibroblasts [285–287]. Studies have shown that pre-treatment with statins in MI models can reduce cell apoptosis and enhance the migration and survival of implanted stem cells by activating signaling pathways such as SDF-1α/CXCR4 [288, 289]. Moreover, simvastatin treatment has been proven to enhance cardiac-related gene expression and cardiomyogenesis in ESCs [287]. In addition to statins, whether other antifibrotic drugs similarly enhance the effects of stem cell therapy requires further investigation.

#### Modulating Fibrosis-Related Target Genes

Studies have shown that targeting genes involved in the regulation of matricellular protein expression and matrix remodeling may help reduce scar barriers in the vicinity of cell injection, thereby improving the survival and functional integration of implanted cells in infarcted myocardium. Adenylyl cyclase-6 (AC6) is an early and effective target in this direction, which can target the TGF-β pathway, inhibit fibroblast activation and collagen synthesis, and thereby promote cardiac function improvement following cell implantation in mice [290]. In addition, overexpression of miR-29 has been shown to significantly enhance the migration and vascularization of iPSC-derived cells in implanted cell sheets within infarcted myocardium [291], a finding that has been preliminarily validated in a Phase 1 clinical trial (NCT02603224). However, this combination of gene therapy and stem cells still requires further research in terms of appropriate delivery vehicles, tissue targeting specificity, and recipient immune responses.

**Fig. 3:**
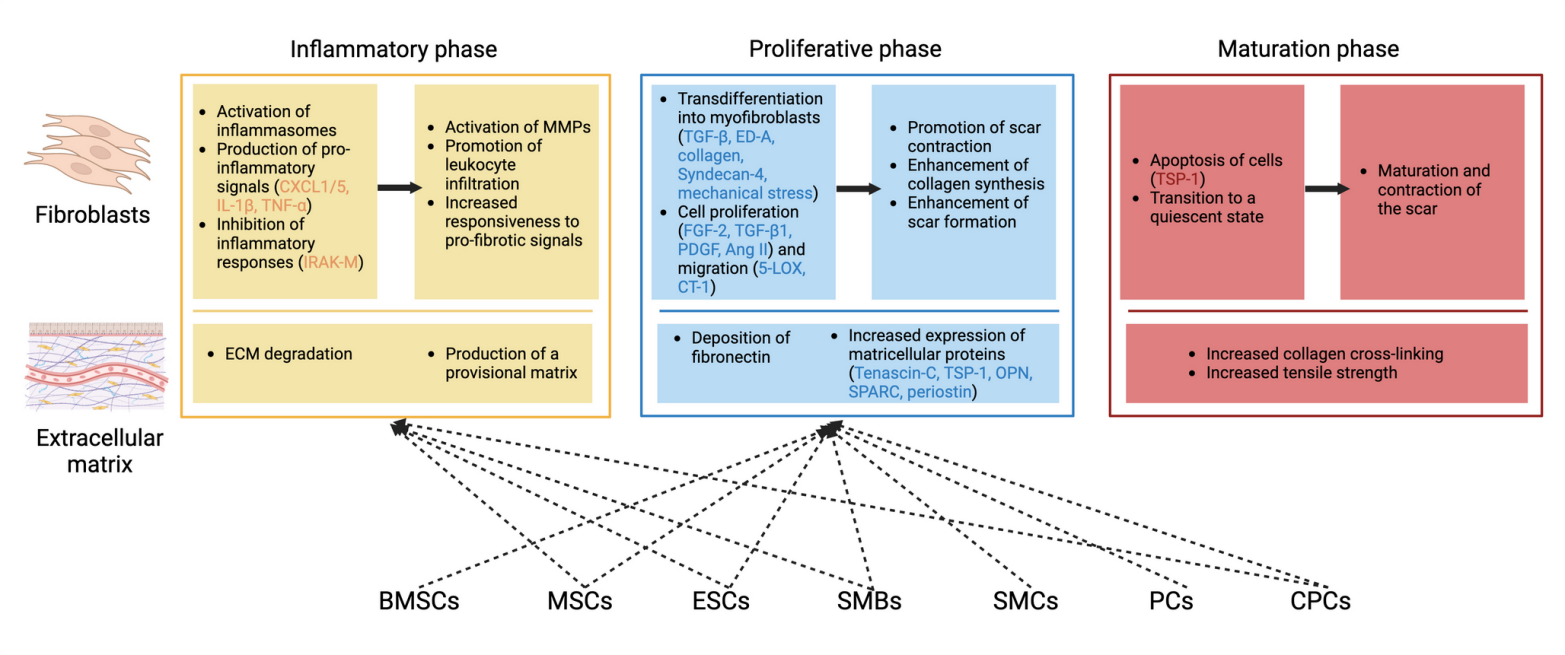
Mechanisms of Stem Cell Therapy in Resisting Tissue Fibrosis After MI. CXCL1/5:keratinocyte-derived chemokine 1/5; IRAK-M: IL-1 receptor associated kinase; FGF-2: fibroblast growth factor 2; PDGF: platelet-derived growth factor; Ang II: angiotensin II; 5-LOX: 5-lipoxygenase; CT-1: cardiotrophin-1; TSP-1: thrombospondin 1; OPN: osteopondin.

In summary, implanted stem cells are believed to reduce cardiac fibrosis, and reducing scar formation will allow enhanced cell migration and implantation, ultimately establishing a positive feedback loop (as shown in Fig. 3) to promote re-myogenesis in the infarcted heart, which is the best expectation. It should be noted that although stem cell therapy has the potential to reduce fibrosis, the adverse effects of fibrotic tissue on implanted stem cells greatly limit the efficacy of cell therapy. Therefore, developing and optimizing stem cell therapy strategies that improve the fibrotic microenvironment is of great significance.

#### Stem Cell Therapy and Angiogenesis

Tissue repair following MI involves a robust angiogenic response, which is crucial for reducing scar formation and improving adverse left ventricular remodeling. During embryonic development, blood vessel formation begins with the assembly of mesoderm-derived endothelial precursor cells, which differentiate to form primitive vascular plexi. Subsequently, endothelial cells sprout to form a microvascular network, which is remodeled to form a complete vascular tree [292]. Post-MI, the formation of a new microvascular network within the tissue is primarily achieved through sprouting angiogenesis from pre-existing endothelial cells in the infarct border zone, with this process involving a gradual extension from the border zone towards the center of the infarct [109]. Stem cells, as therapeutic cells with multidifferentiation potential, show broad application prospects in the treatment of MI-related tissue ischemia. Studies have shown that bone marrow-derived stem cells, pluripotent stem cells, and progenitor cells can promote angiogenesis and restore tissue function, thereby playing an important role in improving cardiac function post-MI.

#### Treatment of MI Using EPCs and MNCs

In the treatment of MI, stem cells mainly participate in angiogenesis to repair tissue ischemia by releasing pro-angiogenic factors and/or differentiating into vascular cell lineages. For example, EPCs and MNCs can differentiate into the endothelial cell lineage, thereby participating in the formation of new blood vessels. EPCs are derived from CD34^+^ hematopoietic stem cells and co-express CD34, CD133, and KDR. Studies have shown that EPCs can be mobilized to the circulation to participate in angiogenesis and promote endothelial repair following tissue injury [293]. During vascular injury, tissues release large amounts of VEGF, which not only mobilizes bone marrow-derived cells to home to ischemic organs but also induces the production of stromal cell-derived factor-1 (SDF-1). SDF-1 can maintain the homing of circulating bone marrow cells and promote angiogenesis [294]. Kamihata et al. transplanted CD34^+^ BM-MNCs into ischemic myocardium in pigs and found increased local blood flow and capillary density, with BM-MNCs being incorporated into over 30% of the newly formed capillaries. Additionally, pro-angiogenic ligands and cytokines, such as IL-1 and TNF-α, were detected in the tissue [295].

#### Treatment of MI Using ESC and PSC-Derived ECs

PSCs mainly promote angiogenesis by differentiating into the endothelial cell lineage and functionally integrating into blood vessels and surrounding tissues. Studies have shown that injection of allogeneic VE-cadherin-positive ESC-ECs into the peri-infarct region of mice significantly increased capillary density in the infarct area [295]. iPSC-ECs have been shown to express endothelial cell markers, including CD31, CD144, VEGFR2 (KDR), and Von Willebrand factor [296], and to possess endothelial cell functions to some extent, such as forming tube-like structures on 3D matrices and chemotactic migration in response to VEGF [297]. In vitro experiments further confirmed that iPSC-ECs can form functionally intact vascular networks together with mesenchymal precursor cells and pericytes [298]. Lee et al. injected differentiated canine iPSC-ECs into a mouse model of MI, and the results showed significant improvement in myocardial contractility in mice, with reporter gene-positive iPSC-ECs still detectable 42 days after administration [299]. Gu et al. generated iPSC-ECs from porcine iPSCs and transplanted them into a mouse model of MI, and mechanistic studies found that iPSC-ECs may promote angiogenesis and cardiomyocyte survival by releasing various pro-angiogenic factors (such as VEGF-1 and angiopoietin) and anti-apoptotic factors, thereby improving cardiac ejection fraction [300].

#### Treatment of MI Using MSCs

MSCs have relatively limited differentiation capacity, and although they can differentiate into the endothelial cell lineage or express endothelial lineage markers, their main role in angiogenesis is achieved through paracrine mechanisms. Studies have shown that after transplantation of a cell sheet composed of MSCs onto the epicardium of infarcted rats, a thick layer structure containing new blood vessels, undifferentiated cells, and a small number of cardiomyocytes was formed at the implantation site. The transplanted cell sheet enhanced angiogenesis through paracrine signaling, reversed ventricular wall thinning, and significantly improved cardiac function [301]. In vitro studies further indicated that the conditioned medium of MSCs can promote capillary lumen formation by increasing sprouting, chemotaxis, and endothelial cell proliferation [302]. The recognized effective soluble cytokines mainly include the VEGF superfamily, bFGF, HGF, IGF-1, and MCP-1. Implantation of MSCs has been shown to lead to increased secretion of VEGF [303], which induces angiogenesis post-AMI by activating the ROS-endoplasmic reticulum stress-autophagy axis in vascular endothelial cells [304]. As a member of the VEGF superfamily, PLGF also plays an important role in MSC-mediated angiogenesis and can recruit more MSCs to ischemic sites through macrophage polarization, forming a positive feedback loop [305]. bFGF mainly acts by enhancing the proliferation of endothelial and smooth muscle cells [306], although validation in tumor cells suggests that the AKT/HIF-1α/VEGF signaling pathway may be activated [307], its specific mechanism of action in ischemic myocardium still requires further exploration. HGF secreted by MSCs has been shown to protect endothelial barrier function by increasing the expression of endothelial cell adhesion molecules through Rac family small GTPase 1 (Rac1) [308]. In animal models of MI, MSCs transfected with HGF were also found to reduce infarct size, increase cell survival, and reduce collagen deposition [309], but the specific mechanisms are not yet clear. IGF-1 is mainly secreted by adipose-derived MSCs [310], and its overexpression has been shown to promote angiogenesis by activating the PI3 K/AKT pathway [311], and improve microcirculation perfusion and LVEF [312]. MCP-1 is a key chemokine that stimulates the migration of vascular smooth muscle cells, endothelial cells, and MSCs [115], and it can mediate the activation of endothelial cell *cadherin-12* and *cadherin-19* gene expression by the transcription factor MAP-1 induced protein (MCPIP) [313].

Similarly, in light of the low survival rate of MSCs following transplantation, researchers have explored various methods to enhance MSC-mediated angiogenesis, including pharmacological preconditioning, coculture or cotransplantation with supportive cells, physical stimulation, tissue engineering, and genetic modification. MSCs preconditioned with atorvastatin (ATV) have been shown to increase the expression of VEGF and intercellular adhesion molecule 1, thereby enhancing endothelial cell function [314]. Moreover, pharmacological treatment can upregulate the expression of C-X-C chemokine receptor 4 on the surface of MSCs, mediating their migration to damaged myocardium through interaction with SDF-1 [315]. The improvement of cell therapy by ATV preconditioning has also entered clinical trial stages. Regarding cytokines, Angiotensin II (Ang II) preconditioning of MSCs has been shown to enhance VEGF expression [316]. Cotreatment of MSCs with IL-1 and TGF-β1 can promote the formation of the ATF2-SP1-SMAD3 transcriptional complex, inducing activation and further synthesis of VEGF at the promoter site [317].

Interactions between supportive cells and MSCs can regulate angiogenesis through paracrine effects and cell-to-cell contact. On one hand, using supportive cells that cannot directly differentiate into endothelial cells can serve as an auxiliary means to enhance the reparative effects of MSCs. For example, coculture with BM-MSCs can promote the shift of macrophage phenotype towards the M2 type, which, through the production of anti-inflammatory factors, synergistically promotes angiogenesis [318]. On the other hand, both MSCs and supportive cells can differentiate into endothelial cells or respectively lead to neovascularization. For instance, MSCs cocultured with BM-EPCs can express vascular markers beyond those expressed in separate cultures, while EPCs can stimulate MSCs proliferation, and in turn, MSCs can enhance EPC survival [319]. Hypoxia preconditioning has been proven to be a powerful stimulus for enhancing the pro-angiogenic effects of MSCs, mainly due to the upregulation of HIF-1α and its target genes (such as VEGF, glucose transporter 1, and lactate dehydrogenase A), as well as the enhanced anti-apoptotic capacity of MSCs [320]. The application of tissue engineering tools such as biohydrogels, adhesive microdroplets, and cell patches supports the retention, survival, and secretion of pro-angiogenic factors of MSCs after transplantation through encapsulation and cross-linking actions [321]. Genetic modification can more directly regulate the paracrine effects of MSCs to maximize their pro-angiogenic effects. In addition to the cytokines mentioned above produced by MSCs, overexpression of anti-apoptotic genes (such as Bcl-2-like protein 1 [BCL2-like protein 1, *BCL-XL*]) [322] and chemokines (such as SDF-1α) [323] has also been shown to promote angiogenesis and has been verified in both in vitro and in vivo experiments.

**Fig. 4:**
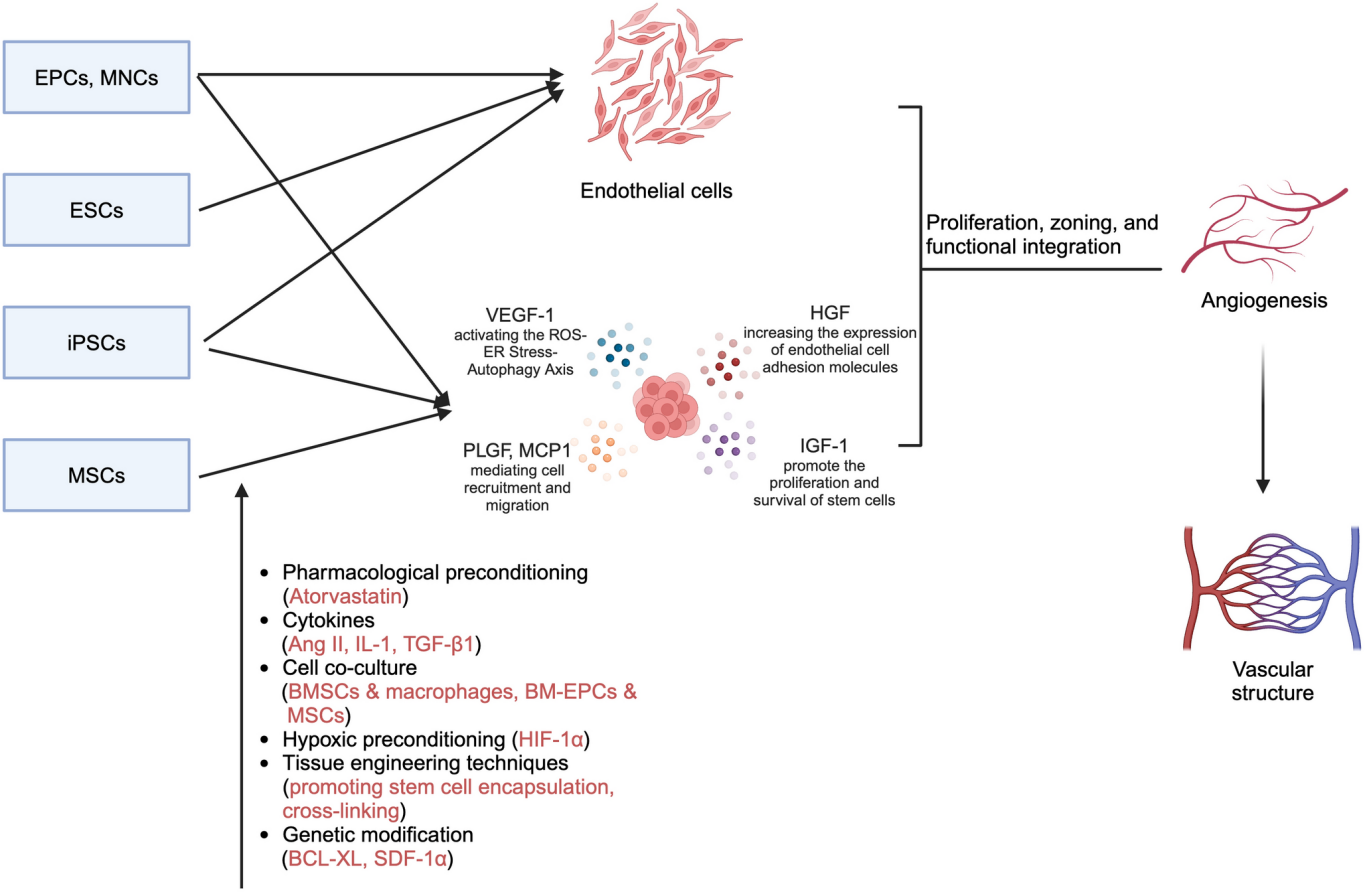
Mechanisms of stem cell therapy in promoting angiogenesis Post-MI. In the treatment of MI, stem cells mainly promote angiogenesis and repair tissue ischemia through two pathways. On one hand, stem cells can differentiate into vascular cell lineages, such as endothelial cells, to directly participate in the construction of new blood vessels. On the other hand, stem cells can secrete a variety of pro-angiogenic factors, such as VEGF, HGF, PLGF, MCP1, and IGF-1, which can work together to stimulate endogenous angiogenesis and improve local blood flow perfusion

These data collectively demonstrate the feasibility and efficacy of improving ischemic myocardium through the transplantation of stem cells and progenitor cells in preclinical animal models. However, current research still has some limitations. For example, the use of immunodeficient animal models or immunosuppressive treatment of transplanted human cells may result in a lack of functional immune response, thus failing to fully simulate the physiological environment within the human body. Moreover, it is difficult to clearly distinguish the specific mechanisms by which stem cells promote myocardial tissue regeneration or improve cardiac function in both in vitro and in vivo studies (as shown in Fig. 4), that is, whether it is achieved through promoting angiogenesis or directly promoting cardiomyocyte regeneration. Despite these limitations, these studies still provide encouraging results. The ability of stem cells to secrete a variety of pro-angiogenic factors and their potential to integrate into the vascular system are the most direct means of promoting angiogenesis.

#### Extracellular Vesicles (EVs) Derived from Stem Cells

Extracellular vesicles (EVs) are nano-sized, membrane-bound vesicles released by cells, capable of transporting bioactive molecules such as proteins, lipids, and nucleic acids. They significantly influence the gene expression and cellular behavior of terget cells, thereby playing a crucial role in cell-to-cell communication [324]. For stem cells such as MSCs, EVs are indispensable in assisting functional components (such as miRNAs and proteins) to cross the cell membrane. An increasing number of studies have proven that the cardioprotective effects of stem cells mainly originate from their paracrine effects, rather than direct cardiomyocyte regeneration. In the context of MI, small extracellular vesicles (sEVs), as a cell-free therapy, are considered a safer alternative to virus-mediated transgenic reprogramming due to their low immunogenicity and minimal risk of immune rejection. In body fluids, EVs are mainly divided into sEVs, apoptotic bodies (ABs), and large extracellular vesicles (lEVs) [324]. Although previous reviews have comprehensively summarized the role of stem cell-derived sEVs in post-MI cardiac repair, most studies have focused on the phenotypic and functional descriptions of target cells/tissues. This section will delve into the specific mechanisms and related signaling pathways by which stem cell-derived sEVs promote angiogenesis, regulate inflammation, resist fibrosis, and induce cardiomyocyte regeneration.

#### Stem Cell-Derived EVs Promote Angiogenesis

In the angiogenesis following MI, studies have shown that sEVs play a key role by transferring pro-angiogenic miRNAs, paracrine factors that promote angiogenesis, and inducing autophagy in endothelial cells, which may involve signaling pathways such as Notch, MAPK, NF-κB, and PI3 K/Akt.

#### Notch Signaling Pathway

Delta-like 4 (DLL4), a membrane-bound ligand of the Notch signaling family, plays a crucial role in angiogenesis. In vitro experiments have shown that sEVs derived from MSCs can promote the formation of tube-like structures in human umbilical vein endothelial cells (HUVECs), while in vivo experiments have shown that they can mobilize endothelial cells into Matrigel plug, thereby promoting angiogenesis. This may be attributed to the secretion of miR-30 family members by MSC-derived sEVs [302]. Further zebrafish embryo experiments have found that miR-30b can inhibit the expression of Dll4 by targeting it, leading to excessive vascular sprouting and accelerated angiogenesis. In addition, loss-of-function experiments have shown that the absence of miR-30 can significantly inhibit the pro-angiogenic ability of MSC-derived sEVs [325]. Studies have found that sEVs derived from MSCs overexpressing HIF-1α (HIF-MSC-Exos) can significantly increase proteins related to the Notch signaling pathway, with the highest content of Jagged1 (a Notch ligand), which is also the only Notch ligand packaged into sEVs. Further in vivo and in vitro experiments have found that inhibiting Jagged1 can block HIF-MSC-Exos-induced angiogenesis [326]. This proves that the activation of the Notch signaling pathway induced by HIF-1α is the main mechanism of its pro-angiogenic effect.

#### MAPK Pathway

VEGF and FGF are two of the most potent pro-angiogenic factors, which promote angiogenesis by activating the MAPK pathway. MiR-126 secreted by MSC-derived sEVs has been shown to regulate this pathway and affect angiogenesis. Studies have found that overexpression of miR-126 can significantly enhance FGF-2-induced ERK1/2 (MAPK) phosphorylation, while knockdown of miR-126 has the opposite effect [327]. Huang et al. have proven that preconditioning with ATV can similarly enhance the positive effect on this pathway. Their study found that the expression of lncRNA-H19 in MSC-ATV-Exos was significantly increased, and the miR-675-3p and miR-675-5p encoded by the latter were also increased in co-cultured endothelial cells [314]. Under hypoxic conditions, overexpression of miR-675-5p can further enhance the level of HIF-1α, which, as the main transcription factor of VEGF, promotes the expression of VEGF and, in synergy with ICAM-1, promotes angiogenesis, showing advantages in improving cardiac function and reducing infarct size in a rat model of MI [328]. In addition, increased activity of Ras protein can also activate the expression of downstream MAPK (such as ERK1/2), thereby promoting endothelial cell migration and tube formation. Studies have found that miR-132 secreted by MSC-derived sEVs can activate the Ras-MAPK pathway by inhibiting the expression of RASA1 (a Ras GTPase-activating protein), thereby promoting angiogenesis in a mouse model of myocardial infarction [329].

#### NF-κB Signaling Pathway

By simulating peripheral artery disease (an ischemia-related condition) and conducting proteomics analysis, studies have identified 1,937 proteins in sEVs derived from MSCs, recognizing pro-angiogenic molecules such as PDGF, epithelial growth factor (EGF), FGF, and NF-κB. This indicates that sEVs derived from MSCs can also secrete potential angiogenic paracrine factors. Among them, in vitro experiments have shown that the NF-κB inhibitor (pyrrolidine dithiocarbamate, PDTC) can significantly inhibit the tube formation capacity of sEVs, indicating that the NF-κB signaling pathway is a key mediator of MSC sEVs-induced angiogenesis [330]. It is thus inferred that sEVs derived from MSCs post-MI may exert similar effects, although the molecules affected in the pathway still require further investigation.

#### PI3 K/Akt Signaling Pathway

The PI3 K/Akt signaling pathway is primarily involved in cell survival, proliferation, metabolism, and angiogenesis. The activation of the downstream protein eNOS can increase NO production, thereby promoting vasodilation and angiogenesis. Studies have found that sEVs derived from MSCs overexpressing miR-126 can significantly enhance the levels of p-Akt/Akt and p-eNOS, and promote the proliferation, migration, and tube formation capacity of endothelial cells damaged by hypoxia/reoxygenation (H/R), while the PI3 K inhibitor (LY294002) can inhibit these effects [331]. This indicates that the PI3 K/Akt/eNOS signaling pathway is involved in the protective effects of MSC-EXs on H/R-damaged endothelial cells. Phosphatase and tensin homolog (PTEN) is a negative regulator of the PI3 K/Akt signaling pathway, which inhibits Akt activation by dephosphorylating PIP3 and reducing its levels. Compared to BM-MSCs and ADMSCs, sEVs secreted by endometrial mesenchymal stem cells (EnMSCs) highly express miR-21, which has been proven to target PTEN to activate the Akt signaling pathway, and increased expression of VEGF has been observed in co-cultured endothelial cells, thereby promoting angiogenesis. Additionally, increased expression of the anti-apoptotic protein Bcl-2 and decreased expression of apoptosis-related proteins Bax and caspase-3 have been observed in co-cultured cardiomyocytes [332], demonstrating the anti-apoptotic and pro-angiogenic effects of the miR-21/PTEN/Akt axis in infarcted myocardium.

In addition to the aforementioned signaling pathways, studies have found that sEVs derived from MSCs may also enhance angiogenesis by promoting autophagy in endothelial cells. It has been observed that a large number of MSCs undergo apoptosis shortly after transplantation, which seems to contradict the therapeutic effects ultimately produced. Further analysis revealed that ABs released during MSCs apoptosis may play an important role. It was observed that ABs released by MSCs were phagocytosed by endothelial cells, activating their lysosomal function and promoting the expression of transcription factor EB (TFEB), thereby significantly enhancing autophagy. Importantly, ABs also upregulated pro-angiogenic genes (such as ANGPT1 and KDR) in endothelial cells, while downregulating anti-angiogenic genes (such as THBS1 and vascular endothelial specific angiogenesis inhibitor 1 [VASH1]). Ultimately, increased angiogenesis in the infarct border zone of rats, reduced infarct size, and significant improvement in cardiac function were observed [333]. This also illustrates the role of ABs, one of the components of sEVs, in promoting angiogenesis.

#### Stem Cell-Derived EVs Protect Cardiomyocytes and Promote Cardiomyocyte Regeneration

Following MI, myocardial ischemia and necrosis activate various cell death mechanisms, including apoptosis, pyroptosis, and necrocytosis. sEVs derived from stem cells have been shown to induce cardiomyocyte protection by modulating these biological behaviors.

MiR-21a-5p is a typical exosomal miRNA secreted by BMMSCs, which significantly reduces cardiomyocyte death induced under oxygen-glucose deprivation (OGD) conditions. Studies have found that miR-21a-5p significantly downregulates the expression of pro-apoptotic genes such as PTEN, programmed cell death protein 4 (PDCD4), pelikan homolog 1 (Peli1), and Fas ligand (Fasl). Among them, miR-21a-5p promotes Akt activation by degrading PTEN protein, thereby reducing its dephosphorylation of PIP3. Additionally, miR-21a-5p inhibits apoptosis by reducing Fasl expression, preventing its binding to the Fas receptor, and decreasing caspase 8 activation. miR-21a-5p also inhibits NF-κB activation by targeting Peli1, reducing inflammation and apoptosis. PDCD4 is another target gene of miR-21a-5p, and miR-21a-5p reduces its protein expression level by targeting the 3’UTR of PDCD4, thereby inhibiting its pro-apoptotic effect [334]. sEVs derived from BMMSCs have also been shown to carry the E3 ubiquitin ligase (ITCH), which inhibits the expression of apoptosis signal-regulated kinase-1 (ASK1) through ubiquitination, thereby reducing apoptosis induced in cardiomyocytes under hypoxia [335]. ASK1 is a mitogen-activated protein kinase that is sensitive to oxidative stress and can further regulate cell death through the activation of p38-MAPK and JNK signaling pathways. GSK3β is also a key molecule in promoting apoptosis, and studies have found that hypoxia treatment increases the expression of miRNA-26a in MSC-Exos, which inhibits the expression of GSK3β and phosphorylated β-catenin (a mediator of the Wnt pro-apoptotic pathway), reducing ischemia-reperfusion injury to cardiomyocytes and infarct size [336]. CXCR4 is a G protein-coupled receptor that binds to SDF-1α and plays a key role in stem cell homing, migration, and survival. Studies have found that sEVs (ExoCR4) isolated from MSCs overexpressing CXCR4 under hypoxic conditions significantly reduce the number of apoptotic cardiomyocytes. This effect is achieved through the activation of the PI3 K/Akt pathway, characterized by upregulation of IGF-1 and pAkt levels in cardiomyocytes and downregulation of active caspase 3 levels. Additionally, ExoCR4 has been found to enhance the tube formation capacity of HUVECs in vitro, which is associated with upregulation of VEGF and can be inhibited by PI3 K inhibitors [337]. MiR-19a secreted by sEVs derived from hucMSCs has been shown to activate the AKT pathway and inhibit the activation of the JNK3/caspase-3 pathway by targeting and reducing the expression of SOX6. Caspase-3 is the execution protein of apoptosis, and its activation marks the irreversible stage of apoptosis. Studies have found that caspase-3/cleaved caspase-3 levels are significantly reduced under the action of miR-19a, indicating that miR-19a targets the AKT/JNK3/caspase-3 pathway to reduce apoptosis and protect cardiomyocytes from ischemic injury [338]. Circular RNAs secreted by sEVs derived from MSCs have also been shown to improve myocardial injury by affecting apoptotic pathways. Studies have found that circRNA_0002113 secreted by sEVs derived from BMMSCs acts as a “sponge” to adsorb miR-188-3p, which inhibits the expression of the RUNX1 gene by targeting its 3’UTR. RUNX1 is a transcription factor whose nuclear translocation is closely related to apoptosis. Studies have found that under the action of circRNA_0002113, the expression of the downstream deubiquitinase USP7 and p53 and BAX of RUNX1 is significantly reduced, the infarct size in rats with MI is significantly reduced, and serum cTnT levels are decreased [339]. This indicates that the circRNA_0002113/miR-188-3p/RUNX axis plays an important role in post-MI repair by affecting the USP7/p53 pathway.

sEVs derived from MSCs can inhibit molecules related to pyroptosis, thereby reducing cardiomyocyte death in infarcted rats. Pyroptosis is an inflammatory form of programmed cell death characterized by the disruption of cell integrity and the release of inflammatory cytokines. Long non-coding RNA (lncRNA) KLF-AS1 has been shown to be significantly expressed in MSCs-Exs. lncRNAs can influence gene expression through multiple processes, including transcriptional regulation (such as binding to promoter regions), post-transcriptional regulation (such as affecting mRNA stability), and epigenetic regulation (such as recruiting chromatin-modifying complexes). Studies have found that KLF-AS1 significantly inhibits the expression of pyroptosis-related factors in cardiomyocytes (such as NLRP3, ASC, caspase-1, and gasdermin D [GSDMD]) and inflammatory cytokines IL-1β and IL-18. It achieves this by acting as a molecular sponge for miR-138-5p, which in turn targets the silencing information regulator 2-related enzyme 1 (Sirt1), ultimately reducing the infarct size in rats [340]. NLRP3 induces the formation of mature caspase-1 and the activation of IL-1β and IL-18, which can create a pro-pyroptotic microenvironment. It has been found that miR-100-5p secreted by sEVs derived from hucMSCs can target and bind to FOXO3, an upstream molecule of NRLP3, thereby inhibiting the formation of the pyroptosis microenvironment and protecting cardiomyocytes from pyroptosis under hypoxia/reoxygenation (H/R) conditions [341].

Recent studies have found that ferroptosis and copper overload may also contribute to cardiomyocyte death following MI. miR-23a-3p secreted by sEVs derived from hucMSCs has been shown to potentially target divalent metal transporter 1 (DMT1), thereby regulating cardiomyocyte ferroptosis and alleviating myocardial injury [342]. In addition, genes related to copper overload and ferroptosis (cuproptosis- and ferroptosis-related genes, CFRGs) have been identified in infarcted tissue, proving that they may be involved in post-MI tissue remodeling [343]. Further research is needed to elucidate the possible signaling pathways and sEVs-carrying molecules with targeting effects.

Endoplasmic reticulum (ER) stress and mitochondrial damage represent the subcellular manifestations of cardiomyocyte death following MI. ER stress is characterized by the accumulation of unfolded or misfolded proteins in cardiomyocytes, which can lead to apoptosis and tissue fibrosis [344]. It has been found that sEVs derived from hucMSCs can respond to ER stress-induced apoptosis in cardiomyocytes through the PI3 K/Akt signaling pathway, with significant reductions in the levels of apoptosis-related proteins such as Bax and cleaved-caspase-3 [345]. The significantly upregulated oxidative stress response post-MI and excessive ROS exacerbate mitochondrial homeostasis and mitochondrial DNA damage, potentially leading to more extensive and severe cardiomyocyte injury [346]. sEVs derived from MSCs have been found to protect mitochondrial function and reduce apoptosis. It has been discovered that sEVs derived from BMMSCs overexpressing macrophage migration inhibitory factor (MIF) can reduce ROS levels in infarcted rats and inhibit excessive mitochondrial fission and apoptosis in damaged cardiomyocytes. Further mechanistic studies have found that MIF-BMMSC-exos exert cardioprotective effects by activating AMPK [347]. sEVs derived from BMSCs co-cultured with CSCs have been shown to alleviate oxidative stress-related damage in CSCs by carrying miR-124 and inhibiting the expression of calcium/calmodulin-dependent protein kinase II (CaMKII) [348]. CaMKII can activate the mitochondrial apoptotic pathway, enhance the oxidative stress response, increase membrane permeability, release cytochrome c, and induce apoptosis [349].

In addition to inhibiting cardiomyocyte injury, sEVs derived from stem cells have also been shown to induce the transdifferentiation of non-cardiomyocytes, promoting cardiomyocyte regeneration and thereby improving myocardial remodeling, a process known as “direct lineage conversion”. Studies have demonstrated that by co-culturing mouse embryonic fibroblasts with sEVs isolated from the embryoid body formation stage (Emb-EVs) and the mesoderm induction stage (Mes-EVs), fibroblasts began to contract spontaneously from day 12 and formed cell clusters resembling cardiomyocytes, characterized by the expression of cardiomyocyte-specific markers (α-actinin, α-MHC, cTnT, etc.) and the appearance of sarcomeric structures. Mechanistic investigations suggest that the miR-290 family, which is enriched in Emb-EVs and associated with pluripotency and early differentiation, may initiate the reprogramming process of fibroblasts, while the highly expressed miR-1 and miR-133 in Mes-EVs play key roles in cardiomyocyte differentiation and maturation. Moreover, when these sEVs were injected into a mouse model of MI, sEVs treatment significantly reduced infarct size and improved mouse cardiac function [350]. Further elucidation revealed that when co-cultured with sEVs secreted by epicardial adipose tissue-derived stem cells (EATDS), porcine fibroblasts showed significant upregulation of key transcription factors promoting cardiomyocyte differentiation and maturation, such as GATA4, Nkx2.5, iroquois homeobox protein (IRX4), and TBX5, indicating that fibroblasts were undergoing a reprogramming process. Although the key molecules in sEVs promoting this transdifferentiation process were not identified, the study used single-cell RNA sequencing to identify a subpopulation of EATDS with great healing potential, which was shown to secrete molecules such as Galectin-1 (regulating immune responses and cell cycle), Peroxiredoxin 2 (encoding antioxidant enzymes), and CCL2 (monocyte chemoattractant protein) to construct a microenvironment conducive to myocardial repair [351]. These findings also provide important evidence for the clinical translation of sEVs.

#### Immunomodulatory Effects of Stem Cell-Derived EVs

In MI, TLR4 serves as a key mediator of pro-inflammatory signaling, and its activation triggers inflammatory responses that exacerbate myocardial injury. Studies have shown that sEVs derived from MSCs highly express miR-182-5p, which targets TLR4, significantly reducing its mRNA and protein expression levels and further decreasing the phosphorylation level of p65, a marker of NF-κB signaling pathway activation. sEVs overexpressing miR-182-5p can significantly inhibit the expression of inflammatory cytokines (such as IL-6, IL-1β, TNF-α, and MCP-1) in myocardial tissue and significantly improve cardiac function, characterized by reduced left ventricular end-diastolic diameter (LVEDD) and left ventricular end-systolic diameter (LVESD), as well as increased LVEF and left ventricular fractional shortening (LVFS) [352]. Additionally, miR-129-5p expressed by MSC-derived sEVs has been shown to target HMGB1, inhibiting its binding to TLRs and the activation of pro-inflammatory cascades, thereby suppressing inflammation, cardiomyocyte apoptosis, and fibrosis in infarcted mice [353].

sEVs derived from MSCs have also been found to suppress myocardial inflammatory responses and promote the transition from the inflammatory phase to the fibroproliferative phase. As previously mentioned, during the inflammatory phase, fibroblasts are activated and acquire a pro-inflammatory phenotype, releasing inflammatory mediators while inhibiting their transdifferentiation into myofibroblasts. Myofibroblasts, which possess anti-inflammatory properties and secrete extracellular matrix proteins to maintain the integrity of cardiac structure and function, play a crucial role in this transition. Studies have shown that sEVs derived from hucMSCs can significantly increase the density of myofibroblasts in the infarct area and enhance the collagen gel contraction capacity of fibroblasts on postoperative day 2 in a rat model of MI, without significantly affecting their migration and proliferation. Moreover, sEVs from hucMSCs significantly reduced the expression of inflammatory cytokines such as IL-1β and TNF-α in fibroblasts [354], highlighting their important role in inflammation suppression.

In the inflammatory microenvironment, the functional and phenotypic changes of immune cells are crucial for regulating inflammatory responses and promoting cardiac repair. Tregs play an important role in inhibiting the activation of the immune system. Studies have found that sEVs derived from MSCs can induce the differentiation of Tregs, thereby promoting cardiac repair. When hucMSCs were injected into the pericardium of infarcted animals (immune cells in the pericardium migrate to the mediastinal lymph nodes and lead to immune activation after cardiac injury), it was found that sEVs were taken up by MHC-II^+^ antigen-presenting cells (APCs) and promoted the dephosphorylation of Foxo3 by activating the PP2 A/p-Akt/Foxo3 pathway, thereby upregulating the expression of anti-inflammatory cytokines such as IL-10, IL-33, and IL-34, and promoting Tregs differentiation. This treatment ultimately significantly improved cardiac function and reduced the size of MI [355]. Macrophages undergo the most typical and dramatic phenotypic changes in the inflammatory microenvironment, and sEVs have been shown to play an important regulatory role in this process. Studies have found that miR-24-3p secreted by sEVs derived from hucMSCs can enhance the polarization of macrophages towards the M2 phenotype by inhibiting the activation of phospholipase Cβ3 (Plcb3) and NF-κB signaling pathway-related molecules [356]. sEVs secreted by ADMSCs have been found to reverse MI-induced M1 macrophage polarization and promote M2 polarization. ADMSC-sEVs have also been shown to activate the sphingosine-1-phosphate (S1P)/sphingosine kinase 1 (SK1)/sphingosine-1-phosphate receptor 1 (S1PR1) signaling pathway, thereby inhibiting the expression of NF-κB p65 and TGF-β1 [357], ultimately reducing myocardial inflammatory responses and myocardial fibrosis.

#### Antifibrotic Effects of Stem Cell-Derived EVs

Studies have shown that sEVs derived from stem cells play an important role in the regulation of myocardial fibrosis following MI, primarily by targeting key fibrotic signaling pathways such as TGF-β/Smad and Angiotensin II/AT1R. sEVs secreted by adMSCs have been proven to inhibit the expression of TGFBR2 and the phosphorylation of Smad2 by transferring miR-671, thereby alleviating myocardial fibrosis. In a mouse model of MI, this effect is manifested as a reduction in myocardial collagen fiber deposition and a significant decrease in α-SMA protein expression. In addition, sEVs treatment can also significantly reduce the levels of inflammatory cytokines such as IL-6 and TNF-α in myocardial tissue and significantly decrease the rate of cardiomyocyte apoptosis, demonstrating their synergistic anti-inflammatory and anti-apoptotic effects [358]. sEVs secreted by BMSCs carry miR-212-5p, which can inhibit the expression of Nod-like receptor family caspase recruitment domain-containing 5 (NLRC5) and target fibroblasts that play a key role in myocardial fibrosis. NLRC5 has been shown to further promote the expression of VEGF, which in turn promotes cardiac fibrosis by activating the TGF-β1/Smad pathway and affects cardiac function [359]. The renin-angiotensin system (RAS) plays a significant role in cardiac remodeling post-MI. The RAS consists of two regulatory axes: the ACE-Angiotensin II-AT1R axis, which promotes fibrosis, and the ACE2-Angiotensin 1–7-Mas axis, which counteracts fibrosis. Studies have found that sEVs released by third-generation BMSCs can induce the expression of ACE2 in the plasma of rats with MI, thereby accelerating the conversion of Angiotensin II to Angiotensin 1–7, reducing collagen deposition in the myocardium, and improving cardiac function in rats [360].

### Clinical Studies of Stem Cell Therapy for MI

The application of cell therapy in clinical medicine initially focused primarily on the treatment of MI. During MI, a large number of cardiomyocytes are lost, and the heart’s compensatory capacity is insufficient. The goal of cell therapy is to use coronary infusion of stem cells as an adjunct to PCI to limit cardiomyocyte necrosis and prevent the progression of the disease to heart failure, a strategy also known as “cardioprotection” [361].

Left ventricular remodeling is an independent predictor of cardiovascular mortality and is used as an important marker of clinical efficacy in cardiovascular treatments [362]. Characteristics of remodeling include gradual thinning of the ventricular wall, fibrosis, and left ventricular dilation, which can be measured by LVESV, LVEDV, infarct size, and chamber dimensions [363]. The effects of stem cell therapy are reflected in the reduction of left ventricular volume, sphericity index, infarct size, and scar size. Additionally, improved myocardial perfusion due to angiogenesis (measured by single-photon emission computed tomography [SPECT] and cMRI) is also used as an efficacy indicator [364]. Most stem cell-based MI treatments have assessed their myocardial repair effects using the aforementioned remodeling indicators.

#### Stem Cell-based Clinical Studies

BMMNCs are the earliest and most widely used cell product in the context of MI, with the advantage of not requiring in vitro culture expansion and the ability to rapidly complete autologous or allogeneic collection, separation, and reinfusion. Early clinical studies have reported that the application of BMMNCs can improve LVEF in the short term and improve indicators related to cardiac remodeling (Table 3). For example, the TOPCARE-AMI (Transplantation of Progenitor Cells and Regeneration Enhancement in Acute Myocardial Infarction) trial and the BOOST (BOne Marrow transfer to enhance ST-elevation infarct regeneration) trial both injected BMMNCs into the coronary arteries of patients 5 days after AMI (TOPCARE-AMI also included groups infused with CPCs and BMCs) and followed up for 4–6 months, finding that BMMNC injection was beneficial for improving LVEF, reducing ESV, and decreasing the size of myocardial infarction [365, 366]. However, TOPCARE-AMI was a single-arm study, and BOOST was an open-label study. Subsequent Leuven-AMI and FINCELL studies further confirmed the effect of BMMNCs infusion on LVEF improvement through double-blind design [375, 376]. The REPAIR-AMI (REinfusion of ENriched Progenitor cells and Infarct Remodeling in Acute Myocardial Infarction) study is a Phase III, double-blind, placebo-controlled clinical trial and the largest clinical trial to date for AMI. The study found that BMMNCs treatment could improve LVEF in the short term (4 months), and after 1 year of follow-up, the incidence of death, MI, and revascularization was lower in the BMMNCs group, with the advantage in the hard endpoint of mortality maintained up to 5 years after the start of the study [367].

**Table 3:** Clinical studies of stem cell therapy after MI

Trial	Dose	Cell delivery	indication	*n*	Follow-up (month)	Death	MACEs	Evaluation method	LVEF	Volumes	Scar size
TOPCARE-AMI [365]	BMMNC: 213 × 10^6^; CPC: 16 × 10^6^	Intracoronary during PCI	STEMI	59 (CPC: 30; BMC: 29)	12	NR	NR	LVG	↑	ESV ↓	↓
BOOST [366]	BMMNC: 2.5 × 10^9^ vs. control	intracoronary 4.8 days after PCI	STEMI	60 (BMMNC: 30; control: 30)	6	NR	NS	cMRI	↑	NS	NA
REPAIR-AMI [367]	BMMNCs: 198 × 10^6^ vs. placebo	intracoronary 3–7 days after PCI	AMI	204 (BMC: 101; control: 103)	4	↓(12 month)	↓ (12 month)	LVG	↑	NS	NA
TIME [368]	BMMNCs: 150 × 106 vs. placebo	intracoronary 3 vs. 7 days after PCI	STEMI	120 (BMC: 79; placebo: 41)	6	NA	NA	cMRI	NS	NS	NS
LateTIME [369]	BMMNCs: 150 × 106 vs. placebo	intracoronary 2–3 weeks after PCI	AMI	87 (BMMNC: 58; placebo: 29)	6	NA	NA	cMRI	NS	NS	NS
BOOST−2 [370]	BMMNC: 20.6 × 10^8^ vs. 7.0 × 10^8^ vs. placebo	intracoronary 8 days after PCI	STEMI	153 (loBMC/loBMCi: 71; hiBMC/hiBMCi: 64; control: 26)	6	NA	NA	cMRI	NS	NS	NS
PreSERVE-AMI [371]	CD34 ^+^ cells: 14.9 × 10^6^ vs. placebo	intracoronary 4−11 days after PCI	STEMI	161 (CD34 ^+^ cells: 78; placebo: 83)	6	NS	NS	cMRI	NS	NS	NS
Hare et al. [372]	hMSC: 0.5/1.6/5.0 × 10^6^/kg vs. placebo	intravenous 1–10 days after MI	MI	60 (hMSC: 10/10/10; placebo: 5/5/11)	6	NR	NS	cMRI	NS	NS	NA
Gao et al. [373]	WJMSCs: 6 × 106 vs. placebo	intracoronary 5–7 days after PCI	STEMI	116 (WJMSC: 58; placebo: 57)	18	NR	NS	Echo/cMRI	↑	↓	NA
CAREMI [374]	AlloCSC−01: 35 × 106 vs. placebo	intracoronary 5–7 days after STEMI	STEMI	49 (AlloCSC−01: 33; placebo: 16)	12	NR	NR	cMRI	NS	NS	NS

*NS* not significant; *NA* not associated or not compared; *NR* not reported; *MACEs* major adverse cardiovascular events

Despite these encouraging results, other trials have reached conflicting conclusions. The TIME (Timing of Adult Stem Cell Infusion in Acute Myocardial Infarction) and LateTIME (Timing of Adult Stem Cell Infusion in Patients with Myocardial Infarction 2 to 3 Weeks Post-Infarction) studies, initiated by the Cardiovascular Cell Therapy Research Network (CCTRN), are multicenter, double-blind, placebo-controlled clinical trials. The findings revealed that infusion of BMMNCs either 3–7 days post-MI or 2–3 weeks post-MI did not result in improvements in LVEF, left ventricular volumes, or wall motion function as shown by cMRI during the 6–12 month follow-up period [368, 369]. The BOOST-2 trial, building on previous research, optimized the trial design by administering low-dose BMCs (7.0 × 10^8^), high-dose BMCs (20.6 × 10^8^), low-dose and high-dose irradiated BMCs (via Ɣ-irradiation to eliminate clonogenic capacity, to discern whether BMC therapy efficacy relies on long-term cell survival and differentiation or acts through paracrine mechanisms) intracoronary 8 days post-PCI on average in patients with STEMI. The study found that during the 6-month follow-up, BMCs infusion did not show significant effects on LVEF and other indicators compared to the control group, and no significant differences were observed among the BMCs groups [370]. Given that BMMNCs represent an unselected cell population, the PreSERVE-AMI trial investigated the comparative effects of CD34^+^ cells isolated from bone marrow versus placebo infused 4–11 days post-PCI. The results indicated that improvements in LVEF, infarct size, and survival rate only became apparent after correction for ischemic duration and exhibited a cell-dose dependency [371], suggesting that this purified cell population may exert beneficial effects.

The most notable characteristic of the aforementioned BMMNCs-based cell trials is their high degree of heterogeneity. This may be attributed to the inherent heterogeneity of the BMMNC cell population itself, such as the variable quality of cells derived from patients of different ages and pathological conditions. Additionally, the impact of different cell isolation and storage media, cell processing times on their bioactivity, and variations in cell dosage and injection timing may also contribute to different outcomes. This highlights the importance of adopting standardized collection protocols and processing techniques in clinical trials and the need for standardized trial protocols in comparative efficacy studies.

Given the limited efficacy demonstrated by BMMNCs in clinical trials, researchers have attempted to use different cell types to explore their potential clinical roles in MI treatment. Hare et al. were the first to administer allogeneic hMSCs intravenously to patients with AMI, yielding encouraging results. The study demonstrated that intravenous injection of MSCs is not only safe but also significantly improves LVEF, particularly in patients with anterior wall infarction. Moreover, the incidence of arrhythmias in patients treated with hMSCs was significantly lower than in the placebo group [372]. Considering that the activity and function of autologous adult stem cells decline with age, Gao et al. conducted a multicenter, randomized, double-blind, placebo-controlled trial involving 116 AMI patients, who were randomly assigned to receive intracoronary infusion of WJMSCs or placebo. The study results showed significant improvements in LVEF, LVESV, and LVEDV absolute values, as well as myocardial viability and perfusion in the WJMSCs group [373]. The CAREMI trial was the first clinical trial to use allogeneic cardiac stem cells (AlloCSC-01) in AMI patients. The study enrolled 49 STEMI patients and administered intracoronary infusion of 35 × 10^6^ AlloCSC-01 cells to the intervention group. These cells were isolated from cardiac biopsy tissue of healthy donors and underwent immunoselection and expansion (excluding CD45^+^ and selecting c-kit^+^ cells). The study reported that intracoronary infusion of allogeneic cardiac stem cells in STEMI patients was safe, but no significant differences were observed between the two groups in terms of infarct size and LVEF [374].

#### Mechanism-Descriptive Clinical Studies

Cell therapy has made certain progress in clinical studies of cardiac remodeling following MI, but the research findings have presented a complex and diverse picture, with both encouraging discoveries and less-than-satisfactory results, as elaborated in the previous sections. Despite these advancements, the specific mechanisms by which cell therapy exerts its therapeutic effects remain incompletely understood, which to some extent limits the further optimization of trial protocols and the translation and application of new mechanisms. Therefore, this study further retrieved and analyzed major clinical trials based on mechanistic descriptions, aiming to reveal the scientific basis and potential breakthroughs of stem cell therapy, providing a more solid theoretical foundation for future research and clinical applications.

#### Granulocyte Colony-Stimulating Factor (G-CSF) Promotes Stem Cell Mobilization

In addition to the infusion of BMMNCs ex vivo, the largest number of clinical studies have focused on optimizing the mobilization of endogenous BMMNCs and their homing to the infarct region. During MI, bone marrow-derived progenitor cells are mobilized into the peripheral blood and home to ischemic myocardium to promote angiogenesis and tissue repair [377, 378]. G-CSF is an endogenous hematopoietic cytokine that mobilizes granulocytes, stem cells, and progenitor cells from the bone marrow into the bloodstream. Moreover, G-CSF has been found to directly activate pro-survival signaling pathways in ischemic cardiomyocytes [379]. Therefore, G-CSF has become one of the most extensively studied molecules in mechanistic-based clinical trials.

The MAGIC-Cell series of studies were the earliest clinical trials to explore the use of G-CSF to mobilize stem cells and repair damaged myocardium following MI (Table 4). In the early phase of the study, 27 subjects were enrolled and randomly divided into three groups: the cell infusion group (*n* = 10), the G-CSF monotherapy group (*n* = 10), and the control group (*n* = 7). The cell infusion group first received subcutaneous injections of G-CSF at a dose of 10 µg/kg for 4 days. During this period, mobilized peripheral blood stem cells (PBSCs) were collected. Subsequently, PBSCs were infused intracoronary during PCI treatment. The other two groups received subcutaneous G-CSF injection + PCI or PCI alone, respectively. The study found that the infusion of mobilized PBSCs intracoronary in AMI patients was feasible. During the 2-year follow-up, it was found that this significantly improved patients’ exercise tolerance, LVEF, and myocardial perfusion. However, a re-stenosis rate of nearly 1/2 to 2/3 was observed in the G-CSF treatment group, which led to the early termination of the study and became the main concern of the research team [380, 382]. To address this issue, the research team attempted to first perform PCI intervention, followed by subcutaneous injection of G-CSF and collection of mobilized PBSCs, and finally explored the therapeutic effects through intracoronary infusion. The results showed that the infusion of mobilized PBSCs significantly improved LVEF in MI patients and reduced the incidence of revascularization events, especially in patients with diabetes. However, significant improvements were only observed compared to baseline in LVESVI and infarct size. In addition, it was found that PBSCs infusion may be more effective in AMI patients than in patients with old myocardial infarction (OMI), and this effect persisted during the 2-year follow-up [381, 384]. Based on this finding, the team enrolled 74 patients to compare the differences in PBSCs collected from AMI and OMI patients. Notably, the comparison revealed higher collection efficiency of PBSCs in AMI patients, with a richer G-CSF-mobilized EPCs population, and significantly higher proportions of VE-cadherin^+^ cells and CD34/KDR double-positive cells in the AMI group compared to the OMI group [386]. This indicates that the efficiency of G-CSF-mobilized stem cells is affected by the timing of mobilization, and it may be easier to mobilize higher-quality stem cell populations during the acute phase. Furthermore, the study evaluated the impact of G-CSF-mobilized PBSCs on coronary vessels by implanting drug-eluting stents. The results demonstrated that G-CSF-mobilized stem cells do not increase the intimal area but have a positive effect on the average stent surrounding immune response, thereby promoting coronary vessel remodeling [383]. With the in-depth exploration of the therapeutic mechanisms of cell therapy, the team launched the MAGIC cell-5-combination cytokine trial in 2011, aiming to explore the impact of G-CSF combined with darbepoetin on cardiac remodeling following MI. Darbepoetin is a long-acting erythropoiesis-stimulating agent that has been shown to protect cardiomyocytes from ischemia-induced necrosis or apoptosis and induce angiogenesis by stimulating EPCs [385]. The findings of this trial will provide new research directions and scientific basis for the mobilization of BMSCs to promote myocardial remodeling.

**Table 4:** Clinical studies based on mechanistic descriptions of G-CSF

Trial	Dose	Cell delivery	indication	number	Follow-up (month)	Death	MACEs	LVEF	LVESVI	Scar size
MAGIC Cell [380]	10 ug/kg, once a day*4 days	PBSCs collected at the 5^th^ day of injection(started after DES), intracoronary	AMI	27 (PBSCs: 10; G-CSF: 7; control: 7)	6	NR	NR	↑	NA	NA
MAGIC Cell−3-DES [381]	10 ug/kg, once a day*3 days	PBSCs collected at the 5^th^ day of injection(started after DES), intracoronary	AMI, OMI	96 (AMI + PBSCs: 25; AMI control: 25; OMI + PBSCs: 16; OMI control: 16)	6	NR	NR	↑	NS	NS
MAGIC Cell 1 [382]	10 ug/kg, once a day*4 days	PBSCs collected at the 5^th^ day of injection, intracoronary	AMI, OMI	30(PBSCs: 10; G-CSF: 10; control: 10)	24	NR	NR	↑	↑	NA
MAGIC Cell−3-DES (sirolimus-eluting or paclitaxel-eluting stent) [383]	10 ug/kg, once a day*3 days	PBSCs collected at the 4^th^ day of injection, intracoronary	AMI	36(PBSCs: 19; control: 17)	6	NA	NA	NA	NA	NA
MAGIC Cell−3-DES [384]	10 ug/kg, once a day*3 days	PBSCs collected at the 4^th^ day of injection, intracoronary	AMI, OMI	163(AMI + PBSCs: 57; AMI control: 60; OMI + PBSCs: 22; OMI control: 24)	30	NS	↓	NS	NA	NA
MAGIC cell−5-combination cytokine trial [385]	5 ug/kg; twice a day*3 day; 4.5 ug/kg	PBSCs collected at the 4^th^ day of injection, intracoronary; darbepoetin after PCI, intravenous	STEMI	116(PBSCs + darbepoetin: 58; PBSCs: 29; control: 29)	6	NR	NR	NR	NR	NR

*NS* not significant; *NA* not associated; *NR* not reported

STEM-AMI OUTCOME (Prognosis of Stem Cell Mobilization in Acute Myocardial Infarction) is the largest prospective, multicenter, randomized, open-label Phase III clinical trial in this field to date. The study enrolled 532 patients with STEMI and assessed the effects of subcutaneous injection of 5 µg/kg G-CSF 24 h post-PCI on cardiac function. CMR evaluation revealed significant improvements in LVEF, left ventricular end-systolic volume index (LVESVI), infarct size, and 2D global longitudinal and circumferential strain in patients receiving G-CSF. Additionally, the study included a subset of high-risk patients, defined as those with persistent left ventricular dysfunction (LVEF ≤ 45%) following successful reperfusion, who were considered at higher risk for adverse left ventricular remodeling. The results indicated that G-CSF could serve as an effective therapeutic option for these patients [387]. The study also conducted stratified analyses to explore the impact of different degrees of bone marrow cell mobilization on cardiac function. It was found that patients with low bone marrow cell mobilization (leukocyte count ≤ 50 × 10^9^/L) had a lower trend in the composite primary endpoint compared to those with high mobilization (leukocyte count > 50 × 10^9^/L), although the results were not statistically significant (*p* = 0.06) [388].

The REVIVAL-2 study enrolled 114 patients with AMI and administered 10 µg/kg G-CSF subcutaneously once daily to the intervention group starting on day 5 post-PCI. The study found significant increases in CD34^+^ cells and leukocytes, with peak levels observed on day 5 of treatment. However, no significant differences were observed between the two groups in terms of LVEF, infarct size, or re-stenosis rate [389]. The study speculated that the mobilized stem cells might not have effectively homed to the infarcted myocardium, or they might have been too immature to participate effectively in myocardial repair. Alternatively, the timing of G-CSF administration might not have aligned with the optimal therapeutic window post-MI. Four years later, the team published mechanistic study results, analyzing venous blood samples collected on days 1, 3, 5, and 7 post-G-CSF injection. They found that G-CSF significantly reduced the expression of progenitor cell surface molecules lymphocyte function-associated antigen 1 (LFA-1), very late antigen 4 (VLA-4), and CXCR4. At the mRNA level, it also reduced the expression of CXCR4 and the anti-proliferative protein transducer of Erk-binding protein (TOB) [390]. Adhesion molecules (such as LFA-1, VLA-4, and CXCR4) are key molecules in the bone marrow microenvironment of progenitor cells, maintaining stem cell retention through binding to ligands (such as VCAM-1, ICAM-1, and SDF-1). Disrupting these interactions has been shown to mobilize progenitor cells into the peripheral circulation [391]. However, these changes might also have weakened the homing capacity of progenitor cells to cardiomyocytes, despite their potentially higher proliferative potential. These findings also explain why G-CSF failed to improve infarct size and left ventricular function in AMI treatment. The study also provided 7-year follow-up results, with 106 patients completing the follow-up. The long-term results were consistent with the short-term findings, indicating that although G-CSF alone could mobilize BMSCs, it had no significant impact on the long-term clinical outcomes (including mortality, recurrent MI, and stroke) of AMI patients [392]. The TECAM trial and a study by Guo et al. reached similar conclusions, suggesting that subcutaneous injection of G-CSF might not significantly improve cardiac function or left ventricular remodeling in AMI patients. The former also found no significant differences when compared to the use of BMMNCs alone or in combination with BMMNCs [393, 394].

The CAPITAL STEM MI trial compared the efficacy of subcutaneous injection of G-CSF on days 3 or 4 after PCI in patients with AMI, but it was found that patients in the G-CSF group had a significant decrease in LVEF [395]. The study suggested that this might be attributed to the selection of study subjects, as the trial included patients with moderate to severe left ventricular dysfunction (LVEF < 45%), who might be more sensitive to the application of G-CSF. Additionally, compared with previous studies that initiated G-CSF treatment within 24 h after PCI, G-CSF treatment in this study was started within 4 days after MI, and this delay might have affected the therapeutic effect, indicating the significant impact of the timing of treatment on efficacy. The study also pointed out that G-CSF might activate neutrophils, thereby triggering inflammatory reactions or thrombosis, which could have a negative impact on the already impaired myocardial function. Moreover, the failure of stem cell mobilization to effectively participate in myocardial repair might also be a reason for the lack of positive results in the trial. Further mechanistic exploration is needed to explain these findings.

The FIRSTLINE-AMI trial was the first to initiate subcutaneous injection of G-CSF within 90 min after PCI to improve ventricular remodeling, and the results showed that it could significantly improve LVEF in patients with AMI, but had no significant effect on LVESVI [396]. The G-CSF-STEMI trial included patients with subacute MI and initiated G-CSF treatment on day 1 after PCI. The results indicated that although there were no significant differences in LVEF and MACEs between the G-CSF group and the control group, G-CSF treatment significantly improved resting myocardial perfusion in the infarct area within 1 month [397]. This suggests that G-CSF might enhance angiogenesis in the infarct area, thereby participating in the promotion of ischemic myocardial repair.

Meanwhile, focusing on the impact of G-CSF-mobilized stem cell subpopulations on cardiac function recovery, the STEMMI trial enrolled 78 STEMI patients who were randomly assigned to receive G-CSF (10 µg/kg/day for 6 days) or standard treatment after PCI, and characterized the mobilized stem cells. The results showed that after G-CSF mobilization, the number of CD34 + cells and mesenchymal stem cells in peripheral blood of patients significantly increased, but the proportion of MSCs was inconsistent. For example, the number of MSCs positive for CD73 (an early stem cell marker) and CD31 (an endothelial marker) was significantly lower than that in the control group. Moreover, it was found that the number of circulating MSCs in peripheral blood was negatively correlated with changes in LVEF. The reason for this phenomenon is considered to be that these peripheral blood MSCs subpopulations with lower proportions might more easily home to the infarcted myocardium, thereby effectively participating in the repair process [398]. Additionally, this selective mobilization characteristic of G-CSF also provides clues for further exploration of molecular mechanisms and the conduct of clinical trials, such as improving the mobilization ability of certain cells that are more likely to home to the infarct area, thereby enhancing mobilization efficiency.

G-CSF as a potent endogenous hematopoietic cytokine can effectively mobilize stem cells into the bloodstream and is theoretically beneficial for the repair of damaged myocardium. However, results from multiple clinical studies indicate that the efficacy of G-CSF may be significantly influenced by various factors, including the timing of G-CSF injection, the administration method of the mobilized cells, and the cell source. Future research that standardizes these key factors or explores their combination with other means (such as erythropoietin [EPO] and other pro-cellular factors [399]) may provide new research directions for improving ventricular remodeling post-MI and lead to more desirable therapeutic outcomes.

#### Other Mechanisms-descriptive Clinical Studies

The MAGNUM clinical trial enrolled 15 patients scheduled for off-pump coronary artery bypass grafting (CABG). During surgery, autologous BMCs were injected into the myocardial scar region while covering and securing a 3D collagen type I matrix with sutures to provide a microenvironment conducive to cell survival, proliferation, and differentiation. The study found that this approach was safe, improved myocardial perfusion, limited ventricular remodeling, and enhanced patients’ cardiac function classification [400], which is of great significance for improving patients’ quality of life. Notably, the thickness of the myocardial scar region significantly increased, indicating that the use of this bioartificial myocardium may facilitate the formation and maturation of fibrous scars and reduce the occurrence of serious adverse events such as cardiac rupture. However, this small-scale study lacked a control group, so it could not definitively attribute the improvements to cell therapy or surgery. This question awaits answers from larger-scale randomized controlled studies in the future.

The use of ATV in combination with stem cells for the treatment of AMI has been extensively explored in preclinical studies. The TEAM-AMI trial enrolled 100 patients with anterior STEMI and compared the efficacy of conventional ATV (20 mg/day) and intensive ATV (80 mg/day) in combination with autologous bone marrow mesenchymal stem cells (MSCINJ) versus placebo injection. The primary endpoint was the change in LVEF, with secondary endpoints including parameters related to cardiac function, remodeling, and regeneration. The results of this study will provide more substantial evidence for the efficacy of ATV in improving post-MI myocardial remodeling, that is, whether improving the inflammatory environment can promote the function of stem cells in repairing myocardial tissue, and will also provide clear indicators for the adjunctive dosage of ATV [362, 363]. Previously, the STRAP study found that patients receiving intensive treatment (taking 80 mg of ATV daily from admission) had significantly higher EPC counts at 4-month follow-up compared to standard treatment, indicating that ATV treatment also aids in the mobilization of bone marrow stem cells [401].

PeriCord is an engineered tissue graft composed of decellularized pericardial matrix and WJ-MSCs. During CABG, PeriCord was fixed to the scar surface with surgical glue in MI patients. The study found that PeriCord successfully integrated into the myocardium without causing immune rejection and significantly modulated the proportion of peripheral blood mononuclear cell subpopulations, characterized by an increase in the number of non-classical anti-inflammatory monocytes (CD14^−^CD16^+^). Additionally, the plasma levels of Metrnl were significantly reduced post-PeriCord implantation [402], indicating its key role in immune regulation.

Thymosin β4 (Tβ4) is a small molecule protein with high affinity for EPCs, capable of inducing EPCs migration and protecting them from apoptosis induced by serum deprivation. Preclinical studies have confirmed that EPCs pretreated with Tβ4 can significantly increase vascular density and reduce infarct size in animal infarction tissues [403]. ZHU et al. were the first to attempt intracoronary infusion of EPCs pretreated with Tβ4 (1000 ng/mL) for 24 h into patients with STEMI and found that this method could significantly improve left ventricular function in patients, including LVEF and stroke volume (SV), and showed significant advantages in the 6-minute walk test of patients [404]. Although this was a small-scale pilot study, it provided a new direction for the pro-angiogenic effects in the myocardial infarction repair process. The ENACT-AMI (Enhanced Angiogenesis Cellular Therapy in Acute Myocardial Infarction) study aimed to evaluate the effect of intracoronary infusion of autologous early EPCs on cardiac function improvement. The study found that an important limitation of autologous cell therapy is the adverse effects of age and cardiac risk factors on progenitor cell activity [405]. eNOS can catalyze the synthesis of NO, which plays an important role in vascular endothelial function. Increased eNOS expression has been shown to promote vasodilation, improve local microcirculation, and promote angiogenesis, thereby reducing myocardial ischemia and infarct size [406]. The study cultured peripheral blood MNCs in vitro to form endothelial-like cells with high regenerative capacity (E-CMMs) and transfected them with eNOS after 4–6 days of culture, ultimately obtaining a new type of enhanced autologous progenitor cells. Patients received intracoronary infusion of 20 million autologous E-CMMs and their efficacy was assessed by measuring endpoints such as LVEF, regional wall thickness, infarct size, and MACEs [407]. ENACT-AMI is the first clinical study to combine gene modification with cell therapy for the treatment of AMI, and it is believed that its research results will endow the treatment of ventricular remodeling post-MI from the perspective of promoting angiogenesis with important clinical significance.

Preclinical studies have shown that ischemic preconditioning can significantly enhance the anti-apoptotic ability and hypoxia tolerance of stem cells, thereby making them more likely to survive and function effectively in the ischemic microenvironment post-MI. The CHINA-AMI study was a prospective, randomized, double-blind, placebo-controlled Phase I clinical trial, enrolling 22 patients with STEMI. Patients were randomly assigned to receive intracoronary infusion of normoxic BMCs (N-BMCs, *n* = 11) or hypoxia-preconditioned BMCs (HP-BMCs, *n* = 11) after routine reperfusion therapy, with an additional 14 patients receiving standard therapy as a control group. BMCs were processed by culturing for 24 h in normoxic (21% O_2_) or hypoxic (0.5% O_2_) environments, and the intracoronary infusion dose was 1 × 10^7^ BMCs. At 30-day and 1-year follow-ups, there were no significant differences in the incidence of MACEs among the three groups. SPECT showed that compared to baseline, the proportion of myocardial perfusion defects in the N-BMCs and HP-BMCs groups significantly decreased at 6 and 12 months. In addition, the LVEDV and LVESV in the HP-BMCs group were significantly improved at 6 and 12 months, and the wall motion score index (WMSI) significantly decreased at 6 months [408]. In addition, studies have assessed the effect of ischemic postconditioning (PostC) on EPCs recruitment in patients with STEMI. The study demonstrated that PostC has benefits in improving endothelial function and reducing infarct size [409]. The study enrolled 20 patients with STEMI and randomly assigned them to the PostC group and the conventional PCI group. In the PostC group, after successfully opening the infarct-related artery (IRA) and restoring blood flow, the vessel blood flow was completely occluded by inflating a balloon to 2–4 atmospheres, and the balloon was rapidly deflated after 60 s to restore blood flow for 60 s. The above operation was repeated four times in a cycle. The study found that there were no significant differences in the number of different EPC subpopulations in the PostC group compared to before the intervention, but the time from symptom onset to balloon inflation (re-occlusion treatment) was negatively correlated with the mobilization of progenitor precursor cells [410]. This suggests that EPCs recruitment may be related to ischemic time, and future studies need to further explore the specific mechanisms of PostC on EPCs recruitment and how to optimize this endogenous repair mechanism to improve the prognosis of patients with STEMI.

Combined cell therapy aims to enhance the efficacy of stem cells through complementary effects. A Phase I clinical study enrolled 15 subjects (combined cell therapy group: 10; control group: 5) and evaluated the efficacy of intracoronary infusion of the combined cell product (7.5 × 10^6^ MSCs and 7.5 × 10^6^ MNCs) on cardiac function in AMI patients. The results showed that intracoronary infusion of the combined cell product was safe and feasible and did not cause ischemic or infarction events in the injection area. In addition, in the treatment group, the baseline SDS score (i.e., myocardial perfusion difference score) was significantly higher than the score at 3 months, indicating a reduction in myocardial ischemia burden, and the use of nitroglycerin by patients was significantly reduced, with a significant improvement in quality of life [411].

From the formulation of clinical questions to an in-depth exploration of their underlying mechanisms, and ultimately to the resolution of these clinical issues, the process is complex and lengthy. In the field of MI treatment, cell therapy, as an emerging therapeutic modality, exemplifies this translational process. Initially, basic research revealed that stem cell therapy appears to have potential protective effects on ischemic myocardial tissue following MI, offering new hope for treating MI. Subsequently, a series of clinical trials further confirmed the safety of cell products in treating MI. However, despite the good tolerability of cell therapy in clinical applications, its effects on improving patient cardiac function and reducing cardiac tissue remodeling have been less than ideal. This outcome suggests that mere safety is not sufficient to meet the needs of clinical treatment, and the efficacy of cell therapy still requires further optimization.

To address this issue, researchers began to delve into the specific pathways by which cell therapy improves myocardial remodeling post-MI at the mechanistic level. In recent years, basic research has unveiled that cell therapy may exert its effects through multiple mechanisms, including promoting myocardial regeneration, facilitating scar maturation, modulating the immune environment, and enhancing angiogenesis. These discoveries provide a theoretical foundation for further optimizing cell therapy. However, current research still has limitations. For instance, although existing clinical studies have to some extent validated the potential mechanisms of cell therapy, the effects have not yet reached the ideal therapeutic goals. Moreover, a substantial number of preclinical studies still need to be further verified in clinical settings. Future research may need to further clarify the specific mechanisms of cell therapy in MI treatment, which may include intracellular components such as cytokines and extracellular vesicles, especially in the key aspects of myocardial repair and tissue remodeling. The preparation and application strategies of cell products have also been proven to be key factors affecting efficacy. Further validation of the efficacy and safety of cell therapy through multicenter, larger sample, randomized controlled studies will also provide high-quality results for cell therapy.

## Summary and Future Directions

### Stem Cell Therapy: Bridging the Gap between Aspiration and Reality

The application of stem cell therapy in post-MI myocardial remodeling primarily focuses on promoting cardiomyocyte regeneration to replace lost myocardial tissue due to infarction and reverse adverse myocardial remodeling processes. However, despite the expression of cardiomyocyte-specific markers by stem cell-derived CLCs, significant differences remain in morphology and function compared to mature cardiomyocytes. These cells fail to fully exhibit the contractile function and electrophysiological properties characteristic of cardiomyocytes, which are fundamental to their critical role in cardiac tissue. Thus, we are still far from achieving the regeneration of “truly functional cardiomyocytes”. Future research needs to delve into several key questions: (1) Functional validation: Can stem cell-derived CLCs exert functions similar to mature cardiomyocytes in vivo, including contractility, electrical conductivity, and metabolic activity? (2) Clinical translation: Do these cells function consistently with in vitro experimental results in the human body, and what are their long-term effects and safety profiles? (3) Immune compatibility: How can stem cell products be optimized to enhance their immunotolerance in the human body and reduce immune rejection?

#### Inflammatory Microenvironment: A New Target for Stem Cell Therapy

Early infiltration of inflammatory cells post-MI is a crucial step in tissue repair, involving the release and regulation of various pro-inflammatory cytokines (such as TNF-α, IL-1β) and chemokines (such as MCP-1, CXCL1). These inflammatory mediators not only participate in the clearance of necrotic tissue but also influence subsequent fibrosis and angiogenesis. Therefore, exploring the regulatory effects of stem cell therapy on the inflammatory microenvironment may provide a new direction for improving myocardial remodeling. MSCs, a vital component of stem cell therapy, have been shown to possess significant immunomodulatory properties, capable of regulating inflammatory responses by secreting anti-inflammatory factors (such as IL-10, TGF-β) and inhibiting pro-inflammatory cytokines (such as TNF-α, IL-6). However, current research primarily focuses on the mechanisms of action of MSCs, while the roles of other stem cell types (such as iPSCs) in the inflammatory microenvironment remain less clear. Additionally, how to maintain the balance between inflammation and repair, such as how to optimize anti-inflammatory effects through stem cell therapy to promote tissue repair without causing excessive fibrosis; or whether the combination of anti-inflammatory drugs and stem cell therapy can further improve the post-MI inflammatory microenvironment, may be potential future research directions.

#### Fibroblasts and the ECM: Key Players in Remodeling

Fibroblasts and the ECM play key roles at different stages post-MI. During the inflammatory phase, fibroblasts are activated, secreting pro-inflammatory factors and MMPs to participate in tissue degradation and repair. In the proliferative phase, fibroblasts are activated into myofibroblasts, synthesizing and depositing collagen to form scar tissue. In the maturation phase, fibroblasts gradually undergo apoptosis, collagen fibers cross-link, and the scar tissue matures. Although studies have shown that stem cell therapy can influence fibroblast activity and ECM remodeling, it is currently unclear whether stem cells have different mechanisms of action at these different stages. Therefore, how to achieve stage-specific intervention, that is, how to design targeted stem cell therapy strategies based on the characteristics of different stages post-MI to optimize tissue repair and remodeling, may be a key focus for future research.

#### Endothelial Cells: Dynamic Participants in Myocardial Remodeling

The dynamic changes of endothelial cells following MI are crucial for the recovery of cardiac function. Studies have shown that endothelial cells exhibit pro-inflammatory and pro-angiogenic properties during the inflammatory phase; they participate in the formation and maturation of new blood vessels during the proliferative phase; and their functions gradually stabilize during the scar maturation phase. Although research has described the dynamic changes of endothelial cells post-MI, focusing mainly on functional changes over time, it is not entirely accurate to simply categorize the dynamic evolution of endothelial cells into the inflammatory, proliferative, and maturation phases based on time points. This may also be due to the overlap between different functional stages of endothelial cells, which cannot be clearly distinguished. Moreover, as evidenced by the content of this paper, with the advancement of technology, we have been able to dynamically identify the functional and phenotypic changes of endothelial cells following MI. This undoubtedly brings more directions for the treatment of post-MI remodeling. For example, can we regulate the apoptotic phenotype and metabolic changes of endothelial cells through the action of cells and their derivatives to promote endothelial cell survival, improve the microvascular network, and reduce tissue remodeling? Or can we promote the mesenchymal phenotypic transition of endothelial cells to enhance angiogenesis and anti-inflammatory and anti-fibrotic effects? There are many areas worth exploring in depth.

#### Clinical Application: Challenges and Opportunities from Laboratory To Clinic

Despite multiple clinical trials indicating that stem cell therapy holds potential for improving myocardial remodeling post-MI, its effects have not yet reached the ideal therapeutic goals. For example, BMMNCs have shown improvements in left ventricular function in some studies, but other studies have failed to replicate these results. This discrepancy may be related to factors such as cell source, processing methods, injection timing, and individual patient differences. Future research needs to further validate the efficacy and safety of cell therapy through multicenter, large-sample, and standardized randomized controlled trials, and explore standardized cell preparation and application protocols.

Moreover, the clinical application of stem cell therapy still faces many challenges that need to be addressed. First, the safety of cell products is one of the key issues. How to ensure the long-term safety of stem cell products in the human body, especially in terms of immune reactions and tumor formation risks, is a focus of future research. Second, optimizing therapeutic effects is another urgent issue. By optimizing cell dosage, injection methods, and treatment timing, the efficacy of stem cell therapy can be further improved, making it more advantageous in clinical applications. Finally, long-term follow-up studies are crucial for assessing the long-term effects of stem cell therapy and its impact on patients’ quality of life. Answers to these questions will provide important evidence for the clinical application of stem cell therapy.

## Conclusion

Cell therapy, as an emerging treatment for myocardial remodeling post-MI, holds great potential but still faces many challenges. Future research needs to delve into the mechanisms of cell therapy, optimize treatment strategies, and validate their efficacy and safety in clinical settings, starting from multiple aspects such as mechanism research, clinical application, new technology development, and combination therapies. Although the road ahead is long, with the continuous advancement of research, cell therapy is expected to bring new breakthroughs in the treatment of myocardial remodeling post-MI and offer more hope for patients.

## Data Availability

Not applicable.
